# Synthesis of Heteroleptic Amidinato Calcium Halides via the In‐Situ Grignard Addition Method (iGAM) Complementing the Metalation of Amidines

**DOI:** 10.1002/chem.202500210

**Published:** 2025-03-30

**Authors:** Simon Sengupta, Maximillian Broek, Phil Köhler, Matthias Westerhausen

**Affiliations:** ^1^ Institute of Inorganic and Analytical Chemistry Friedrich Schiller University Jena Humboldtstraße 8 D‐07743 Jena Germany

**Keywords:** amidinates, calciation, calcium, in‐situ grignard addition method, metalation reactions

## Abstract

The in‐situ Grignard addition method adds intermediately prepared organo alkaline‐earth metal halides onto a suitable substrate. Here, freshly rasped calcium and the carbodiimide R′─N═C═N─R′ (R′ = SiMe_3_, *i*Pr) are suspended in THF at room temperature and then an organyl halide R─X (X = Br, I) is added. Within several hours, intermediately formed R─Ca─X adds onto the carbodiimide yielding the corresponding thf adducts of the amidinato calcium halide {(R′N)_2_C─R}CaX. Suitable organyl halides besides aryl iodide are methyl and ethyl halide whereas bulkier isopropyl and *tert*‐butyl iodides give very low yields.

## Graphical abstract

The in‐situ generation of Grignard‐type organocalcium reagents via the reduction of alkyl and aryl halides in THF and their immediate use for subsequent addition onto carbodiimides allows the synthesis of hitherto unknown amidinates of calcium.

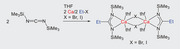

## 1 Introduction

The alkaline‐earth metals magnesium and calcium are rather inert and passivated, partly due to an oxide/hydroxide layer of commercially available granules, and direct metalation of H‐acidic compounds like amines and phosphines is impossible. Therefore, other strategies have been developed to produce calcium‐based metalation reagents whereas commercially available *n*‐butyllithium and dibutylmagnesium are convenient s‐block metal‐based reagents. The preparation of alkaline‐earth metal (Ae) bis[bis(trimethylsilyl)amides] (Ae(hmds)_2_) of calcium, strontium, and barium in the early 1990s^[^ ^1^ ^]^ allows homogeneous reaction conditions in anhydrous organic solvents such as ethers and aromatic hydrocarbons. With these compounds, metalation of diverse H‐acidic substrates is possible, depending on the *pK*_a_ values. Furthermore, the addition of Ae(hmds)_2_ onto benzonitrile and subsequent 1,3‐migration of a trimethylsilyl group, yielding mononuclear 1,3‐bis(trimethylsilyl)benzamidinates of the Ae metals,^[^ ^2^ ^]^ has been the initial step into a rich and successful amidinato alkaline‐earth metal chemistry especially of the heavier group 2 elements. The alkali metal (A) pendants A(hmds) react in a very similar manner leading to the formation of dimeric alkali metal 1,3‐bis(trimethylsilyl)benzamidinates.^[^ ^3^ ^]^

**Scheme 1:**
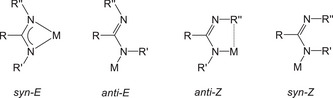
Coordination modes of amidinato ligands in metal complexes. In *syn‐E* amidinato compounds 1,3‐diazaallyl units with very similar C─N bonds are observed. In *anti‐E* and *anti‐Z* conformations the group R′ contains an additionally coordinated Lewis base. The *anti‐Z* conformation is stabilized via interaction with the substituent R′′.

Amidinates and related guanidinates^[^ ^4^ ^]^ besides β‐diketiminates^[^ ^5^ ^]^ are popular ligands not only in the chemistry of the heavier alkaline‐earth metals because these ligands are chemically very robust and in addition, their steric demand and hence shielding effect can be tuned and adopted to the reactivity of the metal‐containing functionality. Therefore, amidinates have been recognized quite early as pendants of bulky cyclopentadienides.^[^ ^6^ ^]^ Impressive examples, illustrating the protecting capability of bulky amidinates include bis(amidinato)dimagnesium(I) with a Mg─Mg bond^[^ ^7^ ^]^ and amidinato alkaline‐earth metal hydrides.^[^ ^8^ ^]^ The amidinato ligand can adopt four coordination modes that are depicted in Scheme 1. The *syn‐E* conformation is the preferred form for coordination at s‐block metals, however, the stabilization of heteroleptic amidinato calcium hydride requires the shielding of the Ca─H functionality by an *anti‐Z*‐amidinate allowing π‐interactions with the bulky aryl group R′′. In addition, the robustness of bis(amidinato)calcium has been utilized in catalysis^[^ ^9^ ^]^ and the application of volatile calcium precursor compounds for atomic layer deposition processes.^[^ ^10^ ^]^

**Scheme 2:**
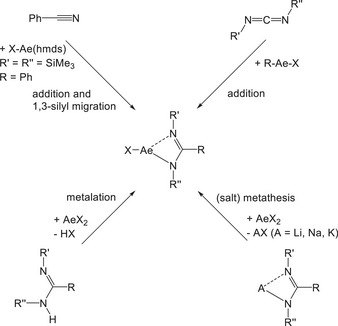
Preparative pathways for the synthesis of amidinato alkaline‐earth metal complexes.

The significance of the compound class of amidinato alkaline‐earth metal derivatives has led to the development of diverse strategies for their preparation. With Ae(hmds)_2_ at hand, addition onto benzonitrile as well as metalation of amidines are convenient protocols (Scheme 2). Another strategy is the addition of an alkaline‐earth metalorganic compound onto carbodiimides. Even bulky mesityl magnesium bromide adds onto sterically shielded Ar─C═N═C─Ar (Ar = Mes, Dipp) yielding dinuclear 1,3‐diaryl‐2‐mesityl‐1,3‐diazaallylmagnesium bromide with the halide ions in bridging positions.^[^ ^11^ ^]^ In organocalcium chemistry, intermediately formed calcium di(phenylacetylide) adds onto diisopropylcarbodiimide yielding dinuclear calcium bis[1,3‐diisopropyl‐2‐phenylethynyl‐1,3‐diazapropenide].^[^ ^12^ ^]^ Heteroleptic β‐diketiminato calcium 1,3‐diisopropyl‐2‐phenylethynyl‐1,3‐diazapropenide is accessible by a very similar strategy.^[^ ^13^ ^]^ Such a procedure has been applied also to prepare 1,3‐dimesitylbenzamidinates of the alkaline‐earth metals via addition of intermediately formed phenyl alkaline‐earth metal iodides onto bulky dimesitylcarbodiimide.^[^ ^14^ ^]^ Furthermore, alkaline‐earth metal amides and phosphides add smoothly onto carbodiimides, yielding guanidinates and 2‐phosphinylamidinates.^[^ ^15^ ^]^ However, the availability of Grignard‐type organometallics of the heavier alkaline‐earth metals limits this possibility because the synthesis of heavy Grignard reagents is related to diverse challenges due to the discrepancy between the inertness of the metal on the one hand and the high reactivity of the organometallics on the other. Nevertheless, calcium Grignard reagents gain tremendous interest due to a more polar Ca─C bond (comparable to RLi) compared to the Mg─C bond of classical Grignard reagents leading to an enhanced nucleophilicity of the calcium‐bound carbanion. Contrary to lithium, calcium can safely be handled in an anhydrous nitrogen atmosphere whereas an argon atmosphere is required when working with lithium. Other advantages are the environmental benign properties and the global abundance of this metal.^[^ ^16^ ^]^ However, the enormous reactivity of organocalcium compounds leads to side reactions like ether degradation and Wurtz‐type C─C coupling reactions besides others depending on the substrate.^[^ ^17^ ^]^ In addition, the aggressiveness of calcium‐based organometallics limits the use of bulky halogeno arenes precluding the straightforward formation of 2,4,6‐tri(*tert*‐butyl)phenyl^[^ ^18^ ^]^ and 2,4,6‐triphenylphenylcalcium organometallics^[^ ^19^ ^]^ due to dominating side reactions like intramolecular metalation, calcium–iodine exchange and redox reactions. The high reactivity also excludes the application of ethereal stock solutions and contrary to commercially available alkane stock solutions of *n*‐butyllithium and dibutylmagnesium, calcium‐based organometallics can only be prepared in ethereal solutions with satisfactory yields. Mesityl calcium halide shows not only a Schlenk‐type equilibrium but also slow conversion to yellow 3,5‐dimethylbenzylcalcium halide requiring low temperatures during preparation and handling of this arylcalcium Grignard reagent.^[^ ^20^ ^]^ In general, direct synthesis of organocalcium Grignard reagents is related to diverse challenges limiting high‐yield synthesis of alkylcalcium halides.^[^ ^21^ ^]^

**Scheme 3:**
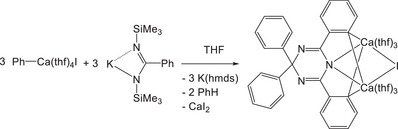
Unexpected reaction of phenyl calcium iodide with potassium 1,3‐bis(trimethylsilyl)benzamidinate yielding [4,4‐diphenyl‐2,6‐bis(1,2‐phenylene)‐1,3,5‐triazacyclohexa‐2,5‐diene‐1‐yl] dicalcium iodide via the reaction of [PhCa(thf)_4_I] with slowly liberated benzonitrile and potassium bis(trimethylsilyl)amide (K(hmds)).

In general, direct synthesis of organocalcium Grignard reagents is related to diverse challenges limiting high‐yield synthesis of alkylcalcium halides.^[^ ^21^ ^]^ The metathetical approach avoids the preparation of organocalcium compounds but requires the synthesis of alkali metal amidinates which react with calcium iodide functionalities yielding amidinato calcium complexes. Thus, heteroleptic 1,3‐diisopropylbenzamidinato calcium iodide can be prepared via the reaction of the lithium amidinate with CaI_2_.^[^ ^22^ ^]^ Homoleptic chiral benzamidinate complexes of the heavier alkaline‐earth metals are accessible via the reaction of potassium amidinates with group 2 iodides.^[^ ^23^ ^]^ However, during the metathesis reaction of phenyl calcium iodide with potassium 1,3‐bis(trimethylsilyl)benzamidinate, benzonitrile has been liberated very slowly under formation of K(hmds) yielding unexpected [4,4‐diphenyl‐2,6‐bis(1,2‐phenylene)‐1,3,5‐triazacyclohexa‐2,5‐diene‐1‐yl]dicalcium iodide as depicted in Scheme 3.^[^ ^24^ ^]^

To circumvent the challenging isolation of calcium Grignard reagents, the in‐situ Grignard addition method (iGAM) has been chosen.^[^ ^25^ ^]^ In this procedure, the intermediately formed organocalcium reagent is immediately consumed by a substrate as has been demonstrated for the addition reaction of RCaX onto imines yielding amido calcium complexes.^[^ ^26^ ^]^ Contrary to amido magnesium halides (Hauser bases),^[^ ^27^ ^]^ the amido calcium halides undergo a Schlenk‐type ligand scrambling with the homoleptic calcium bis(amides) and calcium dihalide being strongly favored.^[^ ^28^ ^]^ Here we produced organocalcium halides and trapped these reagents immediately with carbodiimides yielding amidinato calcium complexes.

## 2 Results and Discussion

### 2.1 Synthesis

**Scheme 4:**
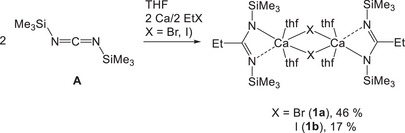
Synthesis of heterolptic 1,3‐bis(trimethylsilyl)‐2‐ethylamidinatocalcium bromide (**1a**) and iodide (**1b**) in THF solution via the in‐situ Grignard addition method (iGAM). The yields refer to isolated crystalline material, conversion rates are given in Table 1 (entries 2 and 3).

In a general procedure for the use of intermediately formed alkyl‐ or arylcalcium halide, slight excess of commercially available calcium granules or freshly rasped calcium (≈1.2 equiv.) and bis(trimethylsilyl)carbodiimide (**A**) were suspended at room temperature in THF. Then bromo‐ or iodoethane (1 equiv.) was added and the reaction mixture stirred for a few hours as depicted in Scheme 4. Within several minutes, the mixture turned cloudy due to precipitation of calcium halide. Removal of the precipitate (calcium halide and excess of calcium metal) and recrystallization from a solvent mixture of THF and pentane yielded colorless [{(Me_3_SiN)_2_C─Et}Ca(thf)_2_(μ─X)]_2_ with X = Br (**1a**) and I (**1b**). Liberation of propionitrile from these complexes was not observed and a degradation of this silylated amidinate as had been observed during the reaction of K{Ph─C(NSiMe_3_)_2_} with PhCaI (Scheme 3)^[^ ^24^ ^]^ did not occur.

**Scheme 5:**
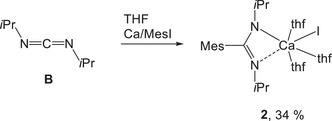
Synthesis of heteroleptic 1,3‐diisopropyl‐2‐mesitylamidinatocalcium iodide (**2**) in THF solution via the in‐situ Grignard Addition Method (iGAM). The yield refers to isolated crystalline material, the conversion rate is given in Table 1 (entry 13).

As mentioned earlier, mesityl calcium halide slowly vanishes and forms a benzyl derivative.^[^ ^20^ ^]^ Therefore, we studied the in‐situ addition of mesityl calcium iodide onto diisopropylcarbodiimide (**B**) as depicted in Scheme 5. This carbodiimide and calcium granules were suspended in THF at room temperature and then mesityl iodide was added. After a few hours, all solids were removed by filtration and the volume of the filtrate was reduced in vacuo. Within a few days, colorless crystals of [{(*i*PrN)_2_C─Mes}Ca(thf)_3_I] (**2**) precipitated at room temperature. This observation verified that the addition of calcium organometallics was much faster than the formation of the 3,5‐dimethylbenzyl substituent.

To probe the scope of the iGAM of carbodiimides, we varied the added alkyl‐ or arylhalide. Freshly rasped calcium (6–7.5 mmol, 1.2–1.5 equiv.), THF (10 mL) and bis(trimethylsilyl)carbodiimide (**A**) or diisopropylcarbodiimide (**B**) (5.0 mmol, 1.0 equiv.) were placed in a Schlenk tube. Alkyl/aryl halide RX (5.0 mmol, 1.0 equiv.) was added, and the reaction mixture was stirred at room temperature for 5–16 h. If the temperature of the reaction mixture increased, it was cooled with ice to limit ether degradation and Wurtz‐type coupling side reactions. The conversion of the carbodiimide was determined by titration of a hydrolyzed aliquot with sulfuric acid (0.1 n) against phenolphthalein. The results are summarized in Table 1 regardless of the mono‐ or dinuclear appearance of the amidinato calcium compounds. In THF solution, a Schlenk‐type equilibrium was operative leading to two sets of ^13^C and ^29^Si NMR signals. For the compounds **1a** (entry 3) and **1b** (entry 2) we were able to determine the molar masses of both species which are monomeric in THF solution. In both cases, the major components were the heteroleptic amidinato calcium halides. The 1,3‐diisopropylamidinato calcium congeners were monomeric in THF, too, but the assignment to homoleptic calcium bis(amidinate) and heteroleptic amidinato calcium halide was less distinct due to overlapping NMR signals.

**Table 1:** Formation of amidinates via the iGAM by addition of intermediately from Ca and RX in THF formed alkyl‐ or arylcalcium halide onto the carbodiimides R′─N═C═N─R′ (R′ = SiMe_3_, **A** or R′ = *i*Pr, **B**).

Entry	R′N═C═NR′	RX	Conv.	*δ*(^13^C_NCN_)_Ca_^[^ ^a^ ^]^	*δ*(^13^C_NCN_)_H_^[^ ^b^ ^]^
1	**A**	MeI	40%	‐	‐
2	**A**	EtI	>95%	183.0/**182.9**	168.2
3	**A**	EtBr	>95%	183.0/**182.7**	168.2
4	**A**	*i*PrI	2%	‐	‐
5	**A**	PhI	84%	‐	‐
6	**B**	MeI	91%	**170.0**/168.6	154.4
7	**B**	EtI	80%	**174.2**/172.8	158.4
8	**B**	EtBr	>95%	**174.0**/172.8	158.4
9	**B**	*i*PrI	13%	‐	‐
10	**B**	*t*BuBr	5%	‐	‐
11	**B**	1‐AdBr	2%	‐	‐
12	**B**	PhI	>95%	**174.1**/173.2	156.7
13	**B**	MesI	87%	**172.9**/172.8	163.9

^[a]^ ^13^C chemical shift of the NCN fragment of the calcium‐bound amidinato ligands; the major component is given with bold numbers.

^[b]^ ^13^C NMR shifts for the amidines, i.e., the protolyzed calcium complexes.

In entries 1 to 5, carbodiimide **A** was derivatized. For bromo‐ and iodoethane the conversion was quantitative (entries 2 and 3). The lower conversion rate using iodomethane assumedly resulted from the low solubility of highly aggregated methyl calcium iodide and dimethylcalcium^[^ ^29^ ^]^ covering the calcium particles thus decelerating the calcium Grignard formation (entry 1). Bulkier 2‐iodopropane was not a suitable substrate in the iGAM of **A** containing bulky trimethylsilyl groups (entry 4). Iodobenzene was a suitable substrate yielding the already known 1,3‐bis(trimethylsilyl)benzamidinato calcium compounds (entry 5). Addition of organocalcium halide should be faster onto less shielded diisopropylcarbodiimide (**B**). Whereas the application of iodomethane (entry 6) and iodo‐/bromoethane (entries 7 and 8) straightforwardly yielded the 1,3‐diisopropyl‐2‐alkylamidinates, increasing steric hindrance and the use of 2‐iodopropane (isopropyliodide, entry 9), 2‐bromo‐2‐methylpropane (*tert*‐butyliodide, entry 10) and 1‐bromoadamantane (entry 11) lowered the conversion rate significantly. The use of iodobenzene (entry 12) allowed a quantitative conversion rate whereas enhancement of steric hindrance using mesityl iodide lowered the conversion rate again (entry 13). In addition to steric requirements, fast ether cleavage side reactions could compete with the iGAM, reducing the yield. It is well‐known that the half‐lives of BuLi in THF solutions significantly decreased from *n*BuLi (107 min at +20 °C) over *s*BuLi (78 min at −20 °C) to *t*BuLi (42 min at −20 °C).^[^ ^30^ ^]^ Considering that Li and Ca have the same Pauling electronegativity value of 1.0, comparable aggressiveness toward THF might be expected.

**Scheme 6:**
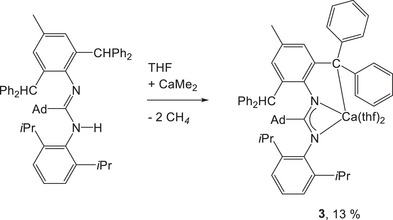
Synthesis of 1‐(2,6‐diisopropylphenyl)‐2‐(1‐adamantyl)‐3‐(2‐diphenylmethyl‐4‐methyl‐6‐diphenylmethanidophenyl)amidinato calcium (**3**) in THF solution via twofold metalation of the corresponding amidine. The yield refers to isolated crystalline material.

For these reasons, calcium compounds with bulky amidinato ligands, especially containing branched alkyl groups at the carbon atom of the 1,3‐diazaallyl moiety, had to be prepared by alternative procedures like metalation of amidines. Hence, we metalated the sterically congested 1‐(2,6‐diisopropylphenyl)‐2‐(1‐adamantyl)‐3‐[2,6‐bis(diphenylmethyl)‐4‐methylphenyl]amidine with sparingly soluble dimethylcalcium in THF, yielding the corresponding amidinato calcium compound **3** according to Scheme 6. The amidine was doubly deprotonated at the amidine functionality and at the triarylmethyl unit. This latter deprotonation is a consequence of increasing acidity of the methyl subunit with increasing number of aryl substituents (*pK*_a_ values for CH_4−_ *_n_* Ph*_n_*: *n* = 0: 40, *n* = 1: 37, *n* = 2: 35, and *n* = 3: 33).^[^ ^31^ ^]^ Metalation of triphenylmethane with dialkylcalcium in THF and the synthesis of triphenylmethyl alkaline‐earth metal complexes had successfully been performed earlier, using dibenzyl alkaline‐earth metal reagents.^[^ ^32^ ^]^ The sterically crowded environment led to a penta‐coordinate calcium atom.

Such bulky N‐bound 2,6‐bis(diphenylmethyl)‐4‐tolyl substituents had been applied earlier to shield amidinato alkaline‐earth metal hydride functionalities of magnesium, calcium, and strontium.^[^ ^8^ ^]^ In addition, primary 2,6‐bis(diphenylmethyl)‐4‐tolylamides of the alkaline‐earth metals were accessible.^[^ ^28^, ^33^ ^]^ This *N*‐bound substituent allowed also the synthesis of a donor‐free mononuclear β‐diketiminato calcium iodide with a terminally bound halide.^[^ ^34^ ^]^ Calciation of one *ortho*‐diphenylmethyl sidearm had been observed earlier during the metalation of overcrowded *N*‐triphenylsilyl‐*N*‐[2,6‐bis(diphenylmethyl)‐4‐isopropylphenyl]amine with diphenylcalcium, which was intermediately prepared via transmetalation of diphenylmercury with calcium metal.^[^ ^35^ ^]^

The *N*,*N*′‐bis(trimethylsilyl)‐substitution leads to a low field‐shifted carbon resonance of the diazaallyl unit compared to the 1,3‐diisopropylamidinato ligands (Table 1). Increasing heteropolar bonding character between the alkaline‐earth metal and this amidinato ligands in ether adducts of {(Me_3_SiN)_2_C─Ph}_2_Ae causes a slight highfield‐shifted resonance of the carbon atom of the 1,3‐diazaallylic system (δ(^13^C_NCN_): Be 182.7, Mg 181.8, Ca 180.3; Sr 179.7, and Ba 179.1 ppm).^[^ ^2^ ^]^ However, the influence of the alkaline‐earth metal on the ^13^C_NCN_ chemical shift is very small in agreement with a mainly ionic bonding situation. Consequently, exchange of one amidinato ligand by a halide ion also leads only to a slight shift of the ^13^C_NCN_ NMR resonance.

For the 1,3‐diisopropylamidinato calcium compounds, the C‐substitution at the 1,3‐diazaallyl backbone plays a very minor role and the *δ*(^13^C_NCN_) values are distributed in a narrow range around 172 ppm and the alkaline‐earth metal itself has a small influence. In the CAAC‐stabilized beryllium complex [{(*i*PrN)_2_C─Ph}Be(CAAC)Cl]_2_ a chemical shift of 173.9 ppm has been observed.^[^ ^36^ ^]^ The magnesium congeners show rather similar NMR parameters and even enhanced steric demand of the aryl group at the carbon atom of the diazaallyl unit leads to very small variation of the ^13^C_NCN_ NMR chemical shifts as observed, e.g., for [{(*i*PrN)_2_C─Mes}Mg(OEt_2_)(μ─Br)]_2_ (173.9 ppm),^[^ ^11^ ^]^ [{(*i*PrN)_2_C─Ar*}Mg(thf)_2_I] with Ar* = C_6_H_3_‐2,6‐(C_6_H_4_‐4‐*t*Bu)_2_ (173.3 ppm),^[^ ^37^ ^]^ and [{(*i*PrN)_2_C─Ar*}_2_Mg] with Ar* = C_6_H_3_‐2,6‐Mes_2_ (170.1 ppm).^[^ ^37^ ^]^ Strontium and barium derivatives with the 1,3‐diisopropyl‐1,3‐diazaallyl ligand are hitherto unknown.

### 2.2 Molecular Structures

**Figure 1:**
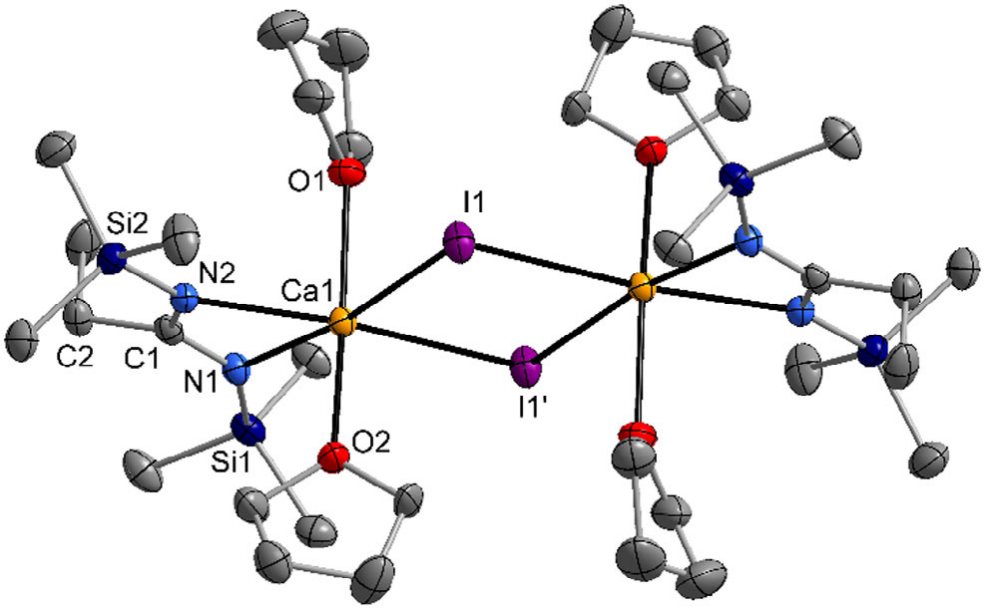
Molecular structure and atom labeling scheme of **1b**. Ellipsoids represent a probability of 50%, hydrogen atoms are neglected for clarity reasons. Symmetry‐equivalent atoms (1−*x*, 1−*y*, 1−*z*) are marked with an apostrophe. Selected bond lengths of **1b** [of **1a** in brackets] (pm): Ca1─N1 238.7(2) [239.3(2)], Ca1─N2 238.9(2) [239.9(2)], Ca1─I1 315.50(6) [Ca1─Br1 291.58(6)], Ca1─I1′ 315.61(6) [Ca1─Br1′ 291.32(6)], Ca1─O1 239.7(2) [240.2(2)], Ca1─O2 234.0(2) [234.6(2)], C1─N1 133.0(3) [132.3(3)], C1─N2 134.0(3) [133.7(3)], C1─C2 152.0(3) [152.3(3)], N1─Si1 171.4(2) [170.9(2)], N2─Si2 170.9(2) [170.7(2)]. Bond angles (deg): N1─Ca1─N2 57.60(7) [57.15(6)], Ca1─N1─C1 91.5(2) [91.8(1)], C1─N1─C2 119.0(2) [119.0(2)], Ca1─N2─C1 91.1(2) [91.2(1)].

The centrosymmetric complexes [{(Me_3_SiN)_2_C─Et}Ca(thf)_2_(μ─X)]_2_ with X = Br (**1a**) and I (**1b**) crystallize isomorphous in the monoclinic space group *P*2_1_/c. Molecular structure and atom labeling scheme of **1b** are depicted in Figure 1, compound **1a** is shown in Figure S27, Supporting Information. The calcium atoms are in distorted octahedral environments due to small endocyclic N1─Ca1─N2 bond angles. The Ca1─N1 and Ca1─N2 bond lengths are very similar due to comparable chemical environments because both nitrogen atoms are trans‐positioned to a μ‐bridging iodine atom. The 1,3‐diazaallyl fragments N1─C1─N2 show charge delocalization with C1─N1 and C1─N2 bond lengths between typical C─N single (138 pm) and double bonds (128 pm).^[^ ^38^ ^]^ A very similar structure has been observed for [{(MesN)_2_C─Ph}Ca(thf)_2_(μ─I)]_2_ with hexa‐coordinate calcium atoms.^[^ ^14^ ^]^

The structural parameters of the amidinato ligand are rather similar to those of [{(Me_3_SiN)_2_C─Ph}_2_Ca(thf)_2_] due to the lack of interaction between the phenyl and 1,3‐diazaallyl π‐systems.^[^ ^2c^ ^]^ The relatively short Ca─O bonds verify absence of significant intramolecular strain in these compounds.

**Figure 2:**
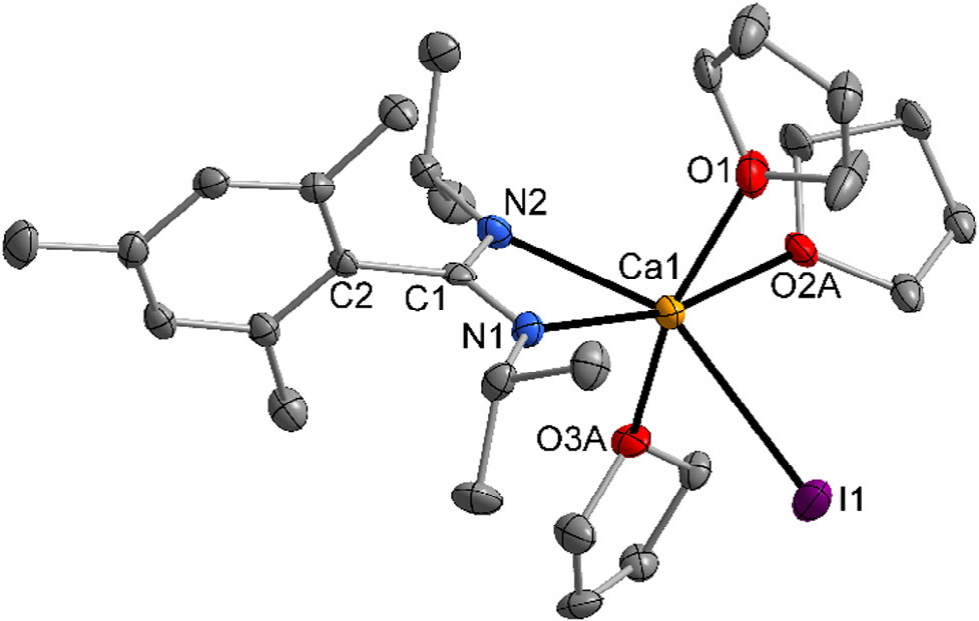
Molecular structure and atom labeling scheme of **2**. The ellipsoids represent a probability of 40%, hydrogen atoms and minor‐occupancy orientations of disordered thf ligands are omitted for clarity reasons. Selected bond lengths (pm): Ca1─N1 238.7(6), Ca1─N2 242.2(6), Ca1─I1 309.6(2), Ca1─O1 237.5(6), Ca1─O2A 241.7(9), Ca1─O3A 239.8(12), C1─N1 134.3(9), C1─N2 131.5(10), C1─C2 152.4(10). Bond angles (deg.): N1─Ca1─N2 56.4(2), Ca1─N1─C1 93.4(4), Ca1─N2─C1 92.5(4), N1─C1─N2 117.5(7).

The molecular structure and atom labeling scheme of mononuclear [{(*i*PrN)_2_C─Mes}Ca(thf)_3_I] (**2**) is depicted in Figure 2,. The structure is very similar to the less strained phenyl‐substituted complex [{(*i*PrN)_2_C─Ph}Ca(thf)_3_I].^[^ ^22^ ^]^ The calcium center shows the preferred octahedral coordination which is heavily distorted due to the small bite of the amidinato ligand. The Ca1─N1 and Ca1─N2 distances are slightly different due to the different chemical environments of N1 and N2 and packing effects in the crystal. For steric reasons, the mesityl group is oriented nearly perpendicular to the 1,3‐diazaallyl plane which hinders the interaction between these π‐systems. This finding is verified by a long C1─C2 bond.

The comparable addition of mesitylmagnesium bromide onto diisopropylcarbodiimide in diethyl ether yields centrosymmetric dinuclear [{(*i*PrN)_2_C─Mes}Mg(OEt_2_)(μ─Br)]_2_ with penta‐coordinate magnesium centers and bridging bromide ions.^[^ ^11^ ^]^ The Mg─Br distances in the inner Mg_2_Br_2_ ring differ by ≈11 pm leading to different Mg─N bond lengths of 207.6(2) and 211.4(2) pm. High donor strength of the ether base thf and increasing softness of the halogen atom lower the negative charge on the nitrogen atoms, and in addition, steric requirements disfavor the formation of a dinuclear structure of **2** as it has been observed for **1a** and **1b**. A comparable mononuclear molecule has been authenticated by X‐ray crystallography also for [{(*i*PrN)_2_C─Ph}Ca(thf)_3_I].^[^ ^22^ ^]^ Crystallization of mononuclear or dinuclear structures of heteroleptic amidinato calcium halides is hard to predict and small changes in steric requirements and crystallization conditions seem to determine the outcome. It is remarkable that the heteroleptic amidinato calcium halides are stable in solution which is contrary to the amido calcium halides where a Schlenk‐type equilibrium is shifted in favor of the homoleptic calcium bis(amides) and calcium halide.

In amidinato alkaline‐earth metal complexes with medium sized N‐bound trimethylsilyl and isopropyl substituents, formation of the *syn‐E*‐isomer is observed. Charge delocalization leads to very similar C─N bond lengths within the 1,3‐diazaallyl system (Table 2). Lack of steric repulsion allows a symmetric coordination at the metal ion with very similar Ae─N distances. Very bulky groups at the backbone of the 1,3‐diazaallyl moiety with demanding N‐bound aryl groups enforce the formation of *anti‐Z* isomeric amidinates with different C─N bond lengths in the 1,3‐diazaallylic substructure, stabilized by π‐interactions between the aryl group and the alkaline‐earth ion.^[^ ^8^, ^39^ ^]^ Asymmetric coordination of amidinato ligands at calcium is also observed when different N‐bound substituents are present, especially if one of the side arms contains a Lewis basic donor atom.^[^ ^40^ ^]^ Structural parameters of selected examples of alkaline‐earth metal‐bound amidinato ligands of the type {(RN)_2_C─R′}^−^ with R = SiMe_3_ and *i*Pr in beryllium, magnesium, and calcium complexes are listed in Table 2. The small beryllium atoms have tetrahedral coordination spheres, distorted due to the small bite of the bidentate amidinato ions. The calcium atoms prefer octahedral environments whereas the coordination number of magnesium strongly depends on the steric requirements of the amidinato ligand and hence, coordination numbers between 4 and 6 are observed. Generally, both nitrogen bases show very similar Ae─N distances except Mg complexes with penta‐coordinate metal atoms. In these trigonal bipyramidal coordination, the nitrogen bases are in significantly different environments with one N base in an apical and the other in an equatorial position. This asymmetric binding mode does not disturb delocalization of charge within the 1,3‐diazaallyl system.

**Table 2:** Comparison of selected structural parameters of the alkaline‐earth metal‐bound amidinato ligands {(R′N)_2_C─R}^−^ (R′ = SiMe_3_, *i*Pr) in homo‐ and heteroleptic beryllium, magnesium, and calcium complexes (bond lengths [pm] and angles [deg.]).

Compound	C.N.(Ae)	Ae─N1	Ae─N2	ΔAe─N1/2	C_NCN_─N1	C_NCN_─N2	ΔC_NCN_─N1/2	N1─C_NCN_─N2	Ref.
[{(Me_3_SiN)_2_C─Ph}_2_Be]^[^ ^a^ ^]^	4	172.9(6)	173.0(6)	0.1	132.9(5)	133.2(5)	0.3	112.9(3)	^[^ ^2a^ ^]^
[{(Me_3_SiN)_2_C─Ph}_2_Mg(N≡C─Ph)]	5	210.9(3)	213.8(3)	2.9	132.3(5)	132.1(5)	0.2	118.8(3)	^[^ ^2b^ ^]^
[{(Me_3_SiN)_2_C─Ph}_2_Ca(thf)_2_]	6	242.4(2)	243.8(2)	1.4	131.7(4)	132.2(4)	0.5	120.7(3)	^[^ ^2c^ ^]^
[{(Me_3_SiN)_2_C─Et}Ca(thf)_2_(μ─Br)]_2_ (**1a**)	6	239.3(2)	239.9(2)	0.6	132.3(3)	133.7(3)	1.4	119.0(2)	Here
[{(Me_3_SiN)_2_C─Et}Ca(thf)_2_(μ─I)]_2_ (**1b**)	6	238.7(2)	238.9(2)	0.2	133.0(3)	134.0(3)	1.0	119.0(2)	Here
[{(*i*PrN)_2_C─Ph}Be(CAAC)Cl]	4	178.0(3)	177.9	0.1	133.4(3)	131.7	1.7	110.8	^[^ ^36^ ^]^
[{(*i*PrN)_2_C─Ar*}_2_Mg]^[^ ^c^ ^]^	4	204(1)	204(1)	0	131(2)	131(2)	0	111(1)	^[^ ^37^ ^]^
[{(*i*PrN)_2_C─Mes}Mg(OEt_2_)(μ─Br)]_2_	5	211.4(2)	207.6(2)	3.8	132.7	133.1	0.4	114.0(2)	^[^ ^11^ ^]^
[{(*i*PrN)_2_C─Ar*}Mg(thf)_2_I]^[^ ^b^ ^]^	5	208.5(6)	214.0(6)	5.5	133.9(8)	133.2(8)	0.7	114.5(6)	^[^ ^37^ ^]^
[{(*i*PrN)_2_C─Ph}_2_Mg(thf)_2_]	6	216.8(6)	216.1(6)	0.7	132.5(10)	133.8(10)	1.3	114.5(7)	^[^ ^41^ ^]^
[{(*i*PrN)_2_C─C≡C─Ph}_2_Ca]_2_^[^ ^d^ ^]^	6	238.2(2)	237.0(3)	1.2	133.3(4)	132.7(4)	0.6	117.9(3)	^[^ ^12^ ^]^
[{(*i*PrN)_2_C─PPh_2_}_2_Ca(thf)_2_]^[^ ^a^ ^]^	6	242.1(2)	243.2(2)	1.1	132.1(3)	133.3(3)	1.2	116.3(2)	^[^ ^15b^ ^]^
[{(*i*PrN)_2_C─Ph}Ca(thf)_3_I]	6	238.3(11)	236.3(13)	2.0	135.0	132.8	2.2	116.4	^[^ ^22^ ^]^
[{(*i*PrN)_2_C─Mes}Ca(thf)_3_I] (**2**)	6	238.7(6)	242.2(6)	3.5	134.3(9)	131.5(10)	2.8	117.5(7)	Here

^[a]^ Only structural data of one ligand listed.

^[b]^ Ar* = C_6_H_3_‐2,6‐(C_6_H_4_‐4‐*t*Bu)_2_.

^[c]^ Ar* = C_6_H_3_‐2,6‐Mes_2_; rather poor structural data due to a two‐site disordering of the mesityl groups.

^[d]^ Only structural data of one terminal ligand listed.

**Figure 3:**
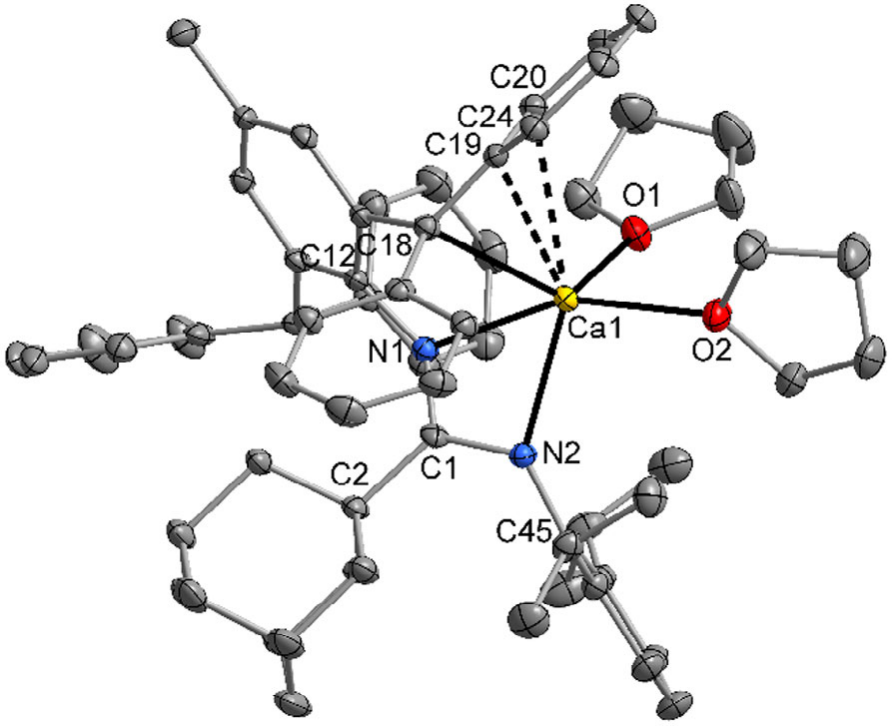
Molecular structure and atom labeling scheme of **3**. The ellipsoids represent a probability of 50%, the intercalated THF molecule and all hydrogen atoms are neglected for clarity reasons. Selected bond lengths (pm): Ca1─N1 235.2(2), Ca1─N2 234.7(2), Ca1─O1 234.3(2), Ca1─O2 235.4(2), Ca1─C18 262.4(2), Ca1─C19 276.1(2), Ca1─C20 282.7(2), C1─N1 133.6(2), C1─N2 135.1(3), C1─C2 154.5(3), N1─C12 141.7(2), N2─C45 142.0(2). Bond angles (deg.): N1─Ca1─N2 56.47(6), Ca1─N1─C1 94.0(1), Ca1─N2─C1 93.8(1), N1─C1─N2 111.7(2), Ca1─C18─C19 79.7(1), C13─C18─C19 116.9(2), C13─C18─C25 117.9(2), C19─C18─C25 122.0(2).

The molecular structure and atom labeling scheme of the bis(tetrahydrofuran) adduct of 1‐(2,6‐diisopropylphenyl)‐2‐(1‐adamantyl)‐3‐(2‐diphenylmethyl‐4‐methyl‐6‐diphenylmethanidophenyl)amidinato calcium (**3**) are depicted in Figure 3. The crowded environment of the calcium center leads to a coordination number of five. The coordination polyhedron lies with a *τ* factor of 0.46 between a square pyramidal and a trigonal bipyramidal structure (*τ* = (*α* − *β*)/60 with *α* as largest and *β* as second largest X─Ca1─X bond angle and with *τ* = 0 for an ideal square pyramid and *τ* = 1 for an undistorted trigonal bipyramid).^[^ ^42^ ^]^ The carbanionic C18 atom is coordinated by the three carbon atoms of the attached aryl groups in a nearly trigonal‐planar fashion with an angle sum of 356.8°. The diphenyl‐C18 fragment is oriented perpendicular to the N1‐bound aryl ring leading to a typical C13─C18 single bond (151.0(3) pm) whereas the C18─C19 (144.9(3) pm) and C18─C25 bonds (146.3(3) pm) are significantly shorter, verifying charge delocalization into these phenyl rings. The calcium atom binds side‐on to the carbon atoms C18, C19, and C20. A comparable side‐on coordination of calcium onto the benzylic as well as ipso‐ and ortho‐carbon atoms has been observed earlier for [(thf)_2_Ca{Ph─C(SiMe_3_)_2_}].^[^ ^43^ ^]^ Despite this third binding site the Ca1─N1 and Ca1─N2 bond lengths are very similar as expected for an amidinato calcium complex. Steric repulsion between the adamantyl and the bulky aryl groups enforces a small N1─C1─N2 bond angle of the 1,3‐diazaallyl unit.

1,3‐Diarylamidinato calcium compounds with tridentate amidinato anions have already been synthesized earlier and are often tested in catalysis.^[^ ^44^ ^]^ In such complexes, steric shielding of the calcium center can be tuned and adapted to the application in organocalcium chemistry.

## 3 Conclusions

The iGAM is an appropriate procedure for the synthesis of amidinato calcium halides in an ethereal solvent, preferably THF. A mandatory precondition for the suitability of iGAM is an addition of R─Ca─X across the unsaturated polar multiple bond which must be much faster than possible side reactions with the solvent (ether degradation) and Wurtz‐type C─C coupling via a metathetical reaction of R─Ca─X with still present organyl halide. Screening of the appropriateness of alkyl halides of the type Me_3−_ *_n_* H*_n_* C─X shows the preferred application of ethyl halides (*n* = 2). Methyl iodide leads to formation of methyl calcium iodide (*n* = 3) and also via a Schlenk‐type equilibrium to dimethylcalcium and calcium iodide. However, methyl calcium complexes are highly aggregated and only sparingly soluble,^[^ ^29^ ^]^ leading to precipitation onto the calcium pieces and deceleration of the Grignard‐type reaction. Higher branched alkyl halides like isopropyl (*n* = 1) and *tert*‐butyl halide (*n* = 0) give very low yields in this iGAM for two reasons. The fast kinetically controlled addition of these alkylcalcium halides is hampered by steric hindrance and side reactions can compete with the envisioned iGAM. In addition, aggressiveness of the carbanion increases from methyl (*n* = 3) over ethyl (*n* = 2) and isopropyl (*n* = 1) to *tert*‐butyl (*n* = 0) as has already been recognized for the lithium congeners leading to a significantly enhanced tendency to cleave ethers. Formation of formamidinates requires the addition of a calcium hydride functionality across the carbodiimide, however, the reaction of calcium with HX yields dihydrogen and calcium halide. As has been demonstrated earlier, calcium hydride moieties must be heavily shielded and then an addition onto carbodiimides is feasible.^[^ ^45^ ^]^

Phenyl and mesityl halides represent suitable substrates for the iGAM. Even though, mesityl calcium halide tends to rearrange slowly to 3,5‐dimethylbenzylcalcium halide, the addition onto diisopropylcarbodiimide is much faster than this rearrangement and 1,3‐diisopropyl‐2‐mesitylamidinato calcium halide can be prepared with good yield. Bulkier 2,4,6‐tri(*tert*‐butyl)phenyl iodide is completely inapplicable due to a fast degradation cascade of intermediately formed 2,4,6‐tri(*tert*‐butyl)phenyl calcium halide finally yielding calcium halide and the C─C coupling product 2,5‐bis[3,5‐di(*tert*‐butyl)phenyl]‐2,5‐dimethylhexane.

Contrary to amidocalcium halides, which show the strongly favored formation of homoleptic calcium di(amides) and calcium halide via a Schlenk‐type ligand exchange, heteroleptic amidinato calcium halides can be isolated and recrystallized. In dimeric complexes the halide ions occupy bridging positions and distorted octahedral coordination spheres of the calcium centers are saturated by ligated thf molecules.

The iGAM is a powerful tool complementing the metalation of H‐acidic compounds with in‐situ generated calcium Grignard reagents (iGMM) or with dialkylcalcium derivatives to expand and support a vivid development of organocalcium chemistry.

## 4 Experimental Section

### General Information

All manipulations were carried out under an inert nitrogen atmosphere using standard Schlenk techniques, if not otherwise noted. The solvents were dried over KOH and subsequently distilled over sodium/benzophenone under a nitrogen atmosphere prior to use. All substrates were purchased from Alfa Aesar, abcr, Sigma Aldrich or TCI and used without further purification. Calcium was freshly rasped prior to use. Dimethylcalcium^[^ ^29^ ^]^ and 1‐(2,6‐diisopropylphenyl)‐2‐(1‐adamantyl)‐3‐[2,6‐bis(diphenylmethyl)‐4‐methylphenyl]amidine^[^ ^8a^ ^]^ were prepared according to published protocols. The yields given are not optimized. The purity of the compounds was verified by NMR spectroscopy. Deuterated solvents were dried over sodium, distilled, degassed, and stored under nitrogen over sodium. ^1^H, ^13^C{^1^H}, ^29^Si{^1^H}‐DEPT NMR spectra were recorded on Bruker Fourier 300 and Bruker Avance III 400 (PA BBO 400S1 BB‐H‐D05 Z probehead, SampleXpress sample changer) spectrometers. Chemical shifts are reported in parts per million relatively to SiMe_4_ as an external standard referenced to the solvents residual proton signal using the *xiref* AU program for ^13^C NMR spectra. ASAP‐HSQC‐spectra were recorded using the published pulse sequences.^[^ ^46^ ^]^ DOSY NMR spectra were measured using the convection compensated dstebpgp3 s standard pulse sequence. Molar masses in solution were calculated using the Stalke‐ECC‐DOSY method.^[^ ^47^ ^]^ The microanalysis gave no reliable CHN data due to loss of ligated ether molecules during handling, due to carbonate formation and due to formation of silicon carbide and nitride for trimethylsilyl‐containing samples during combustion. The single‐crystal X‐ray intensity data were collected on a Bruker–Nonius Kappa‐CCD diffractometer equipped with a Mo‐Kα IµS microfocus source and an Apex2 CCD detector, at *T* = 120 K. The crystal structures were solved with SHELXT‐2018/3^[^ ^48^ ^]^ and refined by full matrix least‐squares methods on *F* ^2^ with SHELXL‐2018/3,^[^ ^49^ ^]^ using the Olex2 1.5 environment.^[^ ^50^ ^]^ Multi‐scan absorption correction was applied to the intensity data.^[^ ^51^ ^]^ Restraints were applied on the interatomic distances and/or anisotropic displacement parameters for disordered groups (RIGU and SADI commands in ShelXL).^[^ ^49^ ^]^

### [(thf)_2_Ca{EtC(N─SiMe_3_)_2_}(μ─Br)]_2_ (**1a**)

Freshly rasped calcium (5.8 mmol, 1.2 equiv.) and bis(trimethylsilyl)carbodiimide (4.8 mmol, 1.0 equiv.) were suspended in THF (10 mL). Ethyl bromide (4.8 mmol, 1.0 equiv.) was added, and the reaction mixture was stirred at room temperature for 6 h (conversion: >95 %). The solution was separated from the colorless solid and concentrated under reduced pressure. The residue was re‐dissolved in *n*‐pentane (10 mL) and stored at −20 °C overnight, yielding colorless crystals. The crystals were collected by filtration and dried carefully in vacuo (yield: 1.08 g, 1.13 mmol, 46%). ^1^H NMR (400 MHz, [D_8_]THF, 297 K) δ (ppm) = 3.64–3.59 (m, 16H, THF), 2.27 (q, 4H, CH_2_), 1.79–1.74 (m, 16H, THF), 1.13 (t, 6H, CH_3_), 0.08–0.06 (s, 36H, TMS); ^13^C{^1^H} (101 MHz, [D_8_]THF, 297 K) δ (ppm) = 183.0/**182.7** (N*C*N), 68.2 (THF), 35.7/**35.3** (CH_2_), 26.4 (THF), 14.4/14.4 (CH_3_), 3.5/**3.3** (TMS); ^29^Si{^1^H} DEPT‐NMR (79.5 MHz, [D_8_]THF, 297 K) δ (ppm) = −11.2/**−12.5/−13.0**; IR (ATR, ν [cm^−1^]) = 3250 (w), 2953 (m), 2881 (m), 1682 (w), 1645 (w), 1605 (w), 1468 (m), 1446 (m), 1405 (w), 1253 (m), 1240 (m), 1193 (w), 1071 (m), 1040 (m), 918 (w), 825 (s), 748 (m), 666 (m).

### [(thf)_2_Ca{EtC(N─SiMe_3_)_2_}(μ─I)]_2_ (**1b**)

Freshly rasped calcium (7.5 mmol, 1.5 equiv.) and bis(trimethylsilyl)carbodiimide (5.0 mmol, 1.0 equiv.) were suspended in THF (10 mL). Ethyl iodide (5.0 mmol, 1.0 equiv.) was added, and the reaction mixture was stirred at room temperature for 5 h (conversion: >95 %). The solution was separated from the colorless solid and concentrated under reduced pressure. The residue was re‐dissolved in *n*‐pentane (12 mL) and stored at −20 °C overnight, yielding colorless crystals. The crystals were collected by filtration and dried carefully in vacuo (yield: 0.46 g, 0.44 mmol, 17%). ^1^H NMR (400 MHz, [D_8_]THF, 297 K) δ (ppm) = 3.64–3.59 (m, 16H, THF), 2.27 (q, 4H, CH_2_), 1.80–1.74 (m, 16H, THF), 1.12 (t, 6H, CH_3_), 0.09–0.05 (s, 36H, TMS); ^13^C{^1^H} (101 MHz, [D_8_]THF, 297 K) δ (ppm) = 183.0/**182.9** (N*C*N), 68.3 (THF), 35.7/**35.2** (*C*H_2_), 26.4 (THF), 14.4/14.4 (*C*H_3_), 3.5/3.5 (TMS); ^29^Si{^1^H} DEPT‐NMR (79.5 MHz, [D_8_]THF, 297 K) δ (ppm) = −11.2/**−12.0/−13.0**; IR (ATR, *ν* [cm^−1^]) = 2952 (m), 2891 (w), 2197 (w), 2123 (w), 2091 (w), 1665 (w), 1464 (m), 1448 (m), 1398 (m), 1242 (m), 1072 (m), 1040 (m), 962 (w), 823 (s), 748 (s), 667 (m).

### [(thf)_3_Ca{MesC(N─iPr)_2_}I] (**2**)

Freshly rasped calcium (6.0 mmol, 1.2 equiv.) and diisopropylcarbodiimide (5.0 mmol, 1.0 equiv.) were suspended in THF (10 mL). Mesityl iodide (5.0 mmol, 1.0 equiv.) was added, and the reaction mixture was stirred at room temperature for 20 h (conversion: 87 %). The solution was separated from the colorless solid and *n*‐pentane (2 mL) was added. Colorless crystals were obtained by storage at room temperature overnight. The crystals were collected by filtration and dried carefully in vacuo (yield: 1.07 g, 1.70 mmol, 34%). ^1^H NMR (400 MHz, [D_8_]THF, 297 K) *δ* (ppm) = 6.78 (s, 2H, Mes), 3.64–3.59 (m, 8H, THF), 2.79 (sept, 2H, *i*Pr), 2.26–2.16 (m, 9H, CH_3_), 1.81–1.70 (m, 8H, THF), 0.93 (d, 12H, *i*Pr); ^13^C{^1^H} (101 MHz, [D_8_]THF, 297 K) δ (ppm) = **172.9**/172.8 (N*C*N), **136.1**/136.0 (*C*_ipso_), 134.8/**134.6** (*C*_Ar_), **128.4**/128.2 (*C*_Ar_), 68.3 (THF), 48.5/48.5 (*i*Pr, *C*H), 28.1/**27.8** (*i*Pr, *C*H_3_), 26.4 (THF), 21.4/**21.3** (Ar─CH_3_), 20.8 (Ar─CH_3_); IR (ATR, ν [cm^−1^]) = 2965 (m), 2874 (w), 1628 (m), 1459 (m), 1369 (w), 1331 (m), 1176 (w), 1162 (w), 1135 (w), 1031 (s), 1005 (w), 914 (w), 879 (m), 851 (m), 670 (w), 547 (w).

### [(thf)_2_Ca{Ad─C(N─C_6_H_3_‐2,6‐iPr_2_)(N─C_6_H_3_‐2‐(CPh_2_)‐6‐(CHPh_2_))}]∙(thf) (**3**)

Dimethylcalcium (1.2 mmol, 1.0 equiv.) was suspended in THF (5 mL) and a solution of 1‐(2,6‐diisopropylphenyl)‐2‐(1‐adamantyl)‐3‐[2,6‐bis(diphenyl‐methyl)‐4‐methylphenyl]amidine (1.2 mmol, 1.0 equiv.) in THF (15 mL) was added. The solution turned red immediately and was stirred for 1 h at room temperature. A few red crystals were obtained after several days during storage of the reaction mixture at −20 °C. (yield: 0.16 g, 0.16 mmol, 13 %). ^1^H NMR (400 MHz, [D_8_]THF, 297 K) *δ* (ppm) = 7.30–6.86 (m, 20H, ArH), 6.84 (s, 1H, TolH), 6.74 (t, 1H, ArH), 6.57 (s, 1H, TolH), 6.42 (s, 1H, ArH), 6.15 (s, 1H, CHPh_2_), 5.55 (t, 1H, ArH), 3.64–3.58 (m, 8H, THF), 3.56 (sept, 1H, *i*Pr), 3.08 (sept, 1H, *i*Pr), 2.18 (s, 3H, CH_3_), 1.80–1.73 (m, 8H, THF), 1.67–1.51 (dd, 6H, AdH), 1.28 (s, 3H, AdH), 1.23–1.01 (m, 6H, AdH), 0.99–0.86 (m, 12H, CH(CH_3_)_2_); ^13^C{^1^H} (101 MHz, [D_8_]THF, 297 K) *δ* (ppm) = 173.8, 148.5, 147.5, 146.5, 145.8, 142.5, 141.5, 136.8, 135.5, 130.9, 130.7, 129.0, 128.7, 128.6, 126.6, 126.5, 124.4, 123.3, 123.0, 121.9, 117.0, 105.7, 68.2, 52.8, 47.9, 39.4, 36.9, 32.5, 29.5, 29.0, 28.2, 26.4, 23.6, 23.5, 22.3, 21.2.; IR (ATR, ν [cm^−1^]) = 2959 (w), 2904 (m), 1637 (w), 1599 (w), 1492 (m), 1446 (m), 1427 (m), 1385 (w), 1260 (w), 1064 (m), 1031 (m), 911 (w), 802 (w), 761 (m), 748 (m), 700 (s), 605 (w).

## Author Contributions

S.S.: Conceptualization, synthesis and characterization, data analysis and methodology, data acquisition, writing original draft and editing; M.B.: synthesis and characterization, data acquisition; P.K.: X‐ray structure determinations at single crystals and editing of manuscript; M.W.: Conceptualization and supervision, writing original draft and editing. All authors have given approval to the final version of the manuscript.

## Conflict of Interest

The authors declare no conflict of interest.

## Supporting information



Supporting Information

## Data Availability

NMR spectra, experimental procedures, crystallographic and refinement details, presentation of the molecular structure of compound **1a** (pdf format). Deposition Numbers 2414721–2414724 contains the supplementary crystallographic data for this paper (see Table S1, Supporting Information). These data are provided free of charge by the joint Cambridge Crystallographic Data Centre and Fachinformationszentrum Karlsruhe http://www.ccdc.cam.ac.uk/structures.

## References

[1] a) M. P. Coles , Coord. Chem. Rev. 2015, 297–298, 2;

[2] a) M. Niemeyer , P. P. Power , Inorg. Chem. 1997, 36, 4688; 11670145 10.1021/ic970319t

[3] a) D. Stalke , M. Wedler , F. T. Edelmann , J. Organomet. Chem. 1992, 431, C1–C5;

[4] a) T. Chlupatý , A. Růžička , Coord. Chem. Rev. 2016, 314, 103;

[5] a) Y.‐C. Tsai , Coord. Chem. Rev. 2012, 256, 722;

[6] M. Wedler , F. Knösel , M. Noltemeyer , F. T. Edelmann , J. Organomet. Chem. 1990, 388, 21.

[7] S. P. Green , C. Jones , Science 2007, 318 1754; 17991827 10.1126/science.1150856

[8] L. A. Freeman , J. E. Walley , R. J. Gilliard , Nature Synth. 2022, 1, 439;

[9] B. Rösch , S. Harder , Chem. Commun. 2021, 57, 9354; 10.1039/d1cc04147a 34528959

[10] A. Stasch , C. Jones , Dalton Trans. 2011, 40, 5659; 21390353 10.1039/c0dt01831g

[11] C. Jones , Coord. Chem. Rev. 2010, 254, 1273;

[12] S. Krieck , L. Yu , M. Reiher , M. Westerhausen , Eur. J. Inorg. Chem. 2010, 2010, 197.

[13] a) W. Huadsai , L. Vendier , H. Görls , L. Magna , S. Bontemps , M. Westerhausen , Eur. J. Inorg. Chem. 2024, 27, e202400128;

[14] L. Yu , R. Qian , X. Deng , F. Wang , Q. Xu , Sci. Bull. 2018, 63, 1010. 10.1016/j.scib.2018.06.002 36658887

[15] S. B. Kim , C. Yang , T. Powers , L. M. Davis , X. Lou , R. G. Gordon , Angew. Chem., Int. Ed. 2016, 55, 10228. 10.1002/anie.201602406 PMC5094530 27351794

[16] R. Green , A. C. Walker , M. P. Blake , P. Mountford , Polyhedron 2016, 116, 64.

[17] M. Arrowsmith , M. R. Crimmin , M. S. Hill , S. L. Lomas , M. S. Heng , P. B. Hitchcock , G. Kociok‐Köhn , Dalton Trans. 2014, 43, 14249. 24434957 10.1039/c3dt53542h

[18] A. G. M. Barrett , M. R. Crimmin , M. S. Hill , P. B. Hitchcock , S. L. Lomas , M. F. Mahon , P. A. Procopiou , K. Suntharalingam , Organometallics 2008, 27, 6300.

[19] C. N. de Bruin‐Dickason , G. B. Deacon , C. Jones , P. C. Junk , M. Wiecko , Eur. J. Inorg. Chem. 2019, 7, 1030.

[20] a) A. G. M. Barrett , M. R. Crimmin , M. S. Hill , P. B. Hitchcock , S. L. Lomas , M. F. Mahon , P. A. Procopiou , Dalton Trans. 2010, 39, 7393; 20607197 10.1039/c0dt00089b

[21] a) S. Harder , J. Langer , Nat. Rev. Chem. 2023, 7, 843; 37935796 10.1038/s41570-023-00548-0

[22] a) M. Westerhausen , A. Koch , H. Görls , S. Krieck , Chem. – Eur. J. 2017, 23, 1456; 27976821 10.1002/chem.201603786

[23] M. Westerhausen , Z. Anorg. Allg. Chem. 2009, 635, 13.

[24] a) S. Krieck , H. Görls , L. Yu , M. Reiher , M. Westerhausen , J. Am. Chem. Soc. 2009, 131, 2977; 19193100 10.1021/ja808524y

[25] a) R. Fischer , M. Gärtner , H. Görls , M. Westerhausen , Angew. Chem., Int. Ed. 2006, 45, 609; 10.1002/anie.200503452 16365845

[26] A. Koch , Q. Dufrois , M. Wirgenings , H. Görls , S. Krieck , M. Etienne , G. Pohnert , M. Westerhausen , Chem. – Eur. J. 2018, 24, 16840. 30095189 10.1002/chem.201803518

[27] S. Yadav , V. S. V. S. N. Swamy , R. Gonnade , S. S. Sen , ChemistrySelect 2016, 1, 1066.

[28] M. He , M. T. Gamer , P. W. Roesky , Organometallics 2016, 35, 2638.

[29] M. Gärtner , H. Görls , M. Westerhausen , Organometallics 2007, 26, 1077.

[30] a) S. Sengupta , S. Krieck , M. Westerhausen , Dalton Trans. 2024, 53, 14961; 39140331 10.1039/d4dt02035a

[31] S. Sengupta , P. Schüler , P. Liebing , M. Westerhausen , Chem. – Eur. J. 2023, 29, e202300035. 36734181 10.1002/chem.202300035

[32] S. Sengupta , P. Schüler , H. Görls , P. Liebing , S. Krieck , M. Westerhausen , Chem. – Eur. J. 2022, 28, e202201359. 35686618 10.1002/chem.202201359 PMC9546396

[33] a) P. Schüler , S. Sengupta , A. Koch , H. Görls , S. Krieck , M. Westerhausen , Chem. – Eur. J. 2022, 28, e202201897; 35912418 10.1002/chem.202201897 PMC9804548

[34] B. M. Wolf , C. Stuhl , C. Maichle‐Mössmer , R. Anwander , J. Am. Chem. Soc. 2016, 140, 2373. 10.1021/jacs.7b12984 29328671

[35] P. Stanetty , M. D. Mihovilovic , J. Org. Chem. 1997, 62, 1514.

[36] M. Schlosser , Polare Organometalle: Struktur und Reaktivität polarer Organometalle, Springer, Berlin, Heidelberg 1973.

[37] a) M. A. Guino‐o , A. Torvisco , W. Teng , K. Ruhlandt‐Senge , Inorg. Chim. Acta 2012, 389, 122.

[38] P. Schüler , S. Krieck , H. Görls , P. Liebing , M. Westerhausen , Dalton Trans. 2022, 51, 8461. 35603695 10.1039/d2dt01121b

[39] B. Maitland , A. Stasch , C. Jones , Aust. J. Chem. 2022, 75, 543.

[40] C. N. de Bruin‐Dickason , A. J. Boutland , D. Dange , G. B. Deacon , C. Jones , Dalton Trans. 2018, 47, 9512. 29964281 10.1039/c8dt02138d

[41] J. K. Schuster , D. K. Roy , C. Lenczyk , J. Mies , H. Braunschweig , Inorg. Chem. 2019, 58, 2652. 30707568 10.1021/acs.inorgchem.8b03263

[42] J. A. R. Schmidt , J. Arnold , J. Chem. Soc., Dalton Trans. 2002, 2002, 2890.

[43] M. B. Smith , March's Advanced Organic Chemistry: Reactions, Mechanisms, and Structure, 7th ed., Wiley, Hoboken, NJ 2013.

[44] a) A. Causero , H. Elsen , J. Pahl , S. Harder , Angew. Chem., Int. Ed. 2017, 56, 6906; 10.1002/anie.201703037 28474850

[45] C. Glock , C. Loh , H. Görls , S. Krieck , M. Westerhausen , Eur. J. Inorg. Chem. 2013, 2013, 3261.

[46] B. Srinivas , C.‐C. Chang , C.‐H. Chen , M. Y. Chiang , I.‐T. Chen , Y. Wang , G.‐H. Lee , J. Chem. Soc., Dalton Trans. 1997, 1997, 957.

[47] A. W. Addison , T. N. Rao , J. Reedijk , J. van Rijn , G. C. Verschoor , J. Chem. Soc., Dalton Trans. 1984, 1984, 1349.

[48] F. Feil , S. Harder , Organometallics 2000, 19, 5010.

[49] a) N. Y. Radkova , T. A. Kovylina , A. S. Shavyrin , A. V. Cherkasov , G. K. Fukin , K. A. Lyssenko , A. A. Trifonov , New J. Chem. 2020, 44, 7811;

[50] J. Dyall , M. S. Hill , M. F. Mahon , L. The , A. S. S. Wilson , Dalton Trans. 2019, 48, 4248. 30847456 10.1039/c8dt05107k

[51] a) D. Schulze‐Sünnighausen , J. Becker , M. R. M. Koos , B. Luy , J. Magn. Reson. 2017, 281, 151; 28603039 10.1016/j.jmr.2017.05.012

[52] R. Neufeld , D. Stalke , Chem. Sci. 2015, 6, 3354. 29142693 10.1039/c5sc00670h PMC5656982

[53] G. M. Sheldrick , Acta Crystallogr. 2015, A71, 3.

[54] G. M. Sheldrick , Acta Crystallogr. 2015, C71, 3.

[55] O. V. Dolomanov , L. J. Bourhis , R. J. Gildea , J. A. K. Howard , H. Puschmann , J. Appl. Crystallogr. 2009, 42, 339. 10.1107/S0021889811041161 PMC3236671 22199401

[56] A. X. S. Bruker , APEX3 or Higher Version, and SADABS, Bruker AXS Inc., Madison, WI 2001.

