# Supplementary material for: Synthesis of Heteroleptic Amidinato Calcium Halides via the In‐Situ Grignard Addition Method (iGAM) Complementing the Metalation of Amidines
Source: Chemistry. 2025 Mar 30;31(25):e202500210. doi: 10.1002/chem.202500210 (PMC12057596; doi:10.1002/chem.202500210)
Supplement: Supplementary file 1 — Supporting Information [file CHEM-31-e202500210-s001.pdf]

Content

1. Experimental Section 2

2. Analytical Data 10

2.1 [$(thf)_2Ca\{EtC(N-SiMe_3)_2\}(\mu-Br)]_2$ (1a) 10

2.2 [$(thf)_2Ca\{EtC(N-SiMe_3)_2\}(\mu-I)]_2$ (1b) 14

2.3 [$(thf)_3Ca\{MesC(N\text{-}i Pr)_2\}I$] (2) 17

2.4 [$(thf)_2Ca\{AdC(N-C_6H_3\text{-}2,6\text{-}iPr2)(N-C_6H_3\text{-}2\text{-}(CPh_2)\text{-}6\text{-}(CHPh_2))\}\cdot(thf)$] (3) 21

2.5 iGAM Screening 24

2.6 Protolysed iGAM reactions 32

2.7 Single-crystal X-ray structural analyses..37

1. Experimental Section

General procedure for the in-situ Grignard Addition Method (iGAM) with carbodiimids

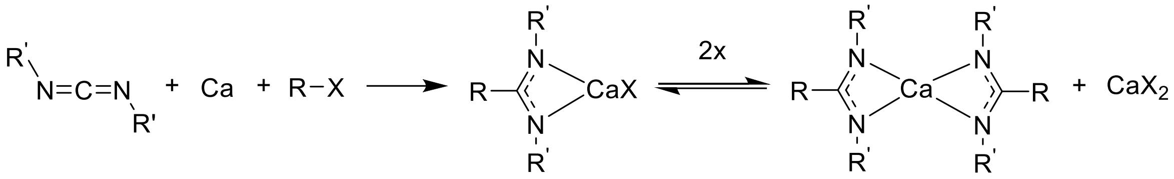

The reactions were carried out under an inert nitrogen atmosphere using standard Schlenk techniques. Freshly rasped calcium (5.8-7.5 mmol, 1.2-1.5 eq.), THF (10 mL) and the carbodiimid (4.8-5.0 mmol, 1.0 eq.) were placed in a Schlenk tube. Alkyl/aryl halide (4.8-5.0 mmol, 1.0 eq.) was added, and the reaction mixture was stirred at room temperature for 5-20 h. If the temperature of the reaction mixture increased, it was cooled down with ice. The conversion of the carbodiimid was determined by titration of a hydrolyzed aliquot with sulphuric acid (0.1 N) against phenolphthalein. Typical conversion rates are in a range of 80 to >95 %. Calcium amidinates were crystallized from concentrated solutions in THF or *n*-pentane.

[(thf)$_2$Ca{EtC(N-SiMe$_3$)$_2$}(μ-Br)]$_2$ (1a)

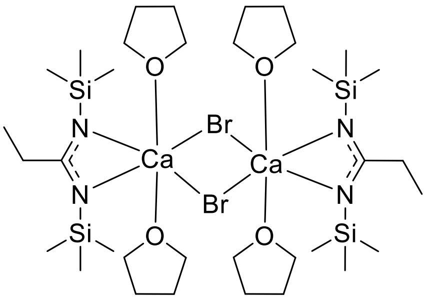

1a

Freshly rasped calcium (5.8 mmol, 1.2 eq.) and bis(trimethylsilyl)carbodiimide (4.8 mmol, 1.0 eq.) were suspended in THF (10 mL). Ethyl bromide (4.8 mmol, 1.0 eq.) was added, and the reaction mixture was stirred at room temperature for 6 h (conversion: >95 %). The solution was separated from the colorless solid and concentrated under reduced pressure. The residue was redissolved in *n*-pentane (10 mL) and stored at -20 °C overnight, yielding colorless crystals. The crystals were collected by filtration and dried carefully in vacuo (yield: 1.08 g, 1.13 mmol, 46 %).

^{1}H NMR (400 MHz, [D$_8$]THF, 297 K) δ (ppm) = 3.64-3.59 (m, 16H, THF), 2.27 (q, 4H, CH$_2$), 1.79-1.74 (m, 16H, THF), 1.13 (t, 6H, CH$_3$), 0.08-0.06 (s, 36H, TMS)

^{13}C{^{1}H} (101 MHz, [D$_8$]THF, 297 K) δ (ppm) = 183.0/**182.7** (N*C*N), 68.2 (THF), 35.7/**35.3** (*C*H$_2$), 26.4 (THF), 14.4/14.4 (*C*H$_3$), 3.5/**3.3** (TMS)

^{29}Si{^{1}H} DEPT-NMR (79.5 MHz, [D$_8$]THF, 297 K) δ (ppm) = -11.2/**-12.5/-13.0**

IR (ATR, ν [cm^{-1}]) = 3250 (w), 2953 (m), 2881 (m), 1682 (w), 1645 (w), 1605 (w), 1468 (m), 1446 (m), 1405 (w), 1253 (m), 1240 (m), 1193 (w), 1071 (m), 1040 (m), 918 (w), 825 (s), 748 (m), 666 (m).

[(thf)$_2$Ca{EtC(N-SiMe$_3$)$_2$}(μ-I)]$_2$ (1b)

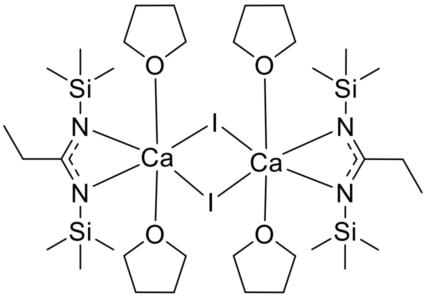

1b

Freshly rasped calcium (7.5 mmol, 1.5 eq.) and bis(trimethylsilyl)carbodiimide (5.0 mmol, 1.0 eq.) were suspended in THF (10 mL). Ethyl iodide (5.0 mmol, 1.0 eq.) was added, and the reaction mixture was stirred at room temperature for 5 h (conversion: >95 %). The solution was separated from the colorless solid and concentrated under reduced pressure. The residue was redissolved in *n*-pentane (12 mL) and stored at -20 °C overnight, yielding colorless crystals. The crystals were collected by filtration and dried carefully in vacuo (yield: 0.46 g, 0.44 mmol, 17 %).

^{1}H NMR (400 MHz, [D$_8$]THF, 297 K) δ (ppm) = 3.64-3.59 (m, 16H, THF), 2.27 (q, 4H, CH$_2$), 1.80-1.74 (m, 16H, THF), 1.12 (t, 6H, CH$_3$), 0.09-0.05 (s, 36H, TMS)

^{13}C{^{1}H} (101 MHz, [D$_8$]THF, 297 K) δ (ppm) = 183.0/**182.9** (N*C*N), 68.3 (THF), 35.7/**35.2** (*C*H$_2$), 26.4 (THF), 14.4/14.4 (*C*H$_3$), 3.5/3.5 (TMS)

^{29}Si{^{1}H} DEPT-NMR (79.5 MHz, [D$_8$]THF, 297 K) δ (ppm) = -11.2/**-12.0/-13.0**

IR (ATR, ν [cm^{-1}]) = 2952 (m), 2891 (w), 2197 (w), 2123 (w), 2091 (w), 1665 (w), 1464 (m), 1448 (m), 1398 (m), 1242 (m), 1072 (m), 1040 (m), 962 (w), 823 (s), 748 (s), 667 (m).

[(thf)$_3$Ca{MesC(N-iPr)$_2$}I] (2)

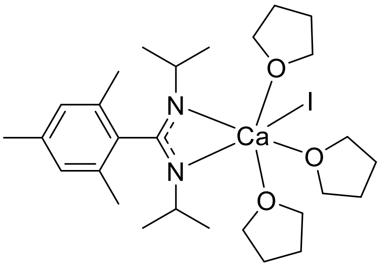

2

Freshly rasped calcium (6.0 mmol, 1.2 eq.) and diisopropylcarbodiimide (5.0 mmol, 1.0 eq.) were suspended in THF (10 mL). Mesityl iodide (5.0 mmol, 1.0 eq.) was added, and the reaction mixture was stirred at room temperature for 20 h (conversion: 87 %). The solution was separated from the colorless solid and *n*-pentane (2 mL) was added. Colorless crystals were obtained by storage at room temperature overnight. The crystals were collected by filtration and dried carefully in vacuo (yield: 1.07 g, 1.70 mmol, 34 %).

^{1}H NMR (400 MHz, [D$_8$]THF, 297 K) δ (ppm) = 6.78 (s, 2H, Mes), 3.64-3.59 (m, 8H, THF), 2.79 (sept, 2H, iPr), 2.26-2.16 (m, 9H, CH$_3$), 1.81-1.70 (m, 8H, THF), 0.93 (d, 12H, iPr)

$^{13}C\{^{1}H\}$ (101 MHz, [D$_8$]THF, 297 K) δ (ppm) = **172.9**/172.8 (N*C*N), **136.1**/136.0 (*C*$_{ipso}$), 134.8/**134.6** (*C*$_{Ar}$), **128.4**/128.2 (*C*$_{Ar}$), 68.3 (THF), 48.5/48.5 (^{i}Pr, *C*H), 28.1/**27.8** (^{i}Pr, *C*H$_3$), 26.4 (THF), 21.4/**21.3** (Ar-*C*H$_3$), 20.8 (Ar-*C*H$_3$)

IR (ATR, ν [cm^{-1}]) = 2965 (m), 2874 (w), 1628 (m), 1459 (m), 1369 (w), 1331 (m), 1176 (w), 1162 (w), 1135 (w), 1031 (s), 1005 (w), 914 (w), 879 (m), 851 (m), 670 (w), 547 (w).

[(thf)$_2$Ca{Ad-C(N-C$_6$H$_3$-2,6-iPr$_2$)(N-C$_6$H$_3$-2-(CPh$_2$)-6-(CHPh$_2$))}]·(thf) (3)

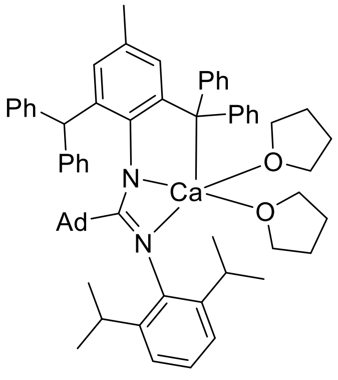

3

Dimethylcalcium (1.2 mmol, 1.0 eq.) was suspended in THF (5 mL) and a solution of 1-(2,6-diisopropylphenyl)-2-(1-adamantyl)-3-[2,6-bis(diphenylmethyl)-4-methylphenyl]amidine (1.2 mmol, 1.0 eq.) in THF (15 mL) was added. The solution turned red immediately and was stirred for 1 hour at room temperature. A few red crystals were obtained after several days during storage of the reaction mixture at -20 °C. (yield: 0.16 g, 0.16 mmol, 13 %).

^{1}H NMR (400 MHz, [D$_8$]THF, 297 K) δ (ppm) = 7.30-6.86 (m, 20H, ArH), 6.84 (s, 1H, TolH), 6.74 (t, 1H, ArH), 6.57 (s, 1H, TolH), 6.42 (s, 1H, ArH), 6.15 (s, 1H, CHPh$_2$), 5.55 (t, 1H, ArH), 3.64-3.58 (m, 8H, THF), 3.56 (sept, 1H, ^{i}Pr), 3.08 (sept, 1H, ^{i}Pr), 2.18 (s, 3H, CH$_3$), 1.80-1.73 (m, 8H, THF), 1.67-1.51 (dd, 6H, AdH), 1.28 (s, 3H, AdH), 1.23-1.01 (m, 6H, AdH), 0.99-0.86 (m, 12H, CH(CH$_3$)$_2$)

$^{13}C\{^{1}H\}$ (101 MHz, [D$_8$]THF, 297 K) δ (ppm) = 173.8, 148.5, 147.5, 146.5, 145.8, 142.5, 141.5, 136.8, 135.5, 130.9, 130.7, 129.0, 128.7, 128.6, 126.6, 126.5, 124.4, 123.3, 123.0, 121.9, 117.0, 105.7, 68.2, 52.8, 47.9, 39.4, 36.9, 32.5, 29.5, 29.0, 28.2, 26.4, 23.6, 23.5, 22.3, 21.2.

IR (ATR, ν [cm^{-1}]) = 2959 (w), 2904 (m), 1637 (w), 1599 (w), 1492 (m), 1446 (m), 1427 (m), 1385 (w), 1260 (w), 1064 (m), 1031 (m), 911 (w), 802 (w), 761 (m), 748 (m), 700 (s), 605 (w).

iGAM Screening reactions:

[(thf)$_x$Ca{MeC(N-SiMe$_3$)$_2$}(I)]$_n$

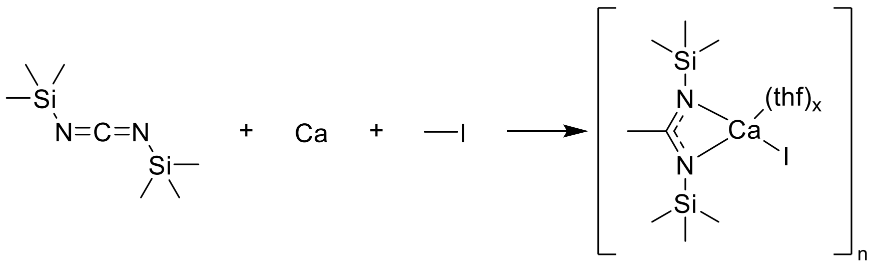

Freshly rasped calcium (5.8 mmol, 1.2 eq.) and bis(trimethylsilyl)carbodiimide (4.8 mmol, 1.0 eq.) were suspended in THF (10 mL). Methyl iodide (4.8 mmol, 1.0 eq.) was added, and the reaction mixture was stirred at room temperature for 20 h (conversion: 40 %).

[(thf)$_x$Ca{^{i}PrC(N-SiMe$_3$)$_2$}(I)]$_n$

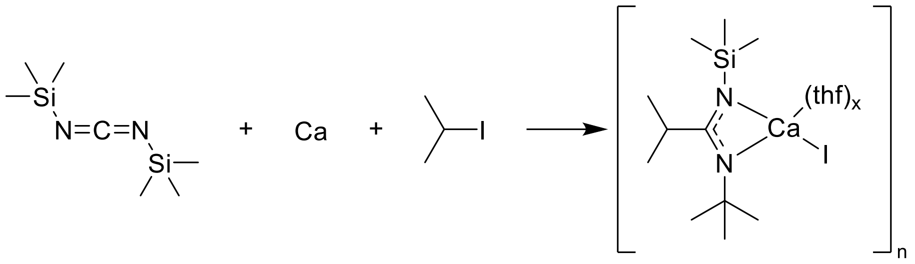

Freshly rasped calcium (6.0 mmol, 1.2 eq.) and bis(trimethylsilyl)carbodiimide (5.0 mmol, 1.0 eq.) were suspended in THF (10 mL). Isopropyl iodide (5.0 mmol, 1.0 eq.) was added, and the reaction mixture was stirred at room temperature for 20 h (conversion: 2 %).

[(thf)$_x$Ca{PhC(N-SiMe$_3$)$_2$}(I)]$_n$

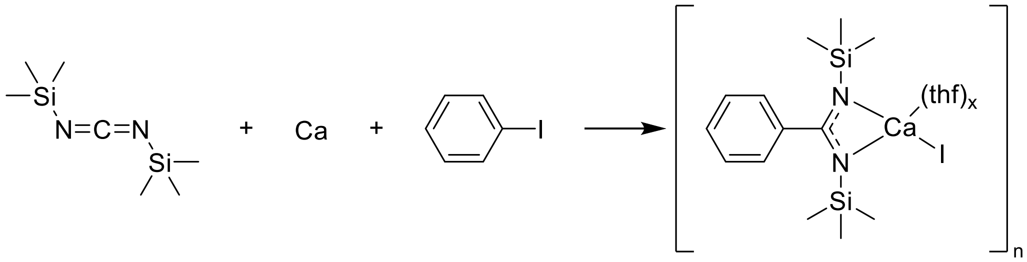

Freshly rasped calcium (5.8 mmol, 1.2 eq.) and bis(trimethylsilyl)carbodiimide (4.8 mmol, 1.0 eq.) were suspended in THF (10 mL). Phenyl iodide (4.8 mmol, 1.0 eq.) was added, and the reaction mixture was stirred at room temperature for 20 h (conversion: 84 %).

[(thf)$_x$Ca{MeC(N-^{i}Pr)$_2$}(I)]$_n$

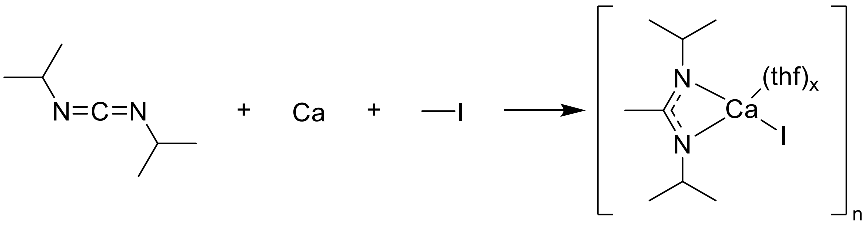

Freshly rasped calcium (6.0 mmol, 1.2 eq.) and diisopropylcarbodiimide (5.0 mmol, 1.0 eq.) were suspended in THF (10 mL). Methyl iodide (5.0 mmol, 1.0 eq.) was added, and the reaction mixture was stirred at room temperature for 20 h (conversion: 91 %, NMR-Yield: 90 %).

^{1}H NMR (400 MHz, [D$_8$]THF, 297 K) δ (ppm) = 3.69-3.59 (m, THF), 3.42 (sept, 2H, iPr), 1.81-1.72 (m, THF + 3H, CH$_3$), 0.99 (d, 12H, iPr)

^{13}C{^{1}H} (101 MHz, [D$_8$]THF, 297 K) δ (ppm) = **170.0**/168.6 (N*C*N), 68.5 (THF), 47.8/**47.7** (^{i}Pr*,* *C*H), 27.6/**27.4** (^{i}Pr*,* *C*H$_3$), 26.4 (THF), 12.3/**10.7** (*C*H$_3$), NMR-Yield: 90 %.

[(thf)$_x$Ca{EtC(N-^{i}Pr)$_2$}(Br)]$_n$

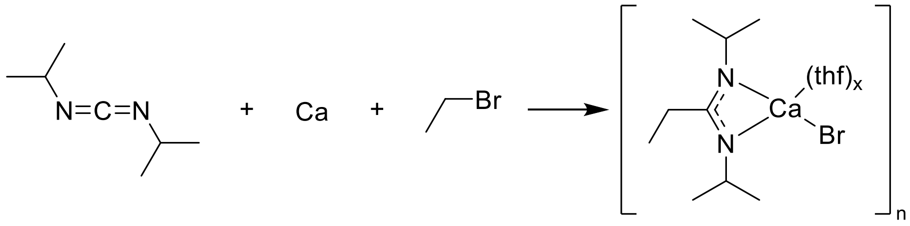

Freshly rasped calcium (6.0 mmol, 1.2 eq.) and diisopropylcarbodiimide (5.0 mmol, 1.0 eq.) were suspended in THF (10 mL). Ethyl bromide (5.0 mmol, 1.0 eq.) was added, and the reaction mixture was stirred at room temperature for 20 h (conversion: >95 %, NMR-Yield: 70 %).

^{1}H NMR (400 MHz, [D$_8$]THF, 297 K) δ (ppm) = 3.67-3.60 (m, THF), 3.45 (sept, 2H, ^{i}Pr), 2.23 (q, CH$_2$), 1.79-1.74 (m, THF), 1.06-0.96 (m, 12H, ^{i}Pr + 3H, CH$_3$)

^{13}C{^{1}H} (101 MHz, [D$_8$]THF, 297 K) δ (ppm) = **174.0**/172.8 (N*C*N), 68.4 (THF), 47.2/**47.1** (^{i}Pr, *C*H), 28.1/**27.8** (^{i}Pr, *C*H$_3$), 26.4 (THF), 18.3 (CH$_2$), 13.7/**13.6** (*C*H$_3$)

[(thf)$_x$Ca{EtC(N-^{i}Pr)$_2$}(I)]$_n$

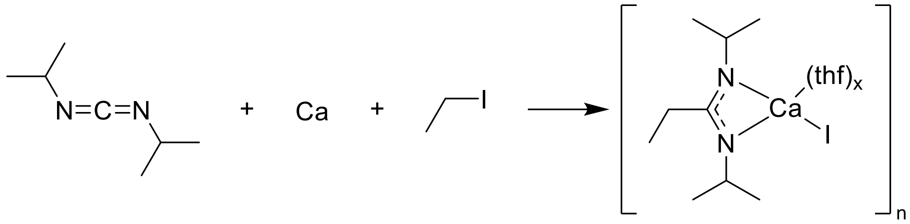

Freshly rasped calcium (6.0 mmol, 1.2 eq.) and diisopropylcarbodiimide (5.0 mmol, 1.0 eq.) were suspended in THF (10 mL). Ethyl iodide (5.0 mmol, 1.0 eq.) was added, and the reaction mixture was stirred at room temperature for 20 h (conversion: 80 %, NMR-Yield: 74 %).

^{1}H NMR (400 MHz, [D$_8$]THF, 297 K) δ (ppm) = 3.68-3.59 (m, THF), 3.50 (sept, 2H, ^{i}Pr), 2.28 (q, CH$_2$), 1.79-1.74 (m, THF), 1.10-0.99 (m, 12H, ^{i}Pr + 3H, CH$_3$)

^{13}C{^{1}H} (101 MHz, [D$_8$]THF, 297 K) δ (ppm) = **174.2**/172.8 (6 (N*C*N), 68.4 (THF), 47.2/**47.1** (^{i}Pr, *C*H), 28.1/**27.8** (^{i}Pr, *C*H$_3$), 26.4 (THF), 18.2 (CH$_2$), **13.5**/13.2 (*C*H$_3$)

[(thf)$_x$Ca{^{i}PrC(N-^{i}Pr)$_2$}(I)]$_n$

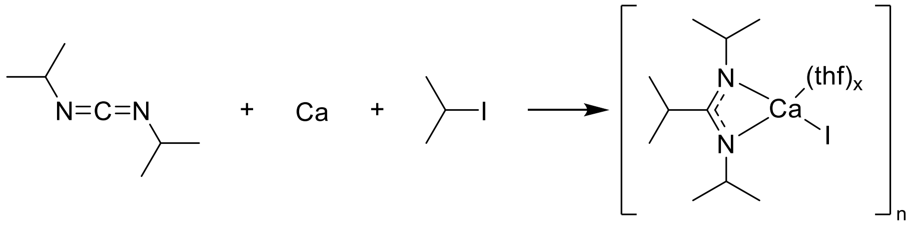

Freshly rasped calcium (6.0 mmol, 1.2 eq.) and diisopropylcarbodiimide (5.0 mmol, 1.0 eq.) were suspended in THF (10 mL). Isopropyl iodide (5.0 mmol, 1.0 eq.) was added, and the reaction mixture was stirred at room temperature for 20 h (conversion: 13 %).

[(thf)$_x$Ca{^{t}BuC(N-^{i}Pr)$_2$}(Br)]$_n$

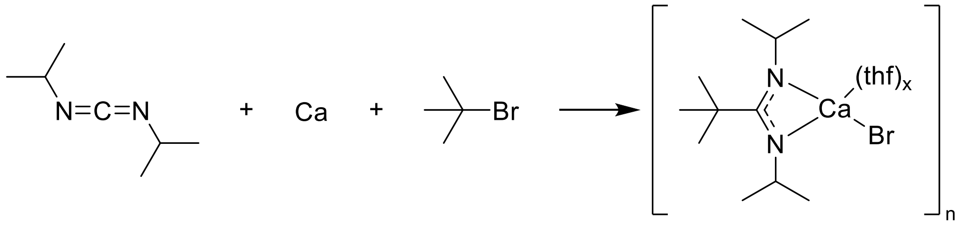

Freshly rasped calcium (6.0 mmol, 1.2 eq.) and diisopropylcarbodiimide (5.0 mmol, 1.0 eq.) were suspended in THF (10 mL). Tertbutyl bromide (5.0 mmol, 1.0 eq.) was added, and the reaction mixture was stirred at room temperature for 20 h (conversion: 5 %).

[(thf)$_x$Ca{AdC(N-^{i}Pr)$_2$}(Br)]$_n$

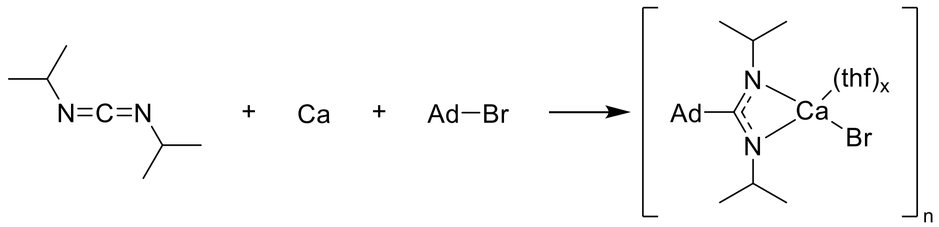

Freshly rasped calcium (6.0 mmol, 1.2 eq.) and diisopropylcarbodiimide (5.0 mmol, 1.0 eq.) were suspended in THF (10 mL). Adamantyl bromide (5.0 mmol, 1.0 eq.) was added, and the reaction mixture was stirred at room temperature for 20 h (conversion: 5 %).

[(thf)$_x$Ca{PhC(N-^{i}Pr)$_2$}(I)]$_n$

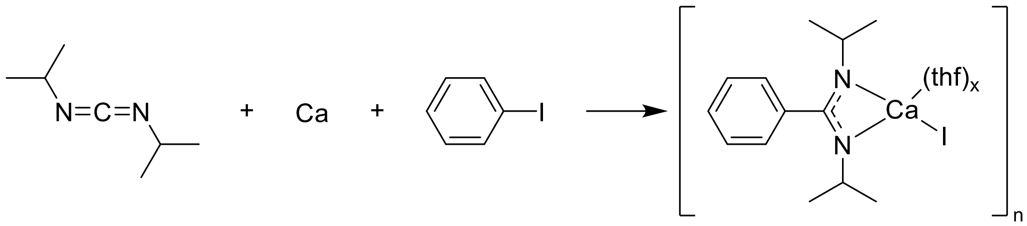

Freshly rasped calcium (6.0 mmol, 1.2 eq.) and diisopropylcarbodiimide (5.0 mmol, 1.0 eq.) were suspended in THF (10 mL). Phenyl bromide (5.0 mmol, 1.0 eq.) was added, and the reaction mixture was stirred at room temperature for 20 h (conversion: >95 %, NMR-Yield: 85 %).

^{1}H NMR (400 MHz, [D$_8$]THF, 297 K) δ (ppm) = 7.30 (t, 3 H, ArH), 6.98 (d, 2H, ArH), 3.67-3.62 (m, THF), 2.90 (sept, 2H, ^{i}Pr), 1.79-1.74 (m, THF), 0.90 (d, 12H, ^{i}Pr)

^{13}C{^{1}H} (101 MHz, [D$_8$]THF, 297 K) δ (ppm) = **174.1**/173.2 (N*C*N), 140.3/**139.4** (*C*$_{ipso}$), **128.6**/128.4 (Ar*C*), **127.3**/127.2 (Ar*C*), 68.4 (THF), 49.4/**48.1** (^{i}Pr*,* *C*H), 27.8/**27.4** (^{i}Pr*,* *C*H$_3$), 26.4 (THF)

Additionproducts after protolysis, general procedure: The reaction mixture of the iGAM was quenched with H_2O. The organic layer was separated, washed with NaOH (10 %) and extracted with DCM. The solvents were removed under reduced pressure and the oily residue was analysed using NMR spectroscopy.

***N,N'*-Bis(diisopropyl)acetimidamide**

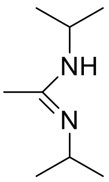

^{1}H-NMR (300 MHz, $CDCl_3$, 297 K) δ (ppm) = 3.62 (sept, 2H, ^{i}Pr), 1.82 (s, 3H, CH_3), 1.10 (d, 12H, ^{i}Pr)
^{13}C-NMR (75.5 MHz, $CDCl_3$, 297 K) δ (ppm) = 154.4 (N*C*N), 45.4 (^{i}Pr, *C*H), 23.8 (^{i}Pr, *C*H_3), 19.9 (*C*H_3)

***N,N'*-Bis(diisopropyl)propionimidamide**

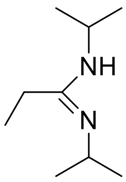

^{1}H-NMR (300 MHz, $CDCl_3$, 297 K) δ (ppm) = 3.66 (sept, 3H, ^{i}Pr), 2.18 (q, 2H, CH_2), 1.10 (d, 12H, ^{i}Pr + t, 3H, CH_3)
^{13}C-NMR (75.5 MHz, $CDCl_3$, 297 K) δ (ppm) = 158.4 (N*C*N), 45.0 (^{i}Pr, *C*H), 24.0 (^{i}Pr, *C*H_3), 22.9 (*C*H_2), 12.5 (*C*H_3)

***N,N'*-Bis(diisopropyl)benzimidamide**

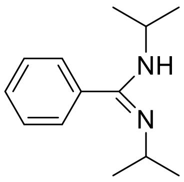

^{1}H-NMR (300 MHz, $CDCl_3$, 297 K) δ (ppm) = 7.41-7.33 (m, 3H, ArH), 7.25-7.18 (m, 2H, ArH), 3.56 (s, br, 2H, ^{i}Pr), 1.08 (d, 12H, ^{i}Pr)
^{13}C-NMR (75.5 MHz, $CDCl_3$, 297 K) δ (ppm) = 156.7 (N*C*N), 136.8 (C_{ipso}), 128.6 (Ar*C*), 128.5 (Ar*C*), 127.5 (Ar*C*) 46.4 (^{i}Pr, *C*H), 24.2 (^{i}Pr, *C*H_3)

***N,N'*-Bis(diisopropyl)-2,4,6-trimethylbenzimidamide**

Protolysis of [(thf)$_3$Ca{MesC(N-iPr)$_2$}I] **(2):**

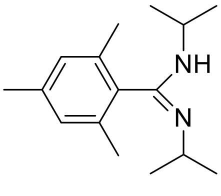

^{1}H-NMR (300 MHz, $CDCl_3$, 297 K) δ (ppm) = 7.00 (s, 2H, ArH), 3.12 (sept, 2H, ^{i}Pr), 2.33 (s, 3H, Ar-CH_3), 2.26 (s, 6H, Ar-CH_3), 1.20 (d, 12H, ^{i}Pr)
^{13}C-NMR (75.5 MHz, $CDCl_3$, 297 K) δ (ppm) = 163.9 (N*C*N), 141.7 (C_{ipso}), 134.8 (Ar*C*), 129.5 (Ar*C*), 122.7 (Ar*C*), 47.7 (^{i}Pr, *C*H), 23.3 (^{i}Pr, CH_3), 21.3 (Ar-CH_3), 19.4 (Ar-CH_3)

2. Analytical Data

2.1 [(thf)$_2$Ca{EtC(N-SiMe$_3$)$_2$}(μ-Br)]$_2$ (1a)

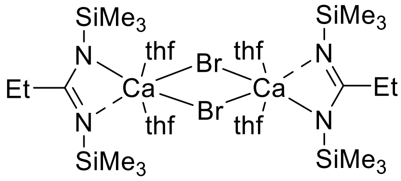

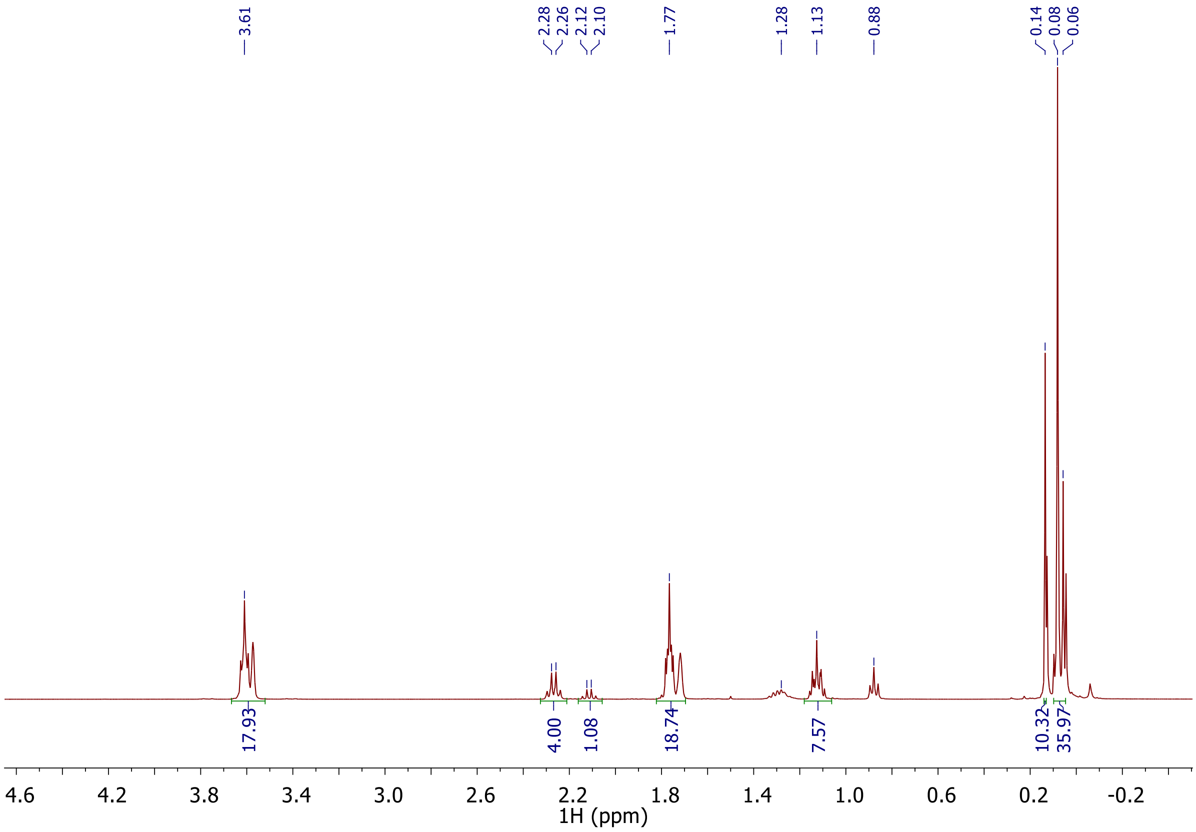

Figure S1: ^{1}H NMR spectrum (400 MHz, THF-d$_8$, 297K) of [(thf)$_2$Ca{EtC(N-SiMe$_3$)$_2$}(μ-Br)]$_2$ (1a). Hydrolysis product: 2.11, 1.13 (overlaps with 1a), 0.14 ppm. Solvents: THF (3.61, 1.77 ppm), n-pentane (1.28, 0.88 ppm).

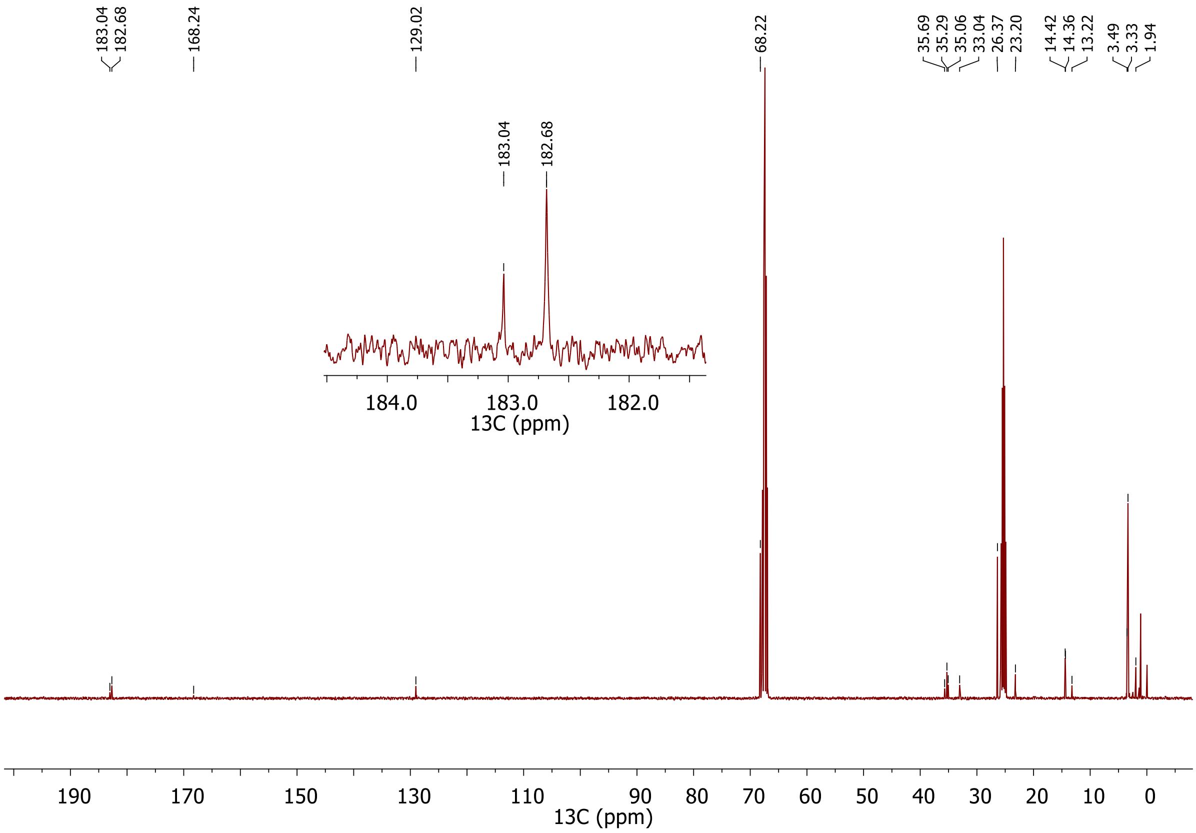

Figure S2: $^{13}C\{^{1}H\}$ NMR spectrum (101 MHz, THF-d_8, 297K) of [(thf)$_2$Ca{EtC(N-SiMe$_3$)$_2$}(μ-Br)]$_2$ (1a). Hydrolysis product: 168.2, 33.0, 13.2, 1.94 ppm. Solvents: THF (68.2, 26.4 ppm), n-pentane (35.0, 23.2, 14.4 ppm). 129.0 ppm: benzene (Impurity of the solvent)

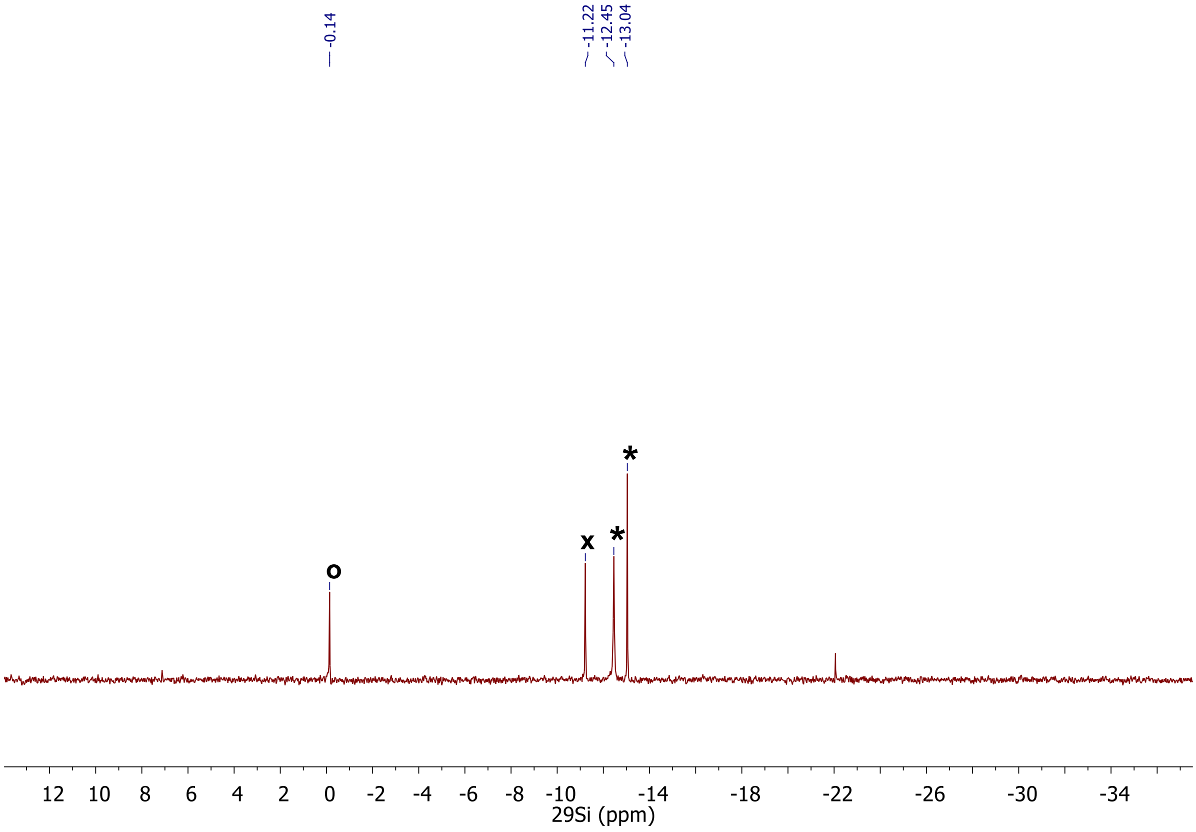

Figure S3: $^{29}Si\{^{1}H\}$-DEPT NMR spectrum (79.5 MHz, THF-d_8, 297K) of [(thf)$_2$Ca{EtC(N-SiMe$_3$)$_2$}(μ-Br)]$_2$ (1a) (*). (o): Hydrolysis (-0.14 ppm), (x): homoleptic species (-11.2 ppm), -22 ppm: grease.

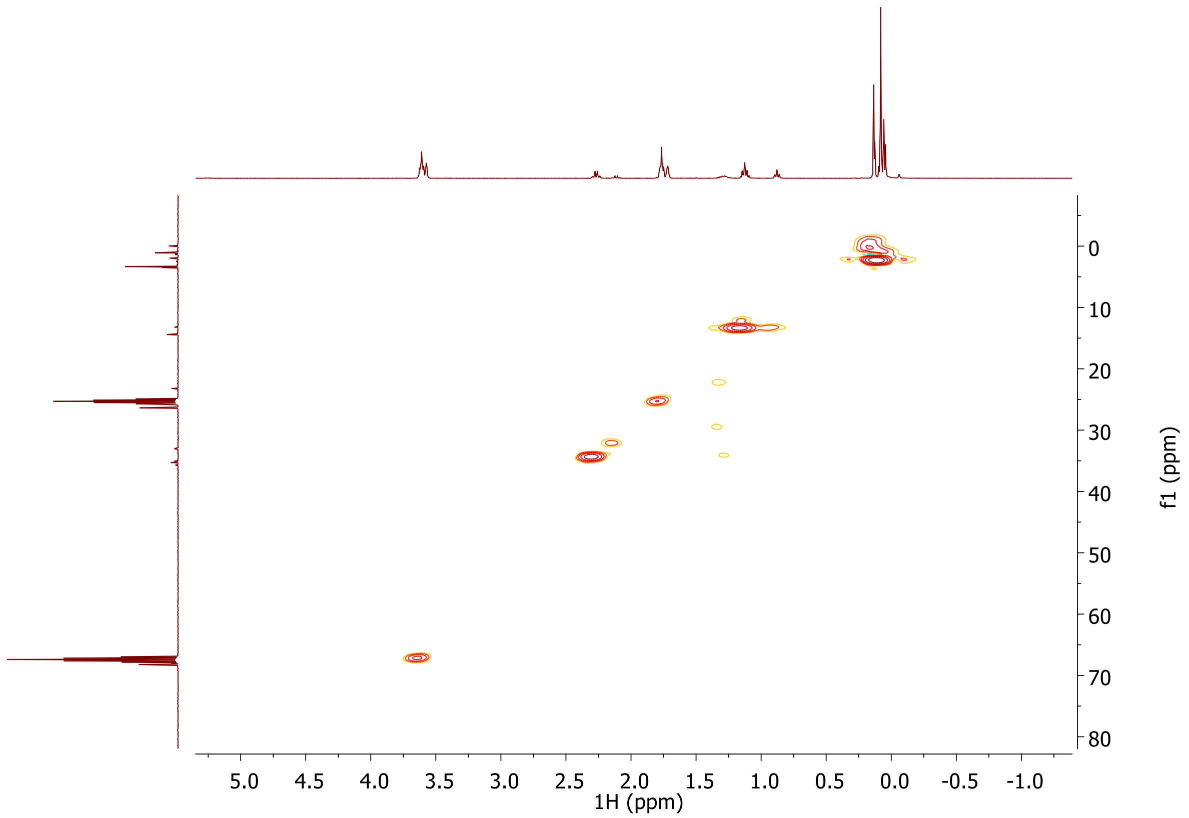

Figure S4: ASAP-HSQC-DEPT NMR spectrum (400 MHz, THF-d$_8$, 297K) of [(thf)$_2$Ca{EtC(N-SiMe$_3$)$_2$}(μ-Br)]$_2$ (1a).

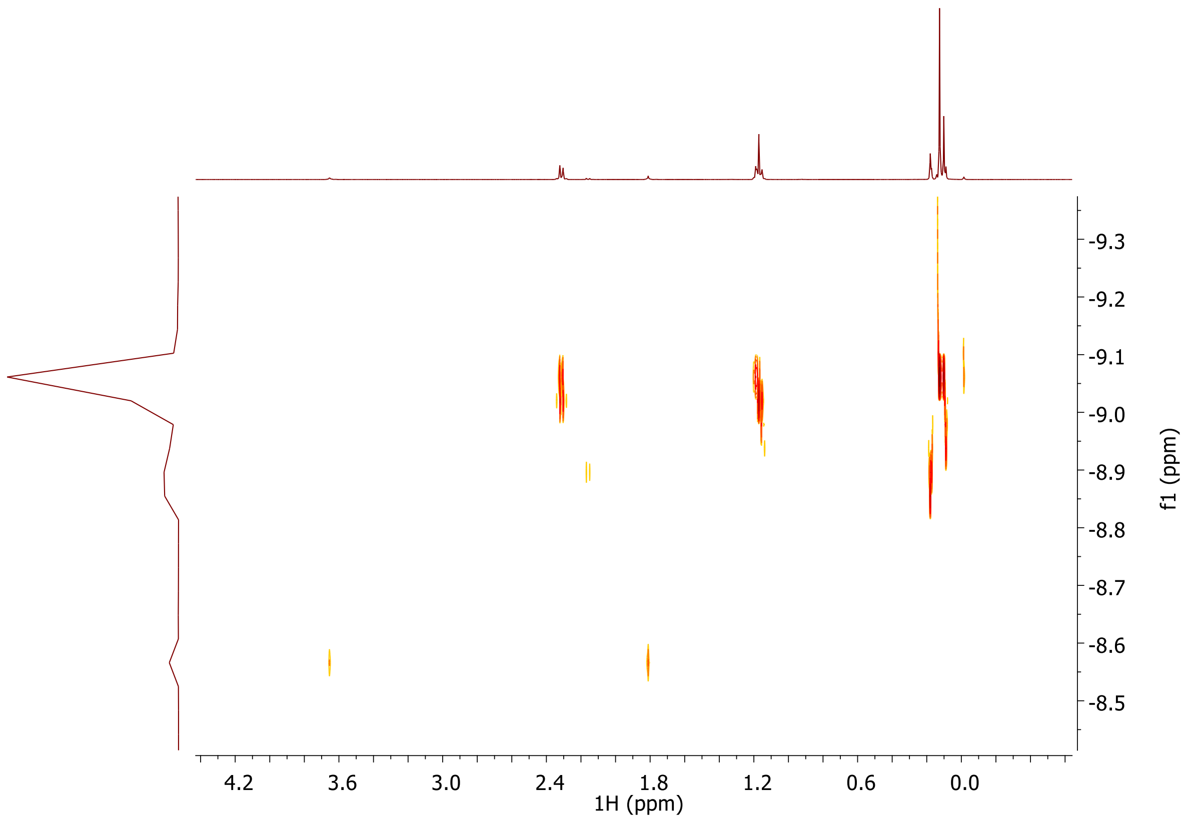

Figure S5: ^{1}H-DOSY NMR spectrum (400 MHz, THF-d$_8$, 297K) of [(thf)$_2$Ca{EtC(N-SiMe$_3$)$_2$}(μ-Br)]$_2$ (1a).

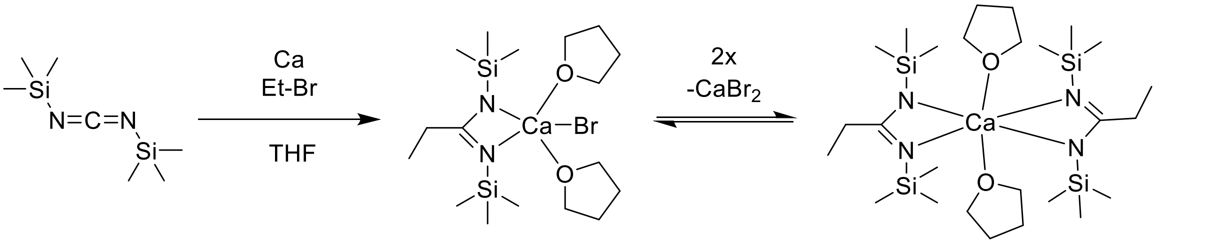

^{1}H δ(ppm) = 0.08
D(Ref) = 2.79x10^{-9} m^{2}/s (THF-d$_{7}$)
D(Subst) = 9.67x10^{-10} m^{2}/s
MW(calc) = 467 g/mol
MW(exp) = 479.6 g/mol
ΔMW = 3 %

^{1}H δ(ppm) = 0.06-0.04
D(Ref) = 2.79x10^{-9} m^{2}/s (THF-d$_{7}$)
D(Subst) = 8.86x10^{-10} m^{2}/s
MW(calc) = 543 g/mol
MW(exp) = 543.1 g/mol
ΔMW = 0 %

Scheme S1: Calculation of the molar masses using ECC-DOSY method (dissipated spheres and ellipsoids (DSE) calibration curve).

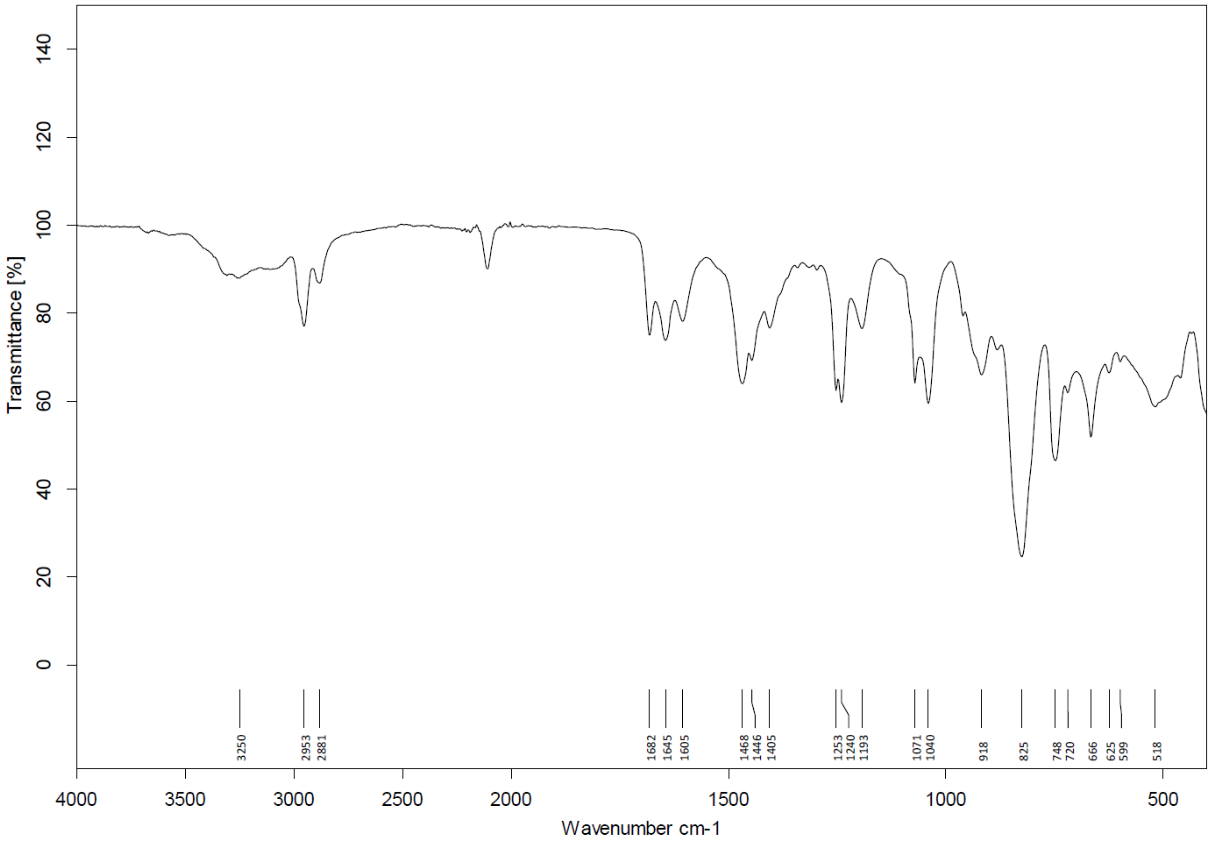

Figure S6: IR spectrum (ATR, ν [cm^{-1}]) of [(thf)$_2$Ca{EtC(N-SiMe$_3$)$_2$}(μ-Br)]$_2$ (1a).

2.2 [(thf)$_2$Ca{EtC(N-SiMe$_3$)$_2$}(μ-I)]$_2$ (1b)

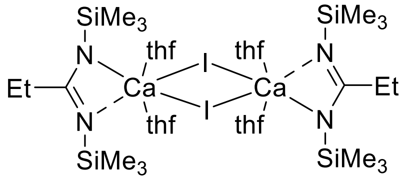

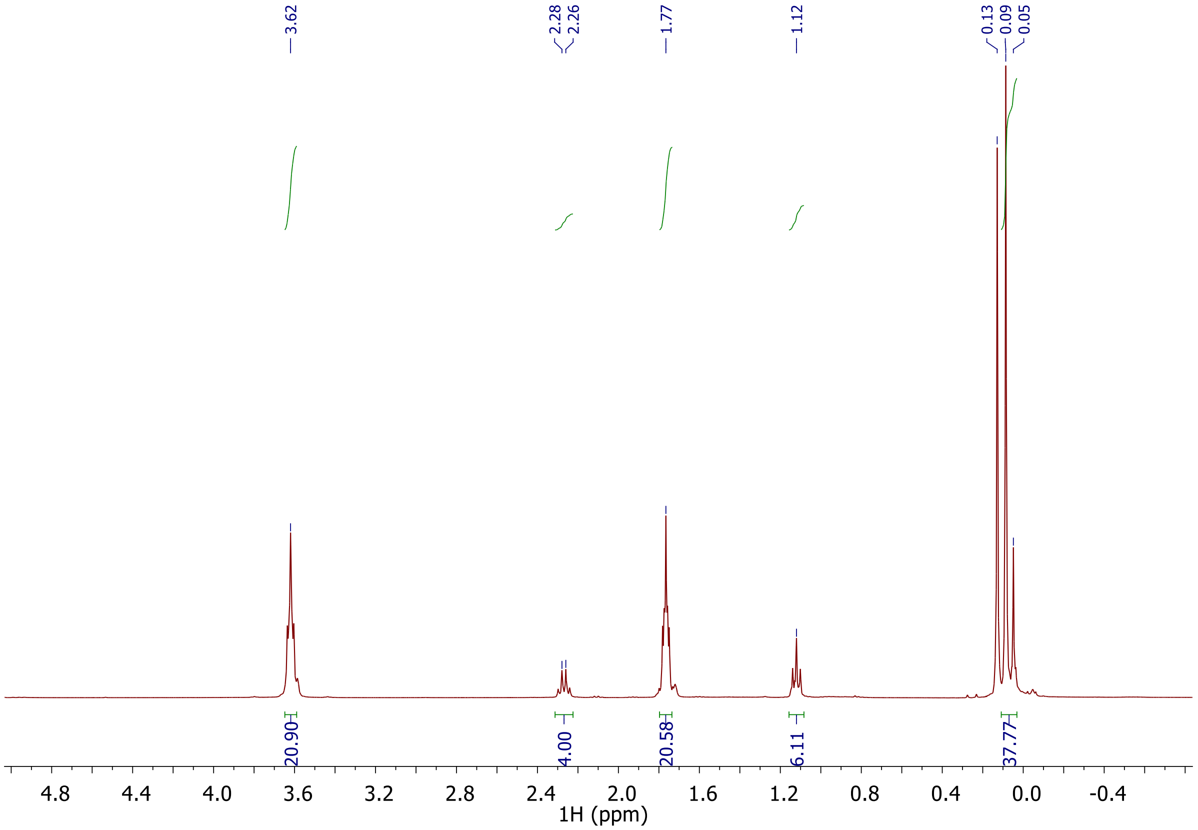

Figure S7: ^{1}H NMR spectrum (400 MHz, THF-d$_8$, 297K) of [(thf)$_2$Ca{EtC(N-SiMe$_3$)$_2$}(μ-I)]$_2$ (1b).

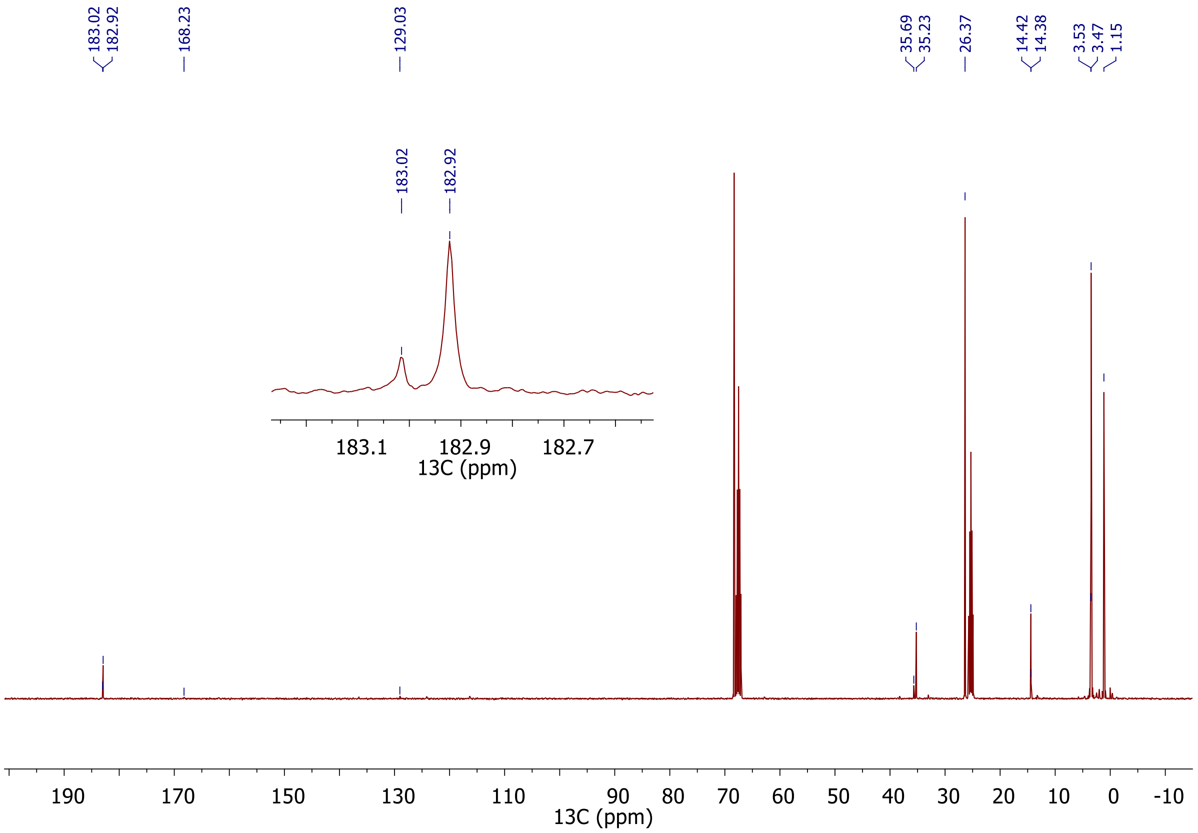

Figure S8: $^{13}C\{^1H\}$ NMR spectrum (101 MHz, THF-d_8, 297K) of [(thf)$_2$Ca{EtC(N-SiMe$_3$)$_2$}(µ-I)]$_2$ (1b). Hydrolysis product: 168.2 ppm. 129.0 ppm: benzene (Impurity of the solvent).

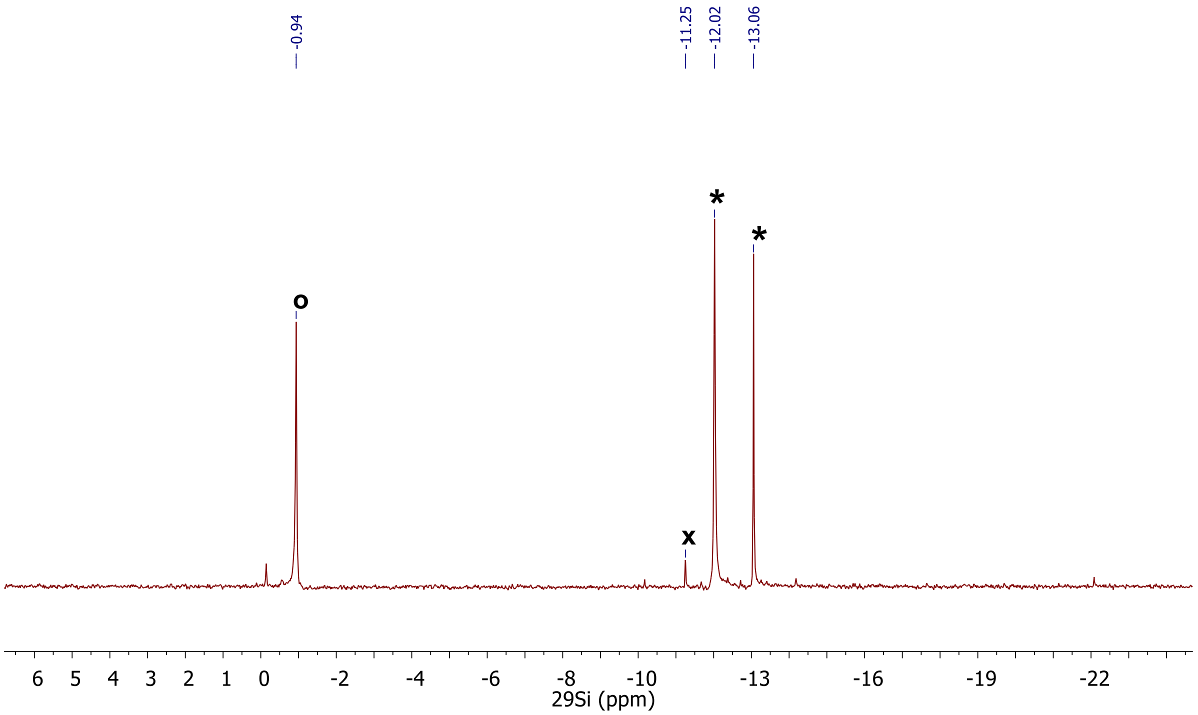

Figure S9: $^{29}Si\{^1H\}$-DEPT NMR spectrum (79.5 MHz, THF-d_8, 297K) of [(thf)$_2$Ca{EtC(N-SiMe$_3$)$_2$}(µ-I)]$_2$ (1b) (*). (o): Hydrolysis (-0.94 ppm), (x): homoleptic species (-11.2 ppm).

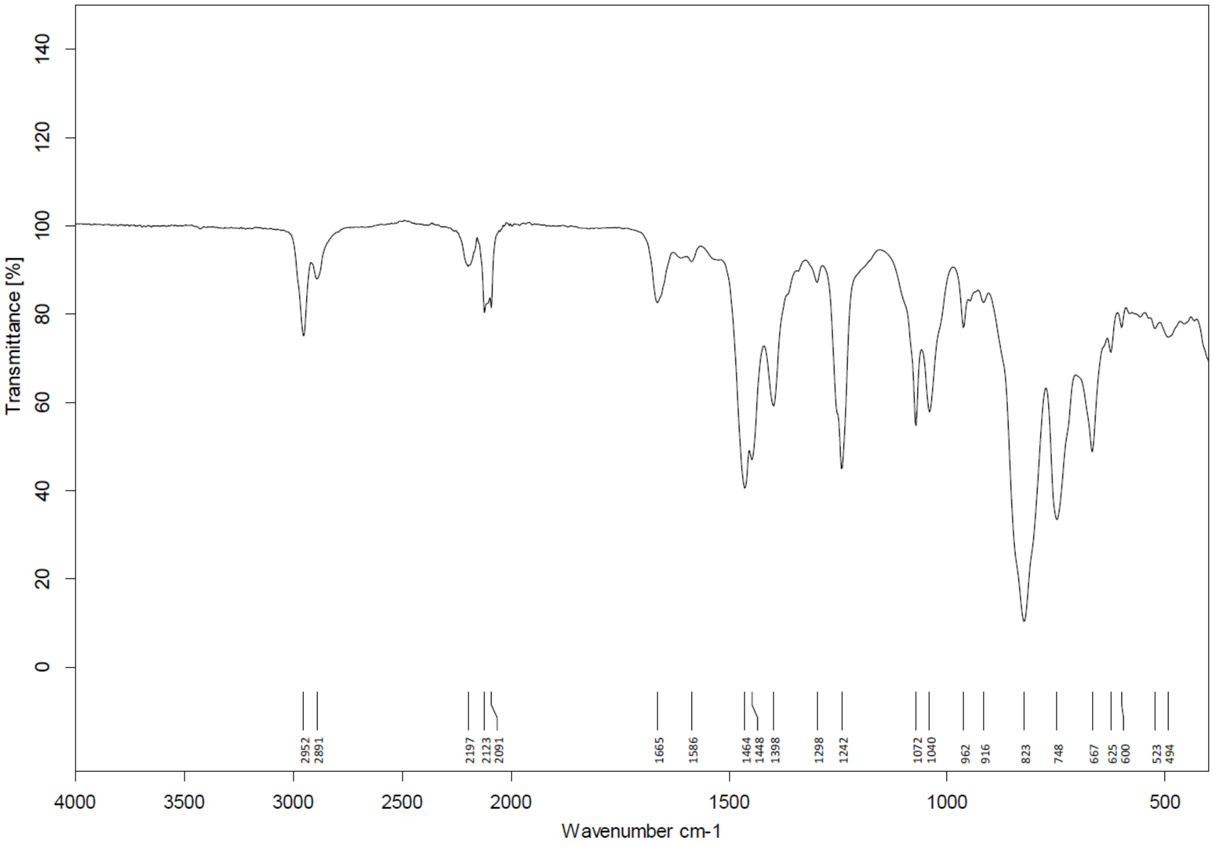

Figure S10: IR spectrum (ATR, ν [cm^{-1}]) of [(thf)$_2$Ca{EtC(N-SiMe$_3$)$_2$}(μ-I)]$_2$ (1b).

2.3 [(thf)$_3$Ca{MesC(N-*i*Pr)$_2$}I] (2)

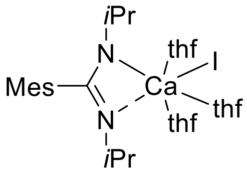

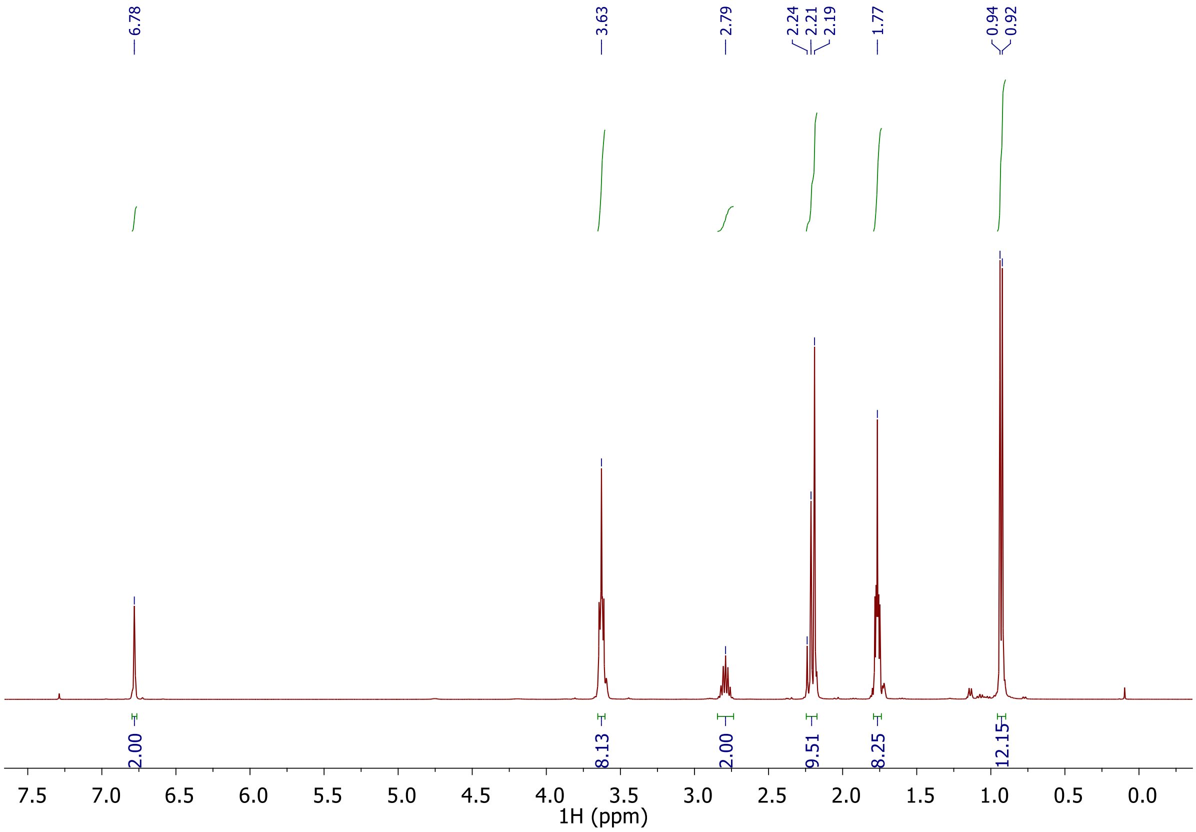

Figure S11: ^{1}H NMR spectrum (400 MHz, THF-d_8, 297K) of [(thf)$_3$Ca{MesC(N-iPr)$_2$}I] (2).

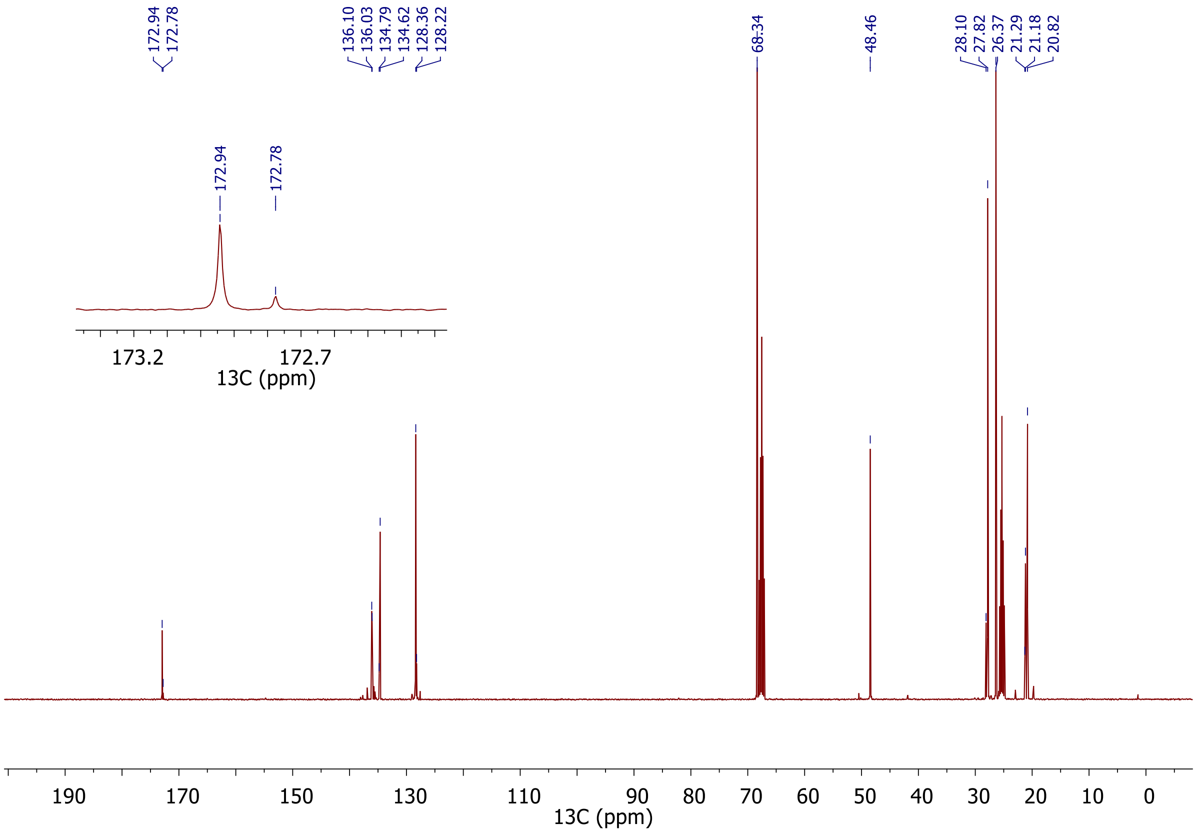

Figure S12: $^{13}C\{^1H\}$ NMR spectrum (101 MHz, THF-d_8, 297K) of [(thf)$_3$Ca{MesC(N-iPr)$_2$}I] (2).

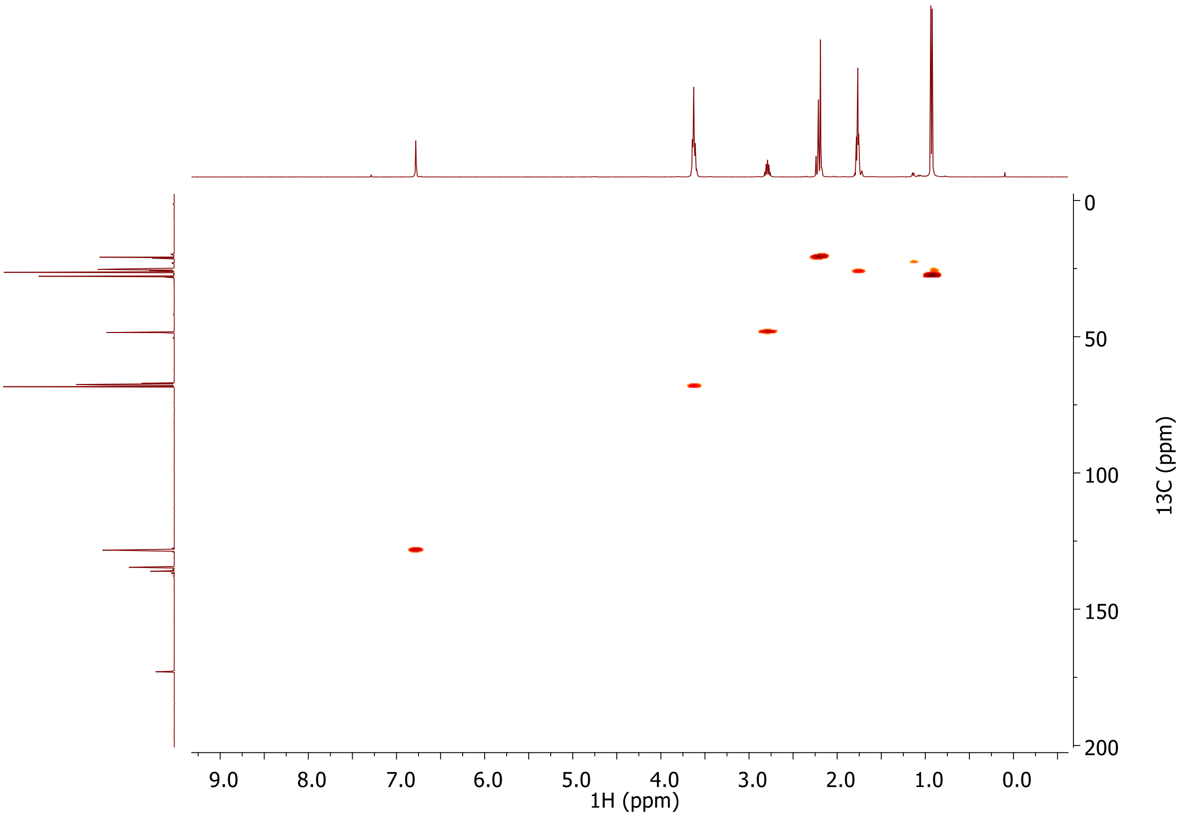

Figure S13: ASAP-HSQC-DEPT NMR spectrum (400 MHz, THF-d_8, 297K) of [(thf)$_3$Ca{MesC(N-iPr)$_2$}I] (2).

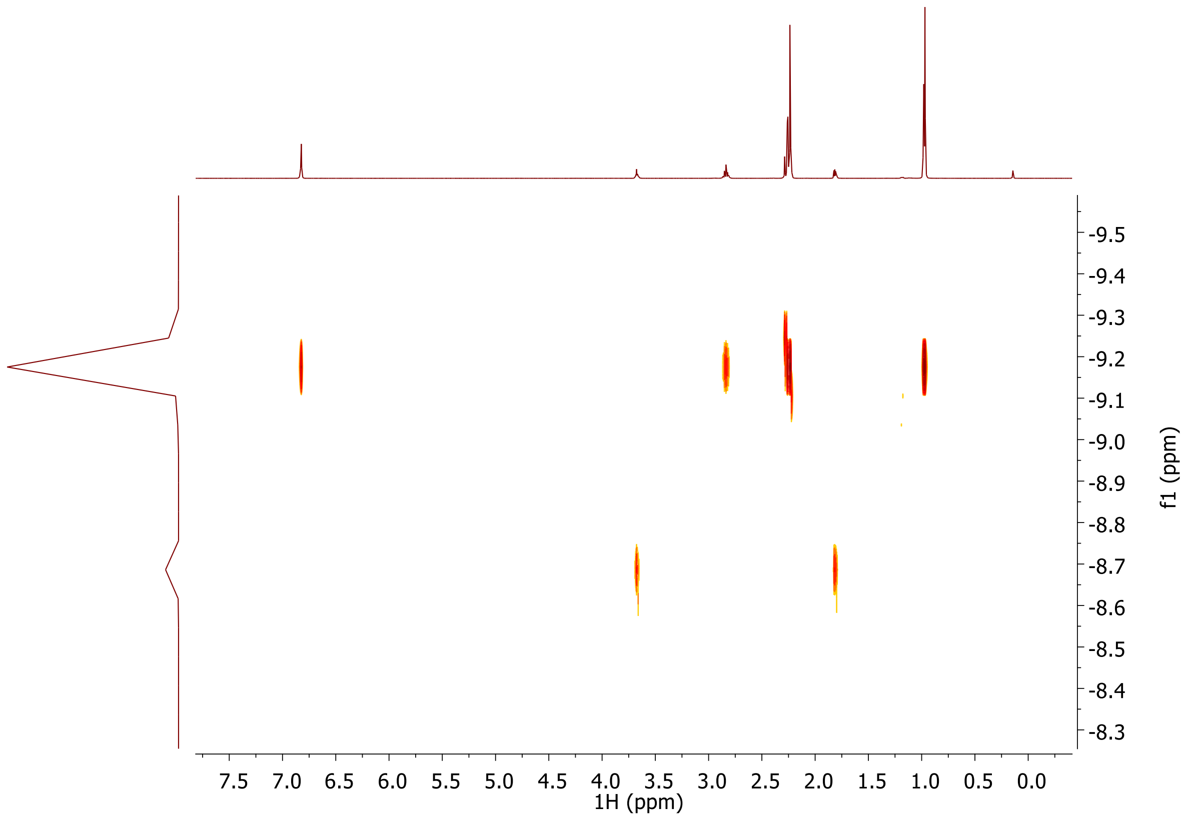

Figure S14: ^{1}H-DOSY NMR spectrum (400 MHz, THF-d_8, 297K) of [(thf)$_3$Ca{MesC(N-iPr)$_2$}I] (2).

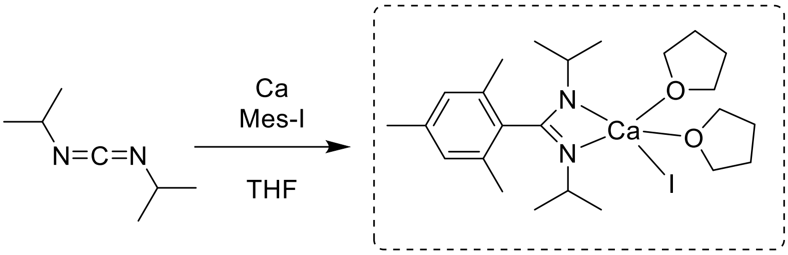

D(Ref) = 2.25×10^{-9} m^2/s (THF-d_7)
D(Subst) = 6.82×10^{-10} m^2/s
MW(calc) = 588 g/mol
MW(exp) = 556.6 g/mol
ΔMW = -5 %

Scheme S2: Calculation of the molar mass using ECC-DOSY method (dissipated spheres and ellipsoids (DSE) calibration curve).

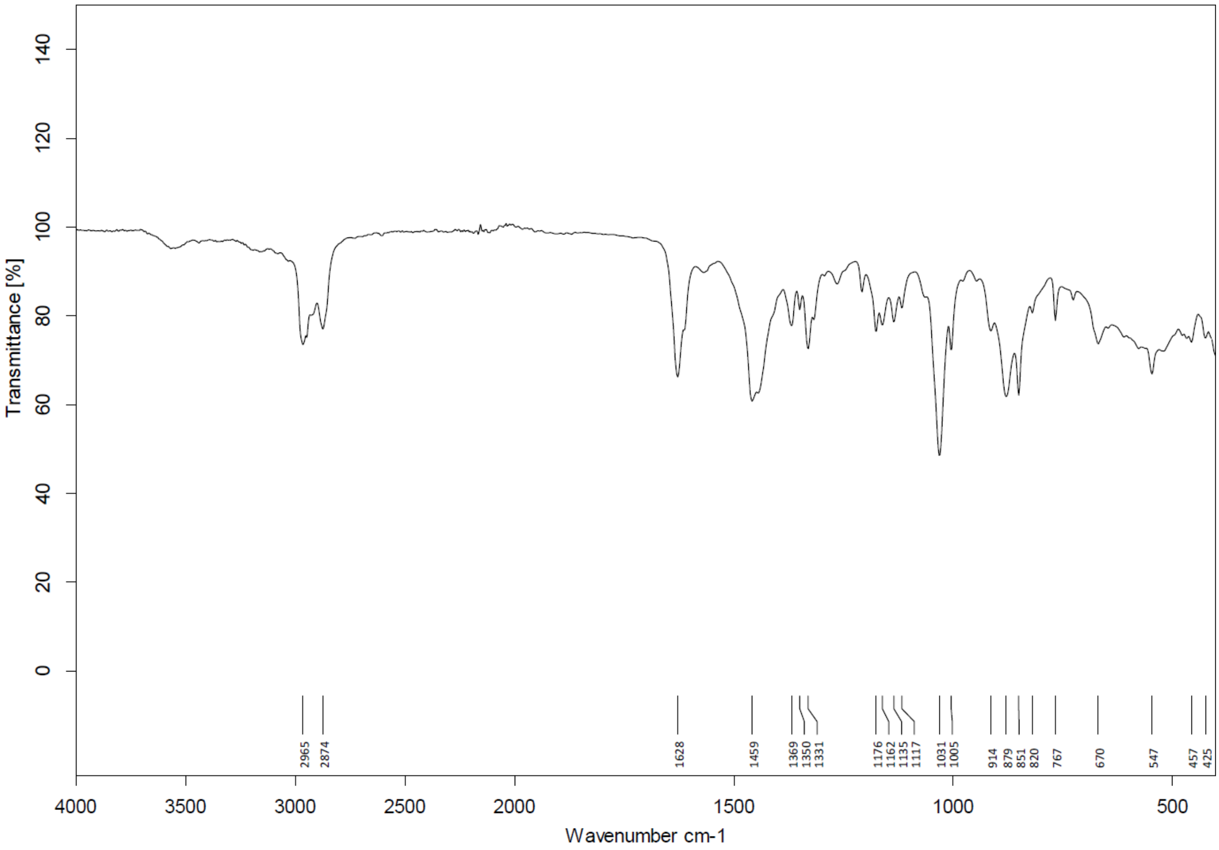

Figure S15: IR spectrum (ATR, ν [cm^{-1}]) of [(thf)$_3$Ca{MesC(N-iPr)$_2$}I] (2).

2.4 [(thf)$_2$Ca{AdC(N-C_6H_3-2,6-iPr2)(N-C_6H_3-2-(CPh_2)-6-($CHPh_2$))}·(thf)] (3)

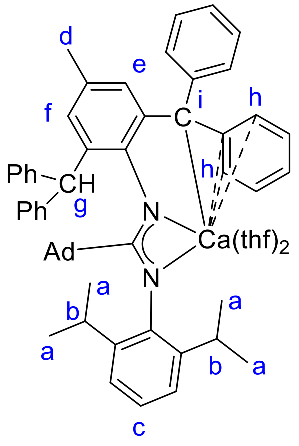

	δ ¹H [ppm]	δ ¹³C [ppm]
a	0.97, 0.88	23.6, 23.5
b	3.55, 3.08	29.5, 28.2
c	6.74	121.9
d	2.18	21.2
e	6.57	130.7
f	6.84	129.0
g	6.15	52.8
h	6.42, 5.55	117.0, 105.7
i	-	47.9
j	-	173.8
Ad	1.67-1.51, 1.28, 1.23-1.01	39.4, 28.2, 22.3
Ph	7.30-6.86	148-121

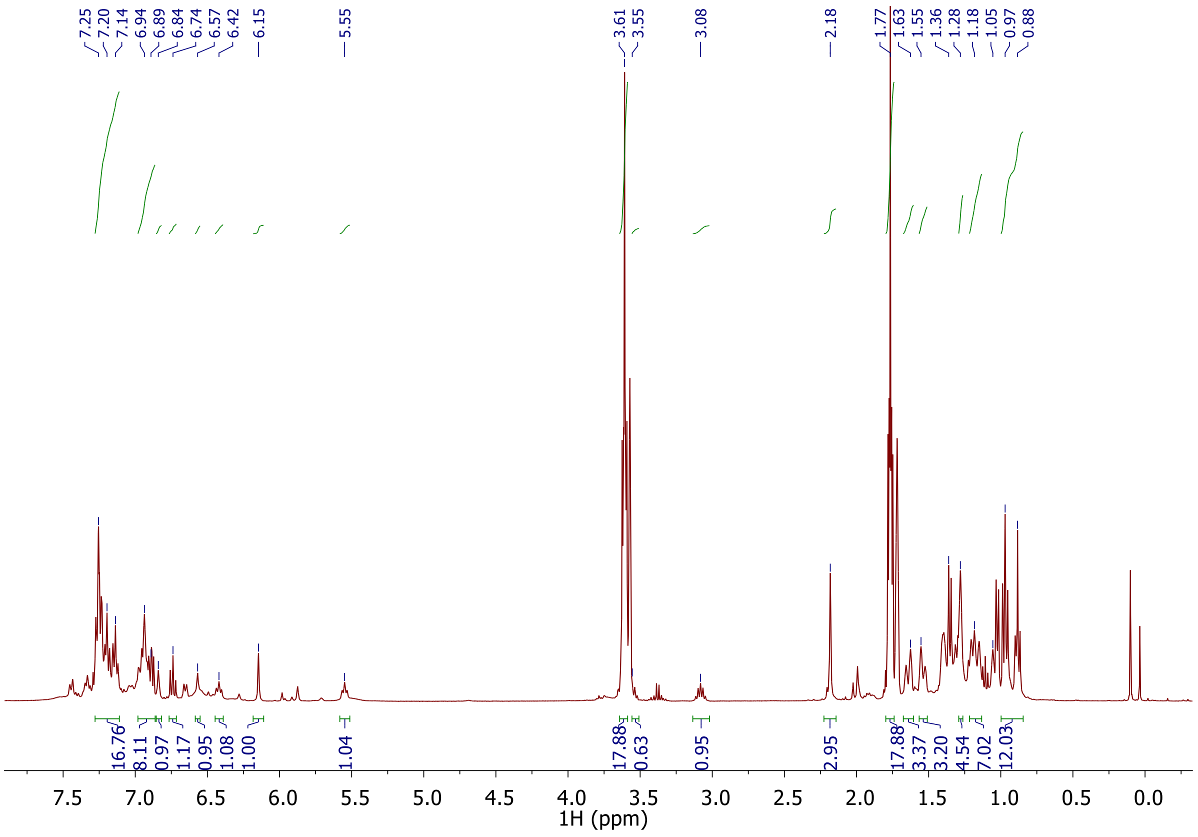

Figure S16: ¹H NMR spectrum (400 MHz, THF-d$_8$, 297K) of [(thf)$_2$Ca{AdC(N-C_6H_3-2,6-iPr2)(N-C_6H_3-2-(CPh_2)- 6-($CHPh_2$))}·(thf)] (3).

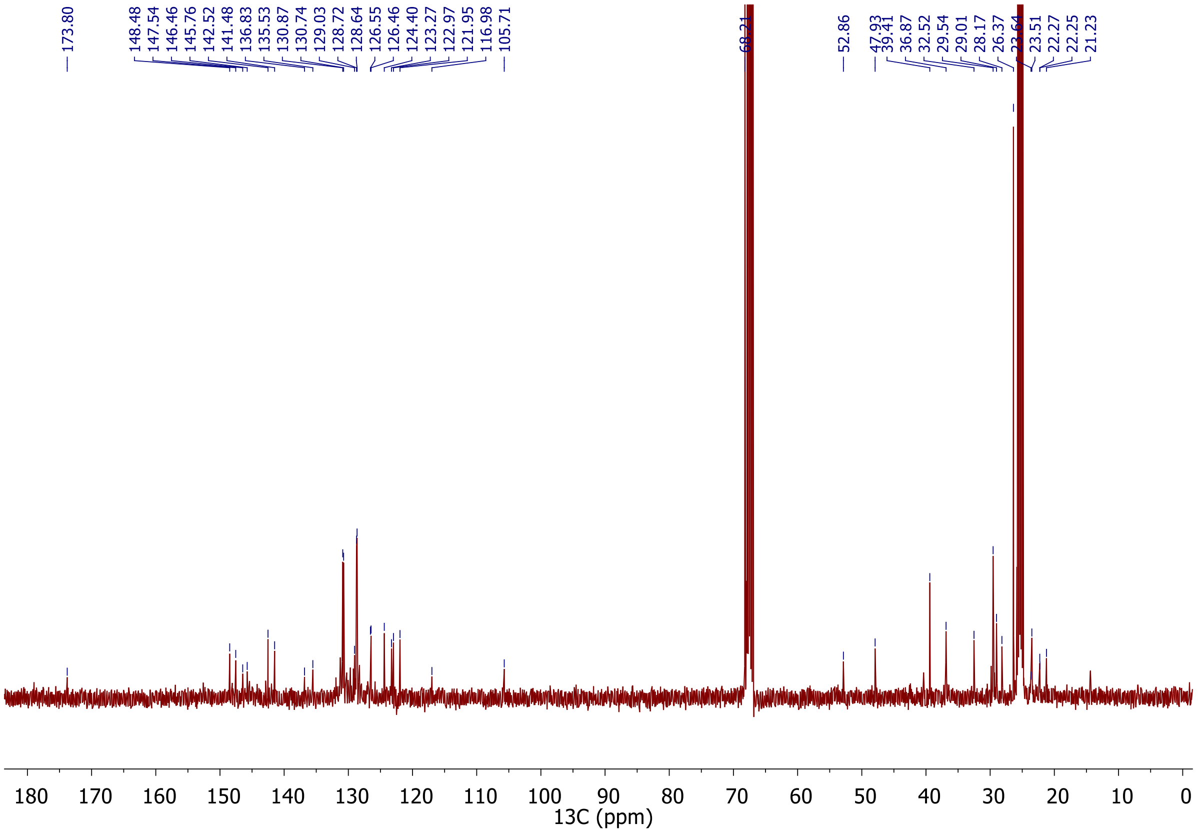

Figure S17: $^{13}C\{^{1}H\}$ NMR spectrum (101 MHz, THF-d_8, 297K) of [(thf)$_2$Ca{AdC(N-C_6H_3-2,6-iPr2)(N-C_6H_3-2-(CPh$_2$)- 6-(CHPh$_2$))}·(thf)] (3).

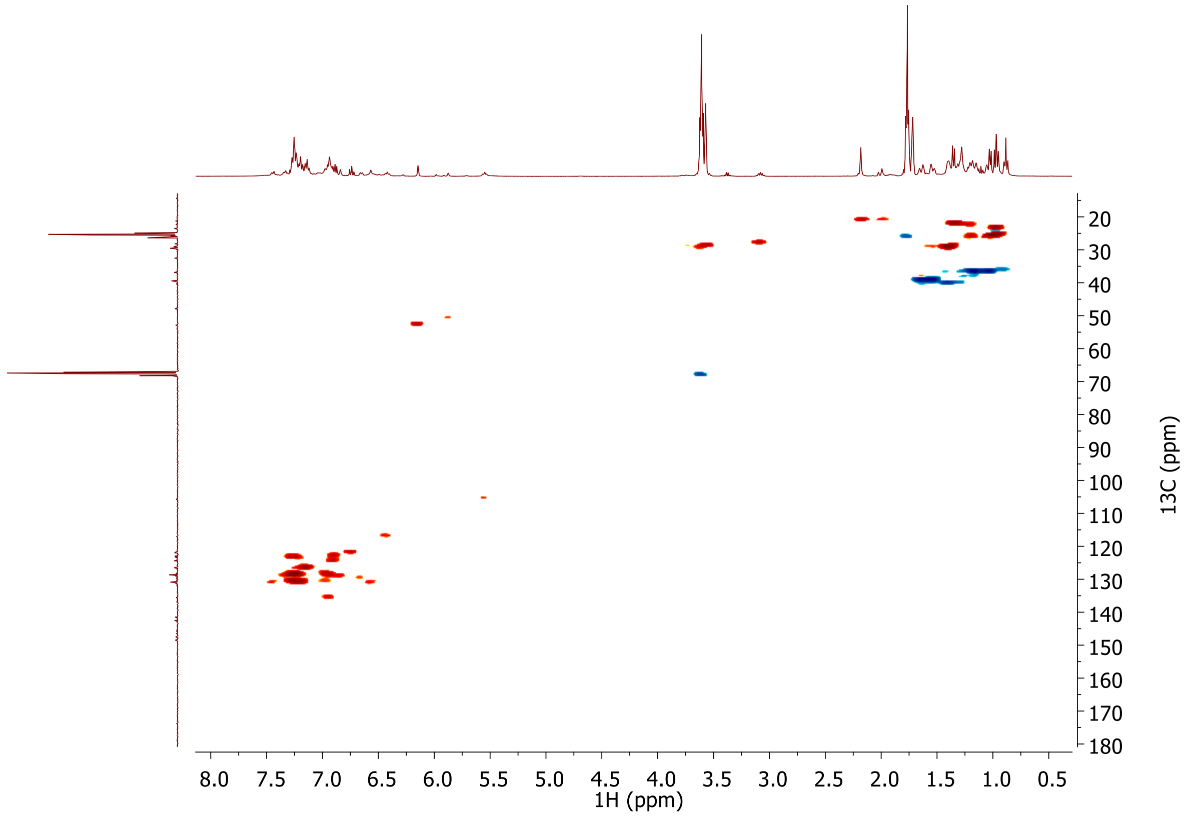

Figure S18: ASAP-HSQC-DEPT NMR spectrum (400 MHz, THF-d_8, 297K) of [(thf)$_2$Ca{AdC(N-C_6H_3-2,6-iPr2)(N-C_6H_3-2-(CPh$_2$)- 6-(CHPh$_2$))}·(thf)] (3).

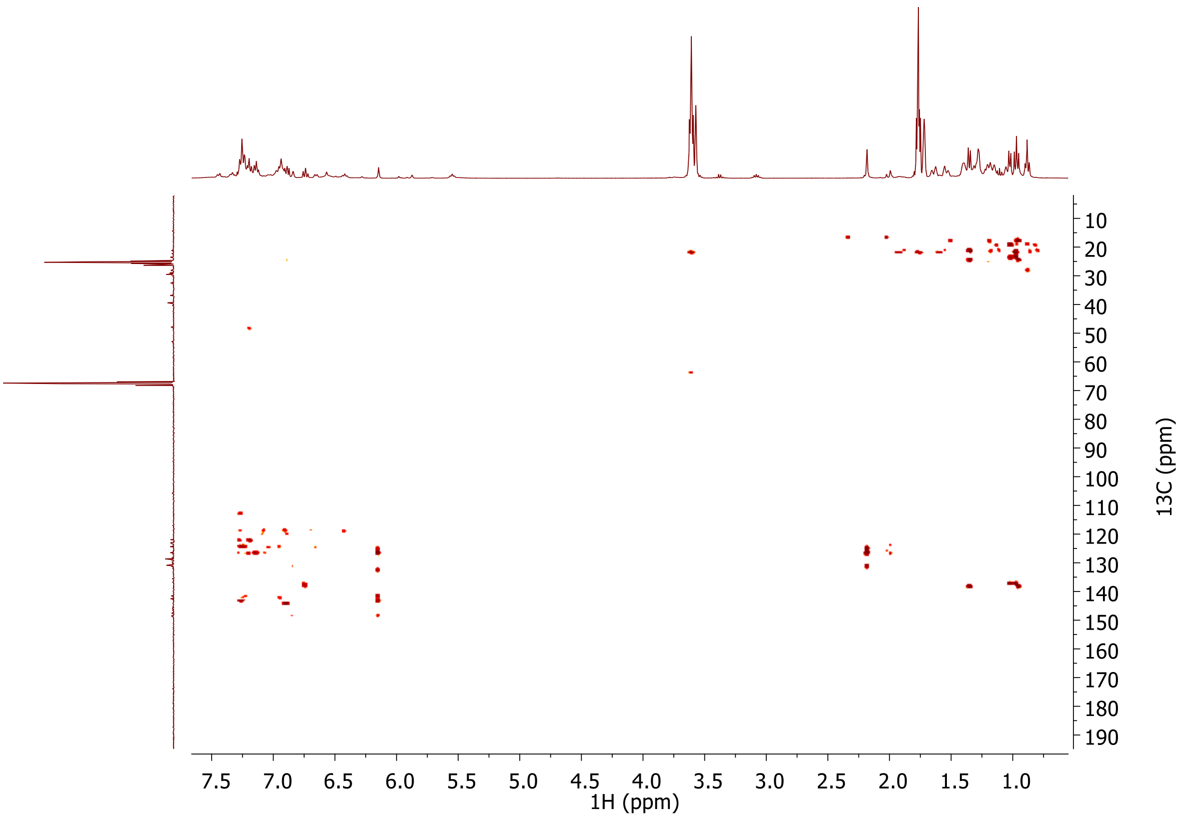

Figure S19: HMBC NMR spectrum (400 MHz, THF-d_8, 297K) of [(thf)$_2$Ca{AdC(N-C_6H_3-2,6-iPr2)(N-C_6H_3-2-(CPh_2)-6-($CHPh_2$))}·(thf)] (3).

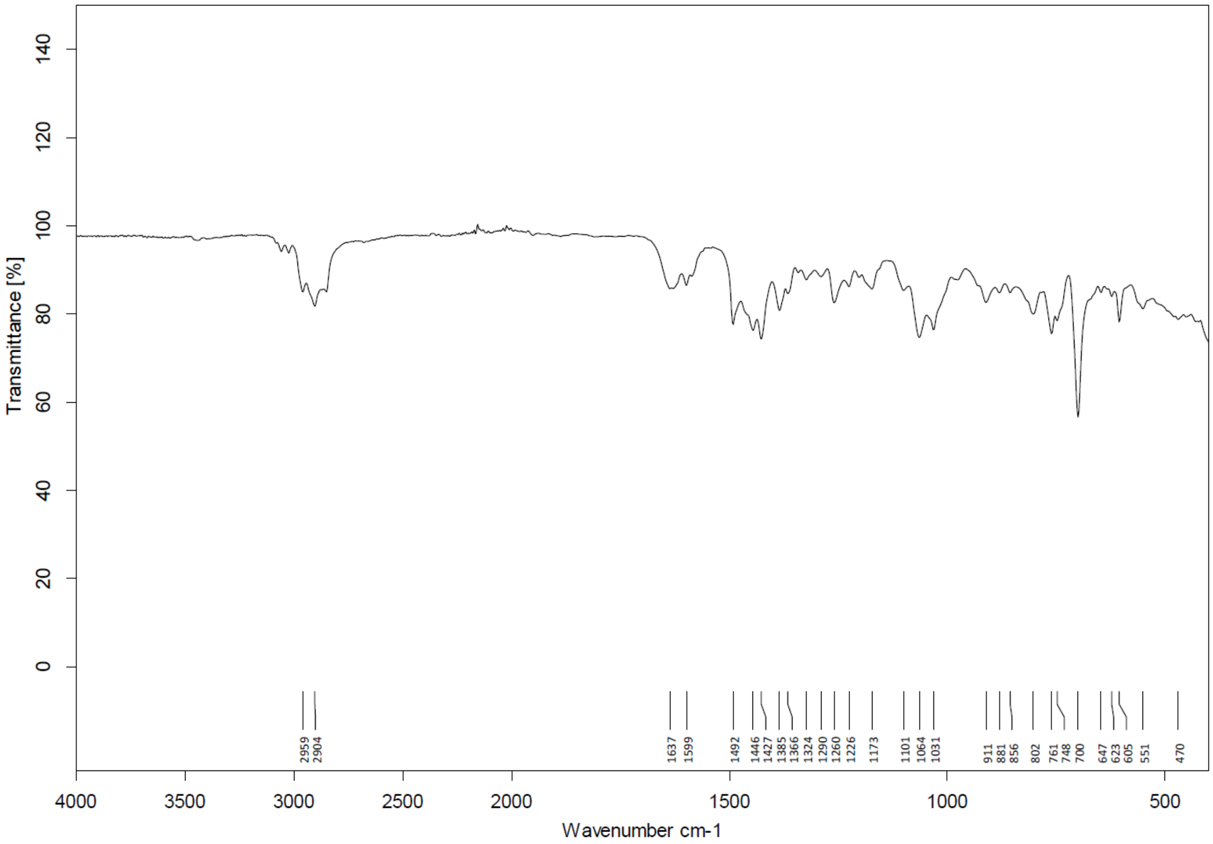

Figure S20: IR spectrum (ATR, ν [cm^{-1}]) of [(thf)$_2$Ca{AdC(N-C_6H_3-2,6-iPr$_2$)(N-C_6H_3-2-(CPh_2)-6-($CHPh_2$))}·(thf)] (3).

2.5 iGAM Screening

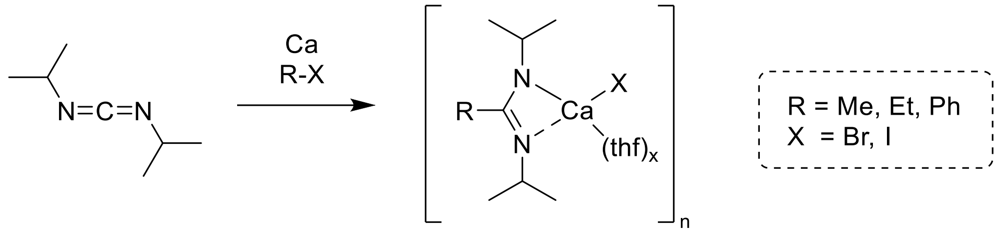

2.5.1 Diisopropylcarbodiimide + methyl iodide

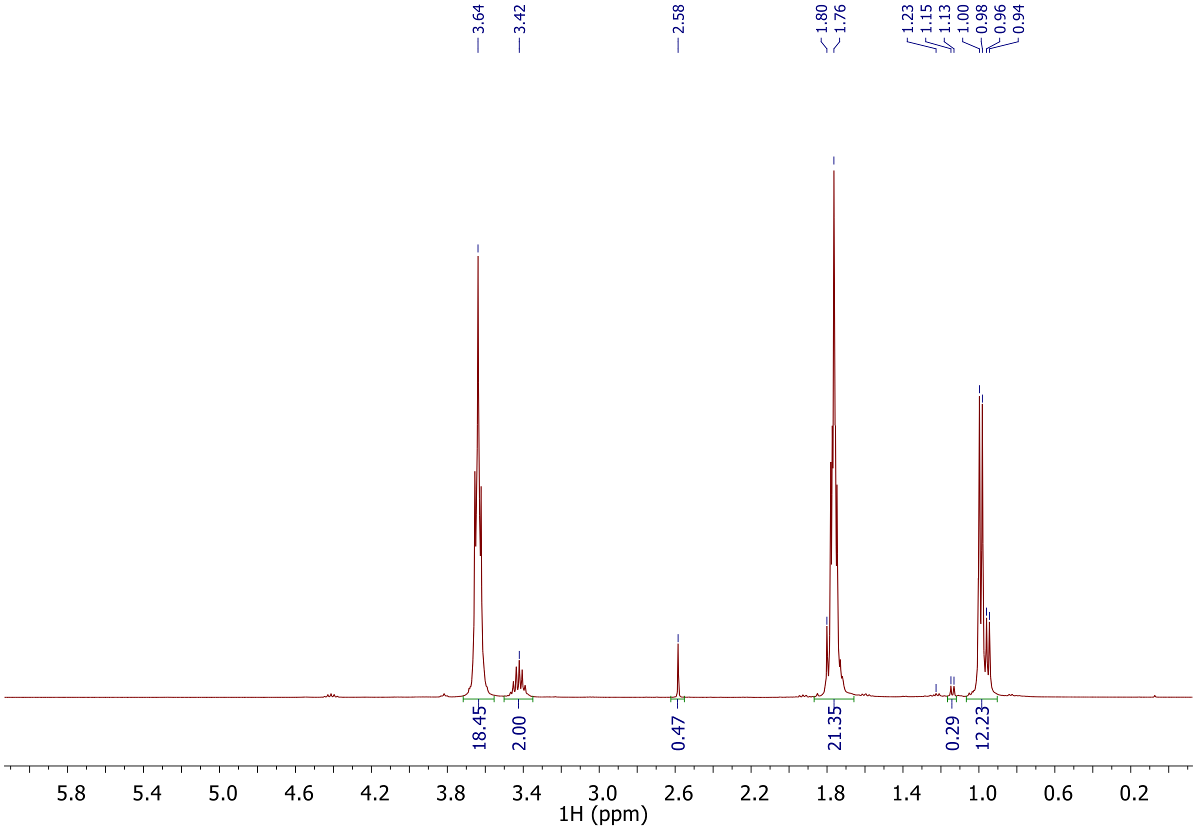

Figure S21: ¹H NMR spectrum (400 MHz, THF-d_8, 297K) of the iGAM between Diisopropylcarbodiimide and Me-I. Reaction mixture: Edukt (1.15 ppm), Hydrolysis (1.23 ppm).

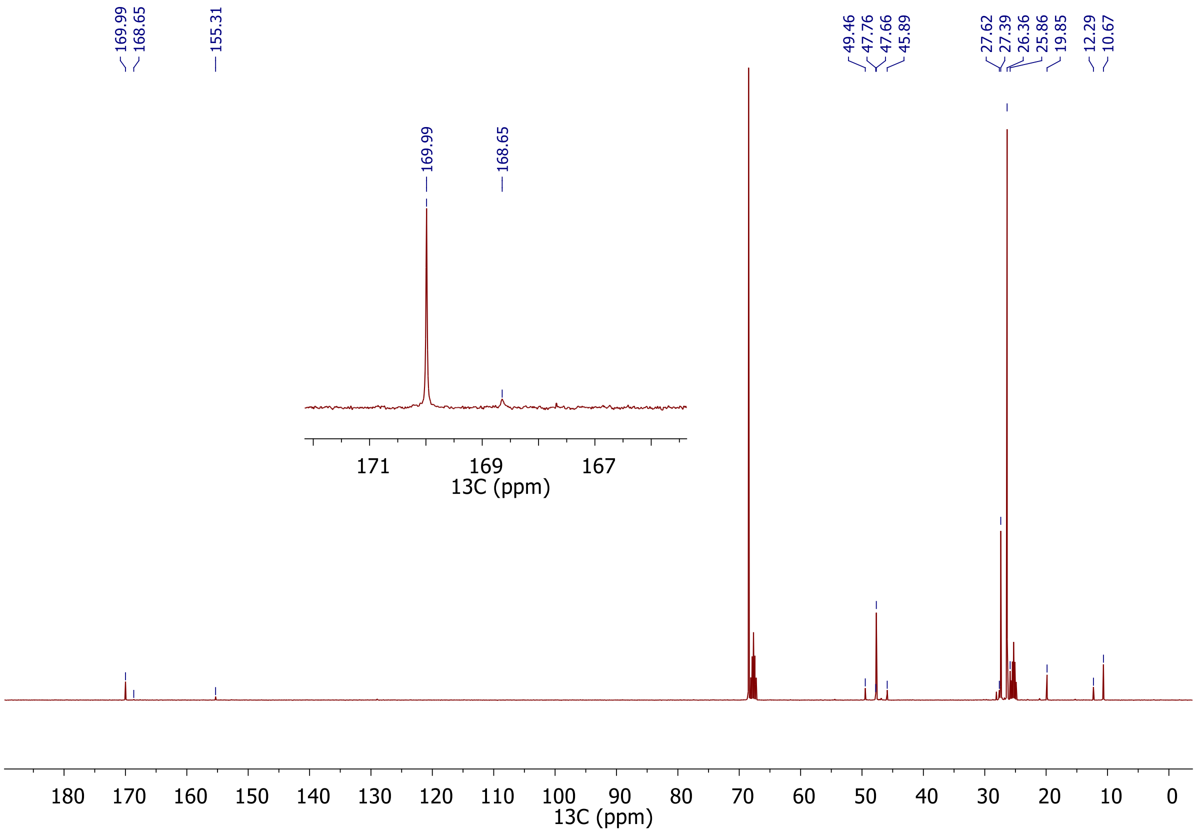

Figure S22: ^{13}C NMR spectrum (101 MHz, THF-d_8, 297K) of the iGAM between Diisopropylcarbodiimide and Me-I. Reaction mixture: Hydrolysis (155.3, 45.9, 19.9 ppm).

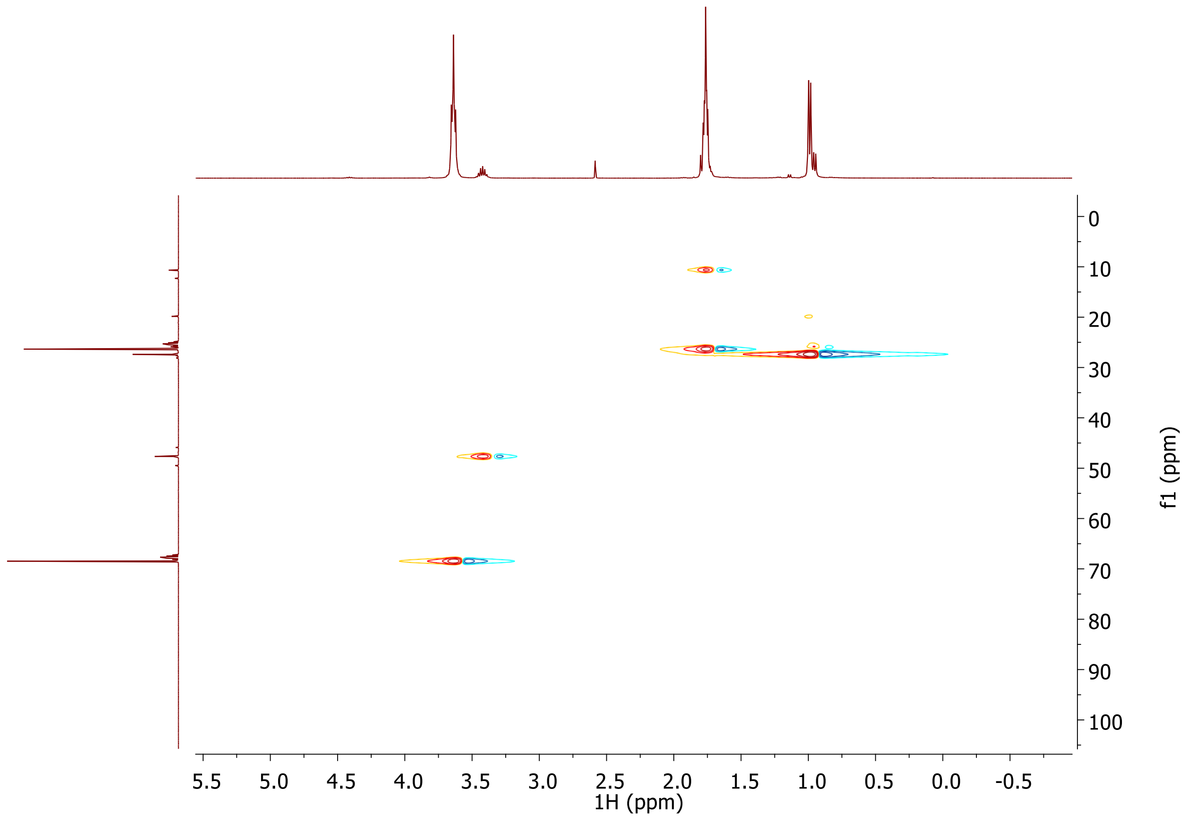

Figure S 23: ASAP-HSQC-DEPT-NMR spectrum (400 MHz, THF-d_8, 297K) of the iGAM between Diisopropylcarbodiimide and Me-I.

2.5.2 Diisopropylcarbodiimide + ethyl bromide

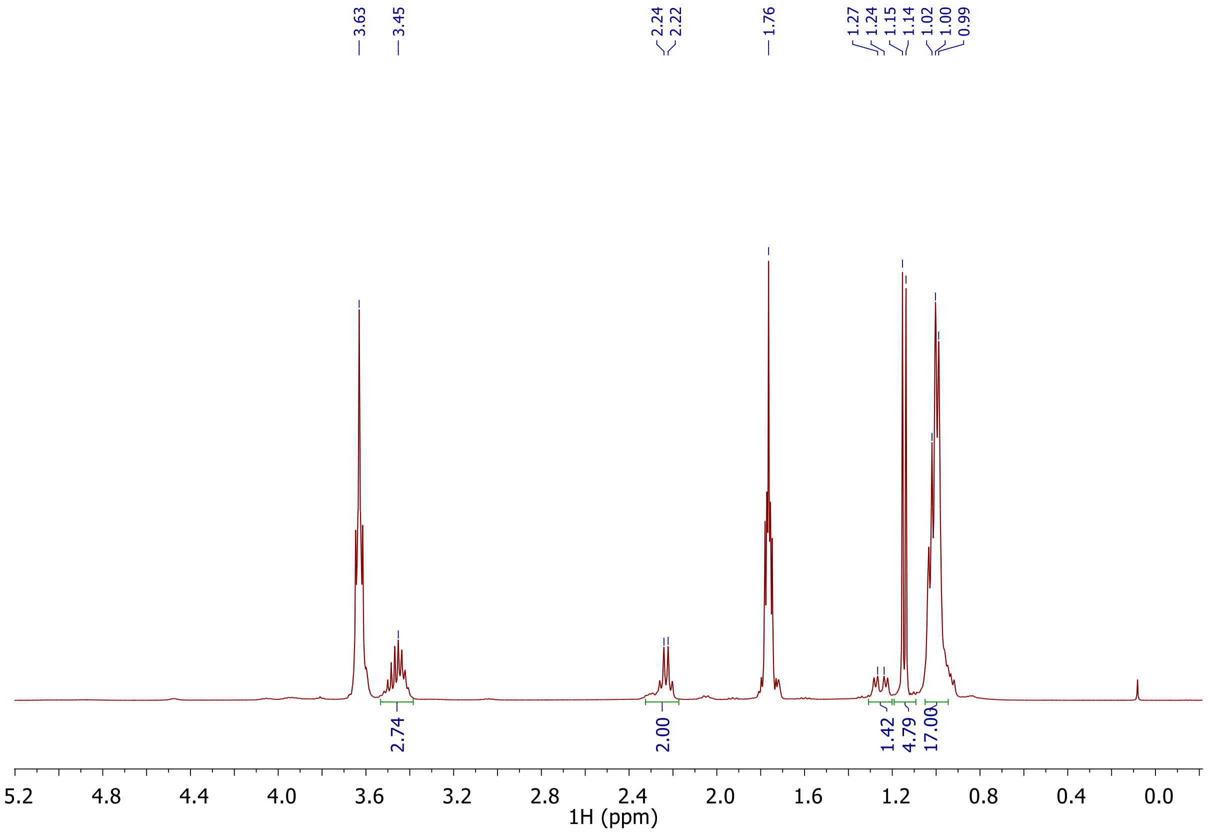

Figure S24: ^{1}H NMR spectrum (400 MHz, THF-d8, 297K) of the iGAM between Diisopropylcarbodiimide and Et-Br. Reaction mixture: Edukt (1.15 ppm), Hydrolysis (1.27-1.24 ppm).

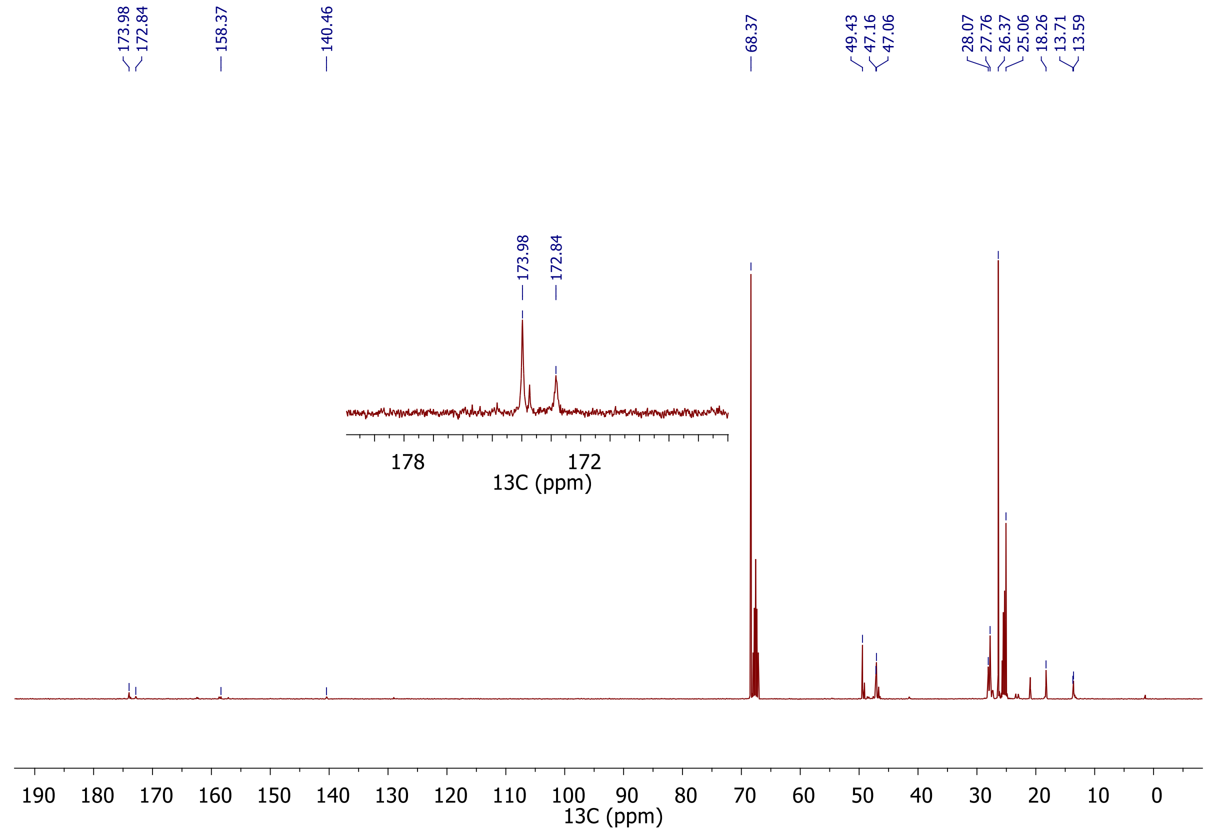

Figure S25: ^{13}C NMR spectrum (101 MHz, THF-d8, 297K) of the iGAM between Diisopropylcarbodiimide and Et-Br. Reaction mixture: Educt (140.5 ppm), Hydrolysis (158.4 ppm).

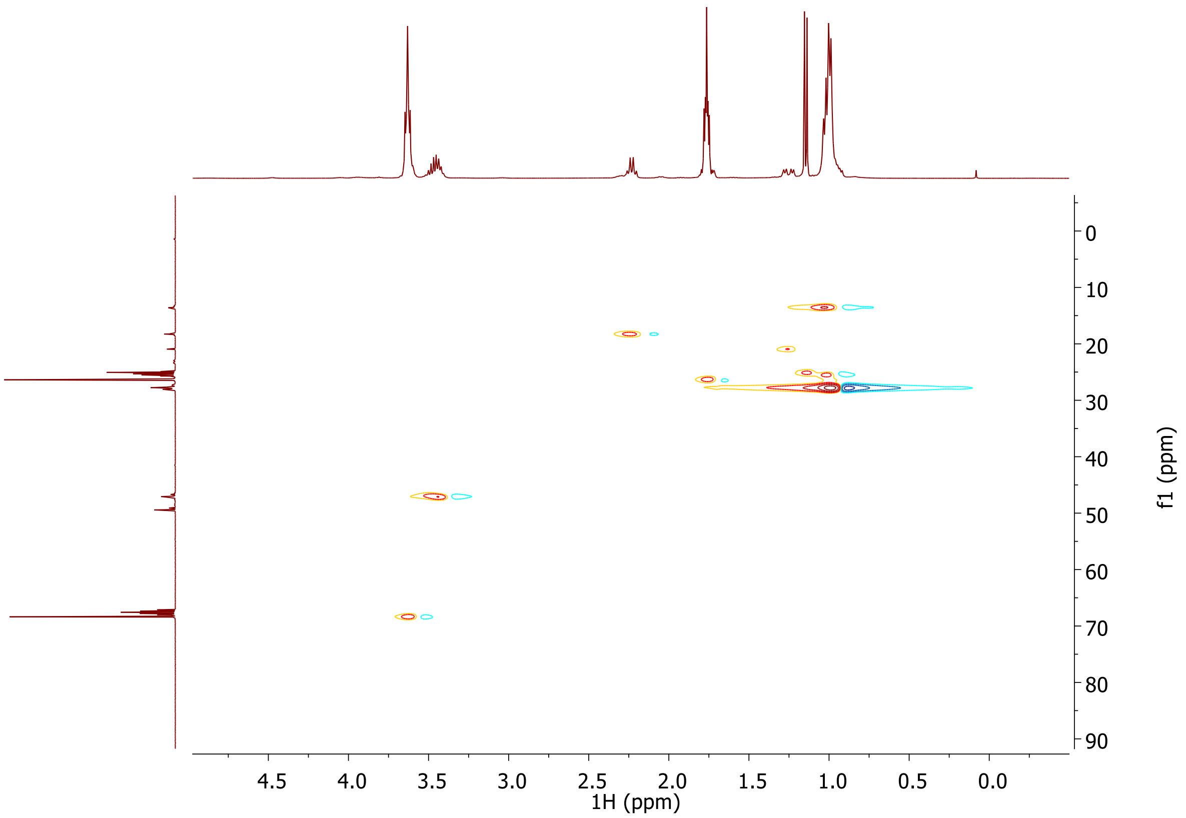

Figure S26: ASAP-HSQC-DEPT-NMR spectrum (400 MHz, THF-d_8, 297K) of the iGAM between Diisopropylcarbodiimide and Et-Br.

2.5.3 Diisopropylcarbodiimide + ethyl iodide

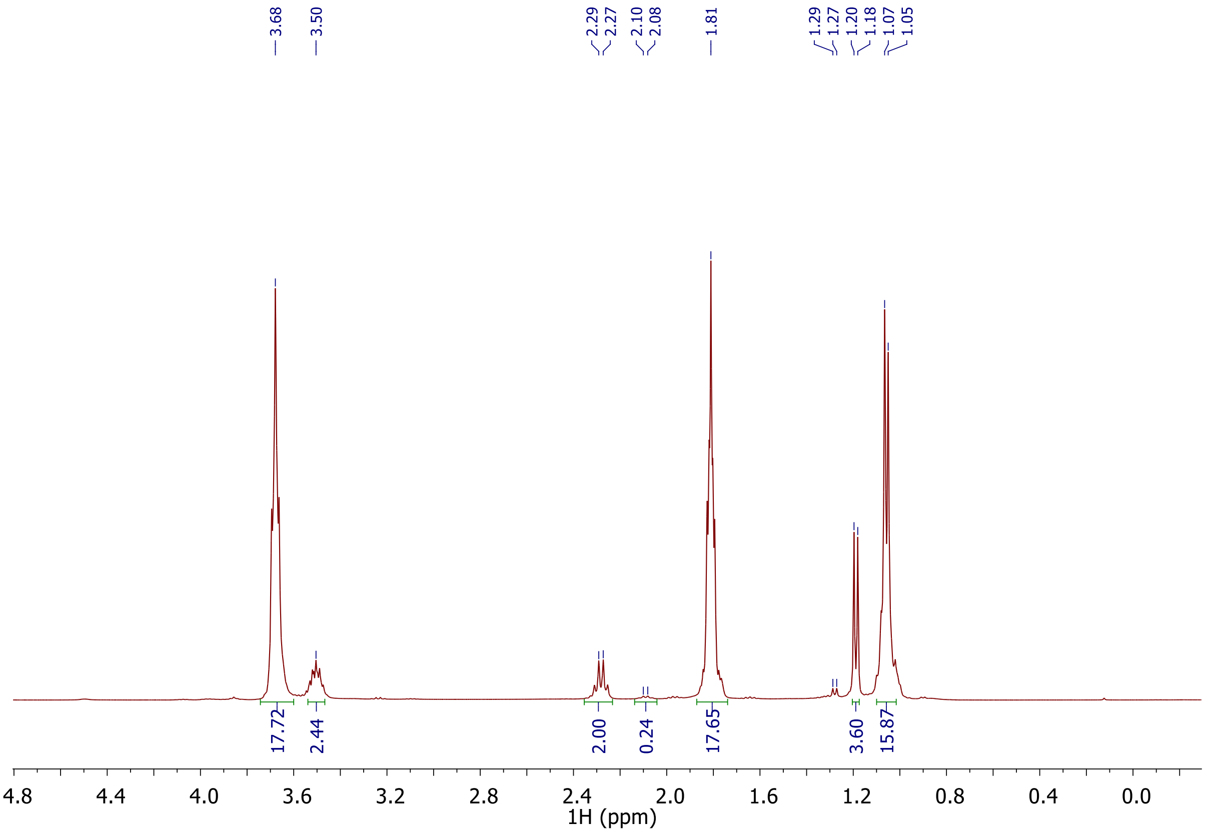

Figure S27: ^{1}H NMR spectrum (400 MHz, THF-d$_8$, 297K) of the iGAM between Diisopropylcarbodiimide and Et-I. Reaction mixture: Edukt (1.19 ppm), Hydrolysis (1.28 ppm).

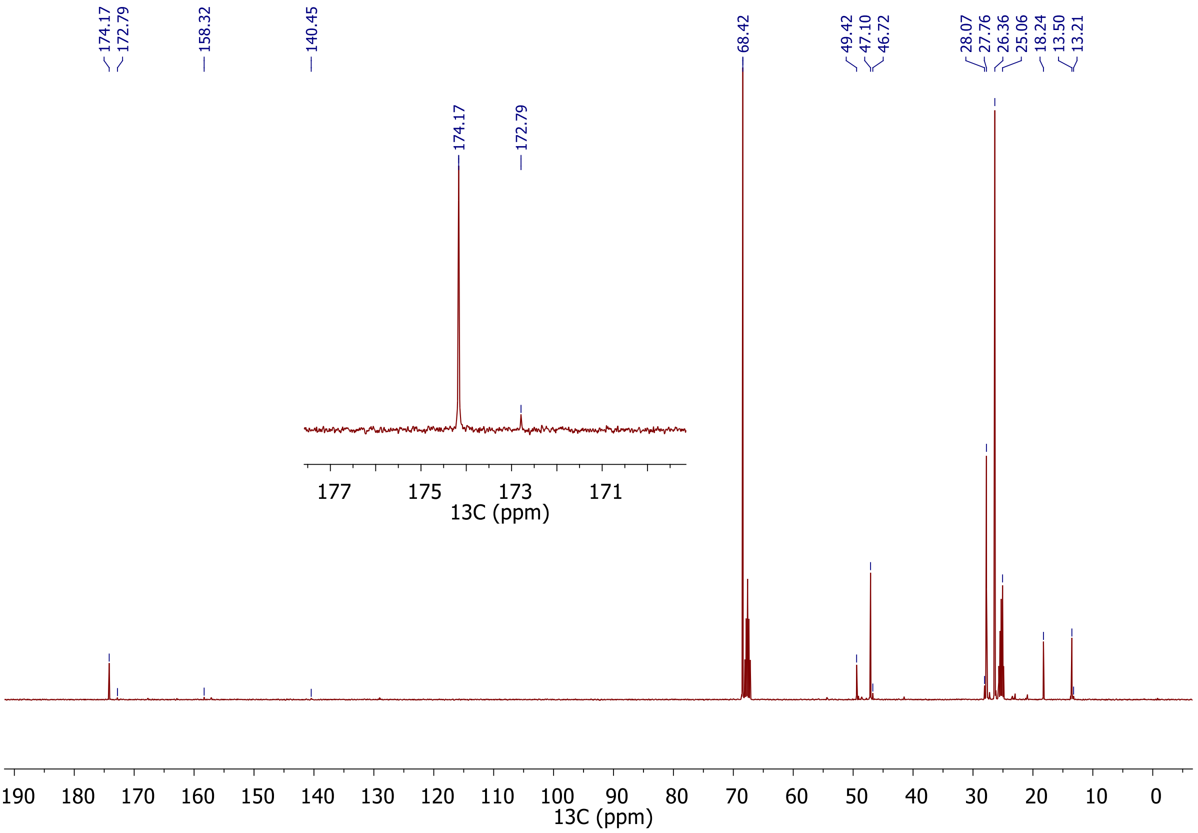

Figure S28: ^{13}C NMR spectrum (101 MHz, THF-d_8, 297K) of the iGAM between Diisopropylcarbodiimide and Et-I. Reaction mixture: Educt (140.5 ppm), Hydrolysis (158.3 ppm).

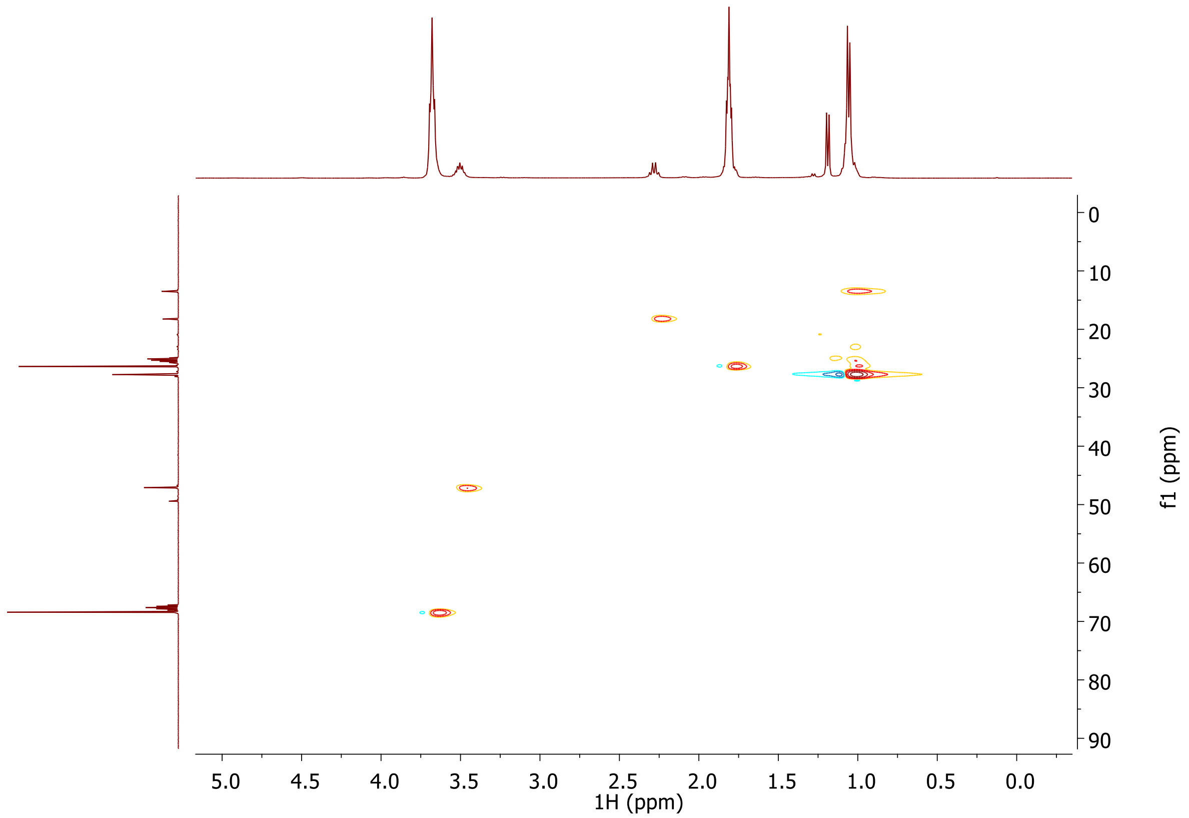

Figure S29: ASAP-HSQC-DEPT-NMR spectrum (400 MHz, THF-d_8, 297K) of the iGAM between Diisopropylcarbodiimide and Et-I.

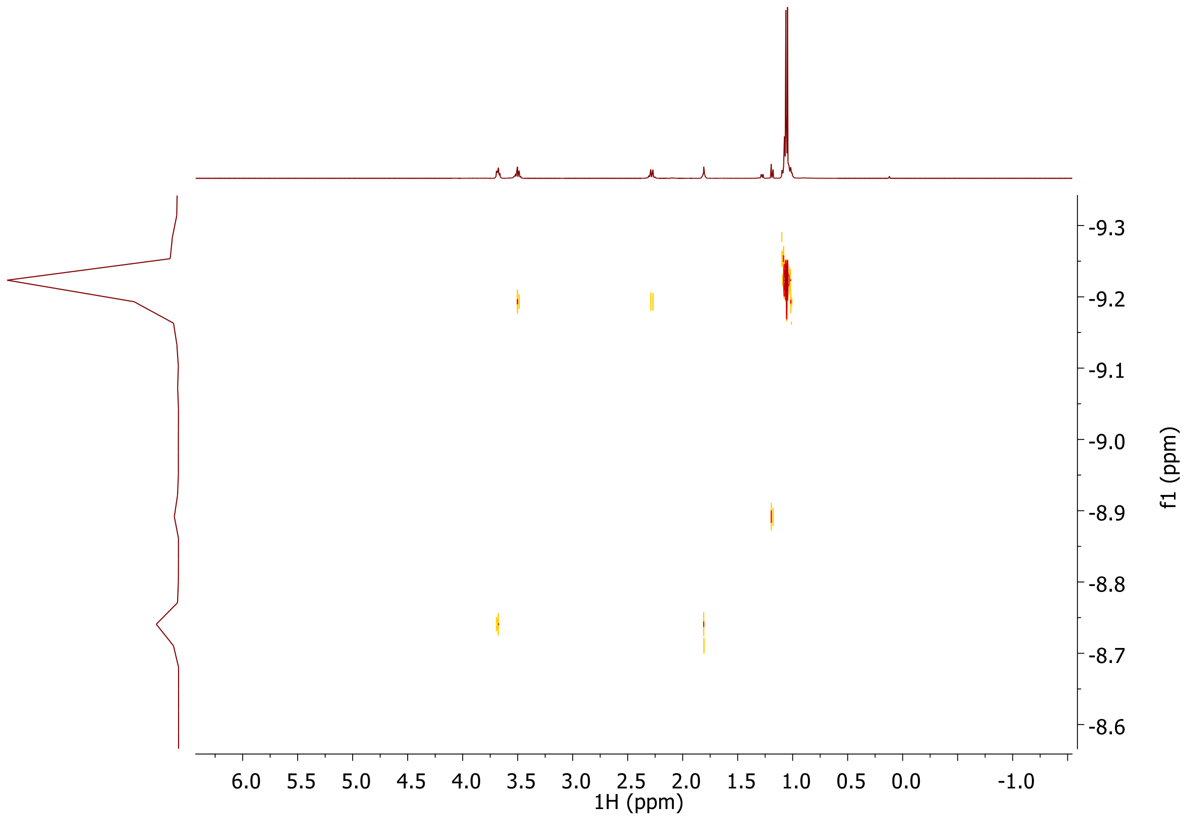

Figure S30: 1H NMR spectrum (400 MHz, THF-d8, 297K) of the iGAM between Diisopropylcarbodiimide and Et-I.

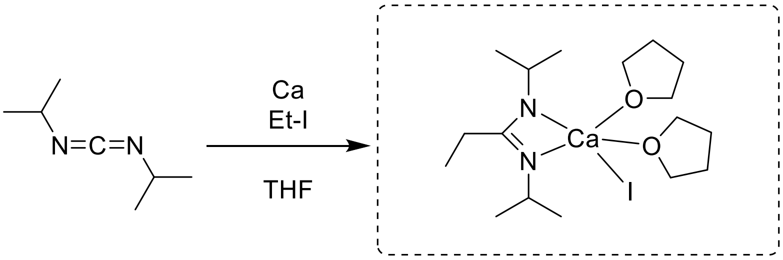

D(Ref) = 1.82×10^{-9} m^2/s (THF-d_7)
D(Subst) = 6.22×10^{-10} m^2/s
MW(calc) = 479 g/mol
MW(exp) = 466.5 g/mol
ΔMW = -3 %

Scheme S3: Calculation of the molar mass using ECC-DOSY method (dissipated spheres and ellipsoids (DSE) calibration curve).

2.5.4 Diisopropylcarbodiimide + phenyl iodide

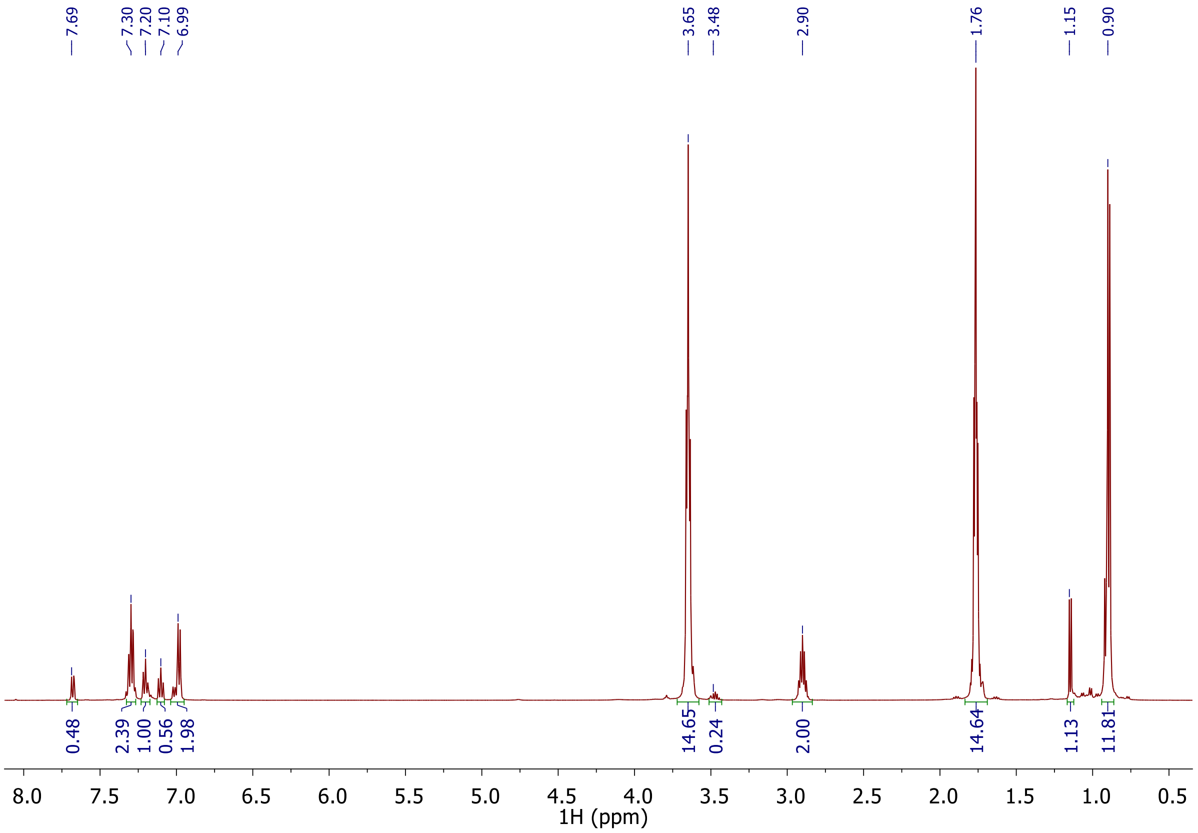

Figure S31: ^{1}H NMR spectrum (400 MHz, THF-d8, 297K) of the iGAM between Diisopropylcarbodiimide and Ph-I. Reaction mixture: Educt (1.13 ppm), Ph-I (7.69, 7.30 (overlaps with product), 7.10 ppm).

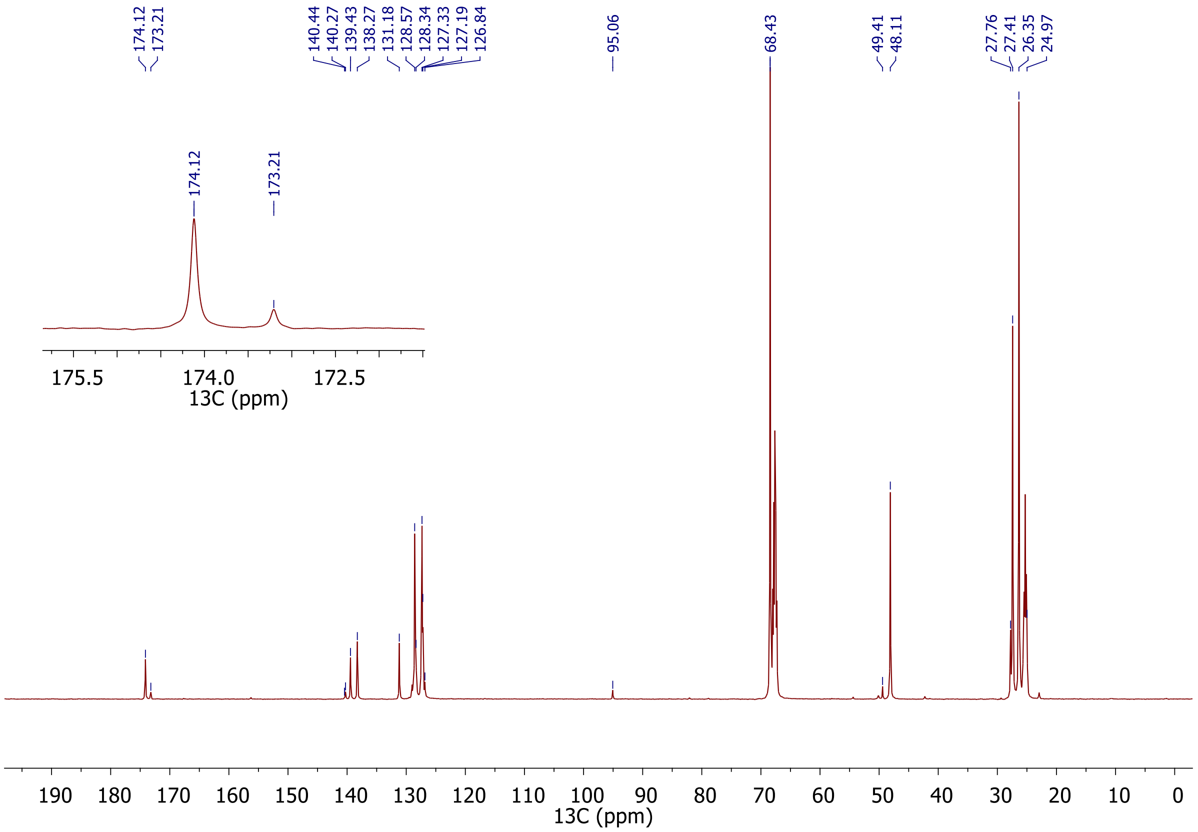

Figure S32: ^{13}C NMR spectrum (101 MHz, THF-d8, 297K) of the iGAM between Diisopropylcarbodiimide and Ph-I. Reaction mixture: Educt (140.4 ppm), Ph-I (138.2, 131.2, 128-127, 95.1 ppm).

2.6 Protolysed iGAM reactions

2.6.1 Protolysis of [(thf)$_x$Ca{MeC(N-^{i}Pr)$_2$}(I)]$_n$

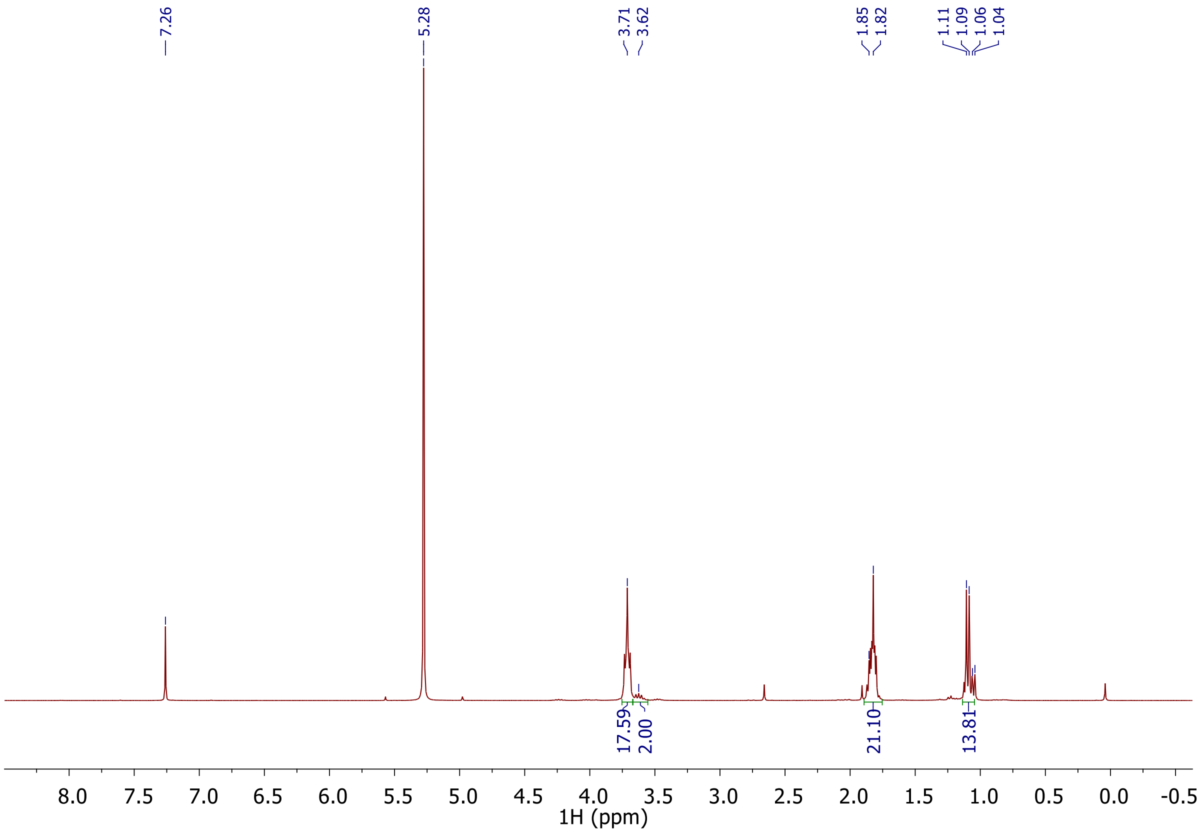

Figure S33: ^{1}H NMR spectrum (300 MHz, $CDCl_3$, 297K) of N,N'-Bis(diisopropyl)acetimidamide (reaction mixture). (Dichloromethane: 5.28 ppm).

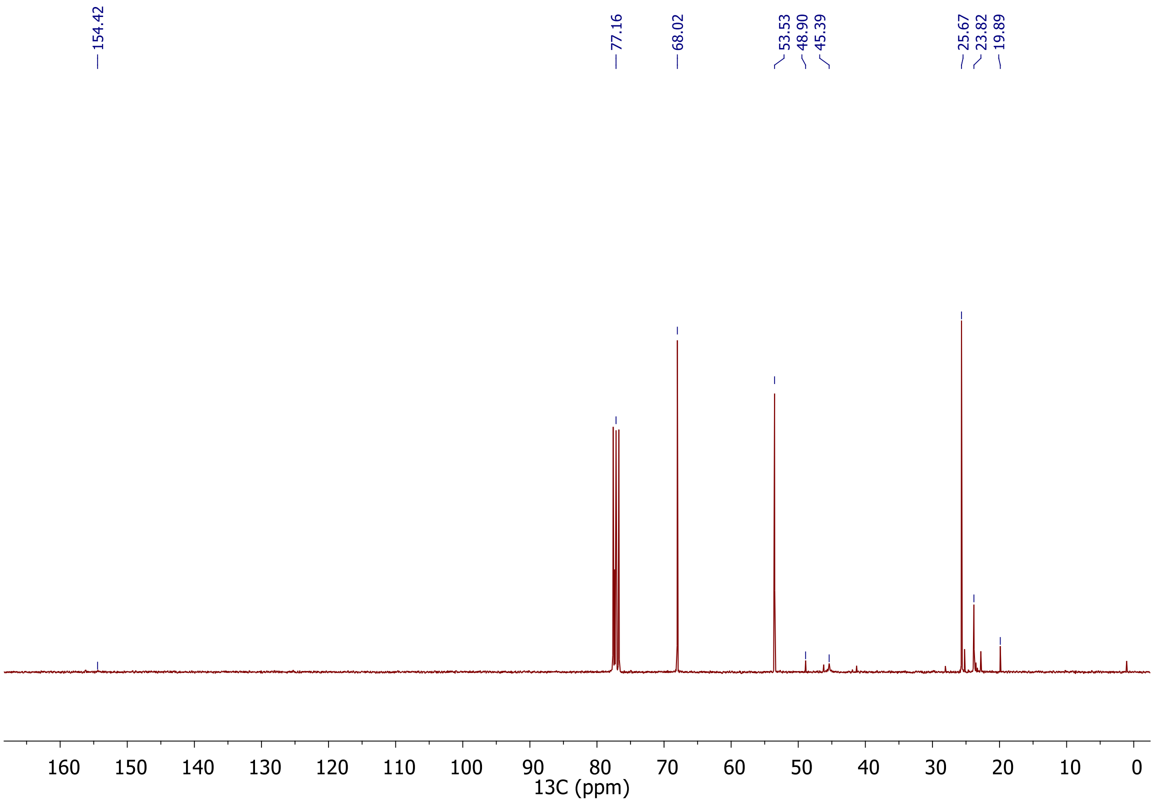

Figure S34: ^{13}C NMR spectrum (75.5 MHz, $CDCl_3$, 297K) of N,N'-Bis(diisopropyl)acetimidamide (reaction mixture). (Dichloromethane: 53.53 ppm, THF: 68.0, 25.7 ppm).

2.6.2 Protolysis of $[(thf)_xCa\{EtC(N\text{-}^iPr)_2\}(I)]_n$

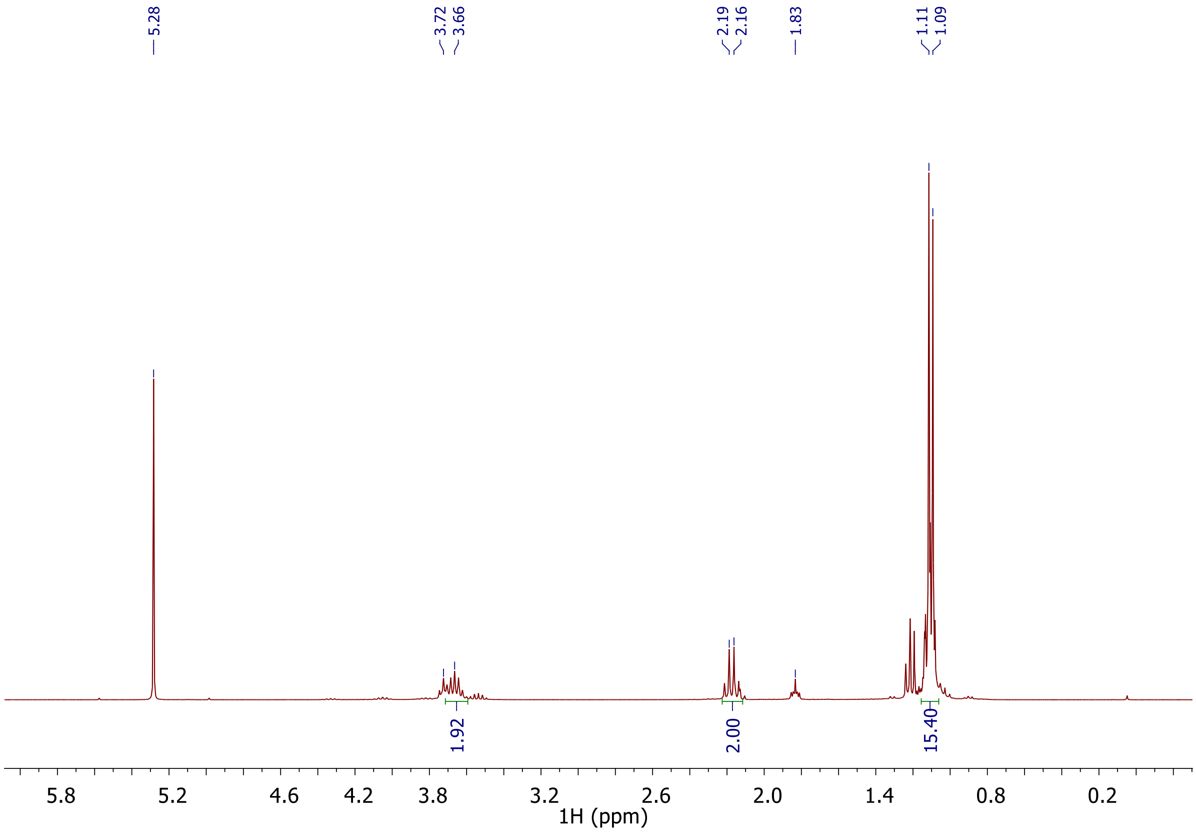

Figure S35: ^{1}H NMR spectrum (300 MHz, $CDCl_3$, 297K) of N,N'-Bis(diisopropyl)propionimidamide (reaction mixture). (Dichloromethane: 5.28 ppm).

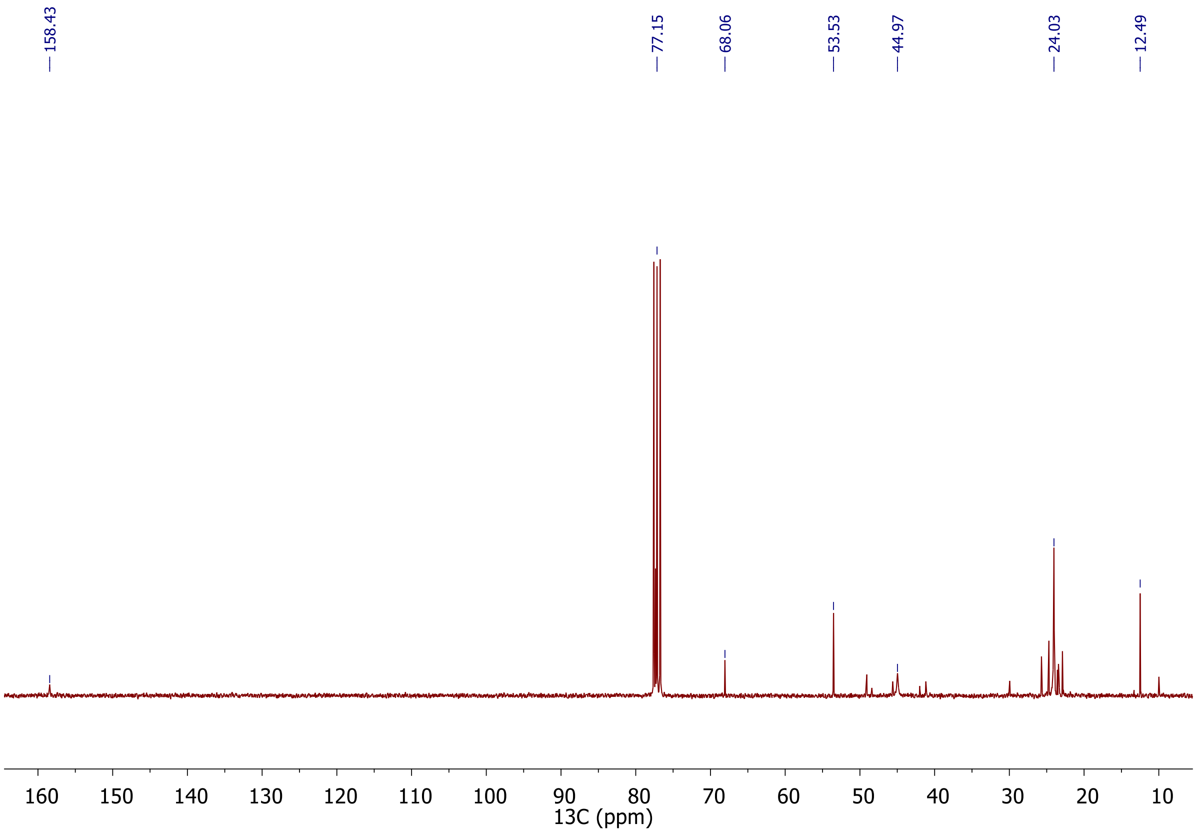

Figure S36: ^{13}C NMR spectrum (75.5 MHz, $CDCl_3$, 297K) of N,N'-Bis(diisopropyl)propionimidamide (reaction mixture). (Dichloromethane: 53.53 ppm, THF: 68.0, 25.7 ppm).

2.6.3 Protolysis of $[(thf)_xCa\{PhC(N\text{-}^iPr)_2\}(I)]_n$

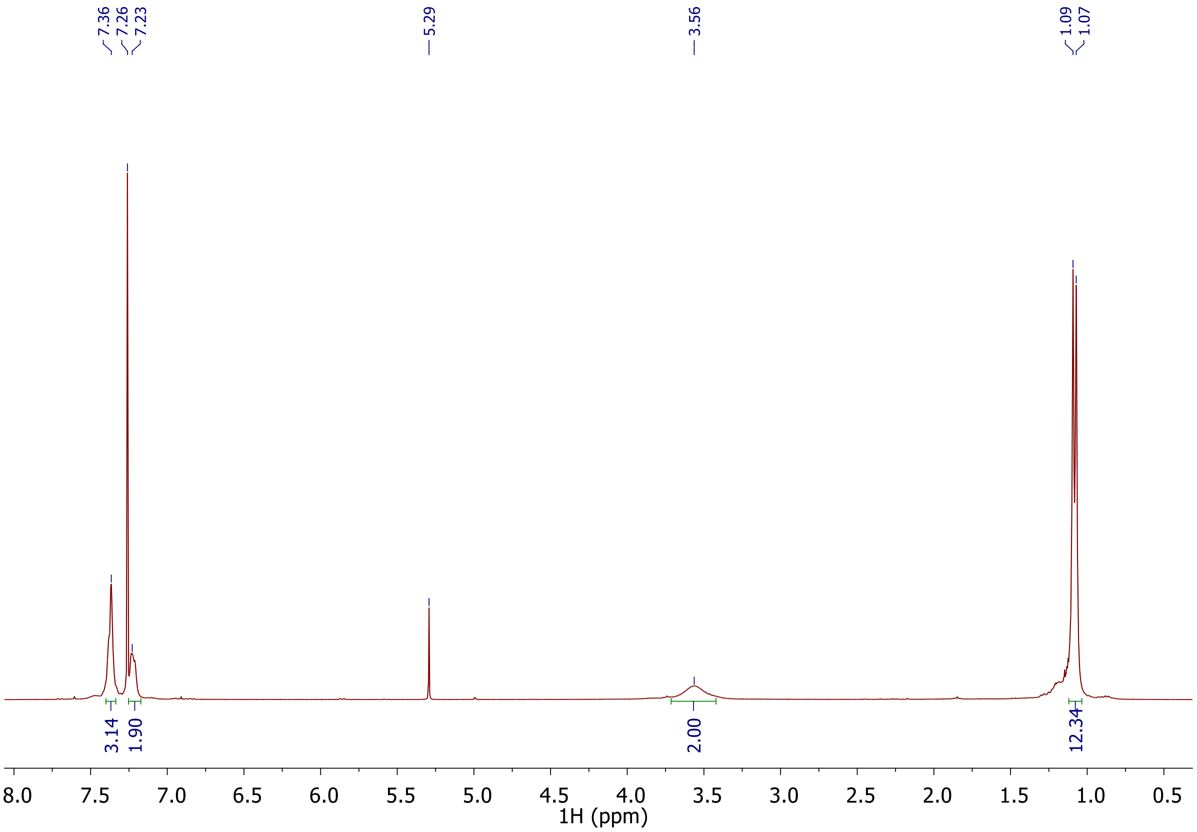

Figure S37: ^1H NMR spectrum (300 MHz, $CDCl_3$, 297K) of N,N'-Bis(diisopropyl)benzimidamide (Dichloromethane: 5.29 ppm).

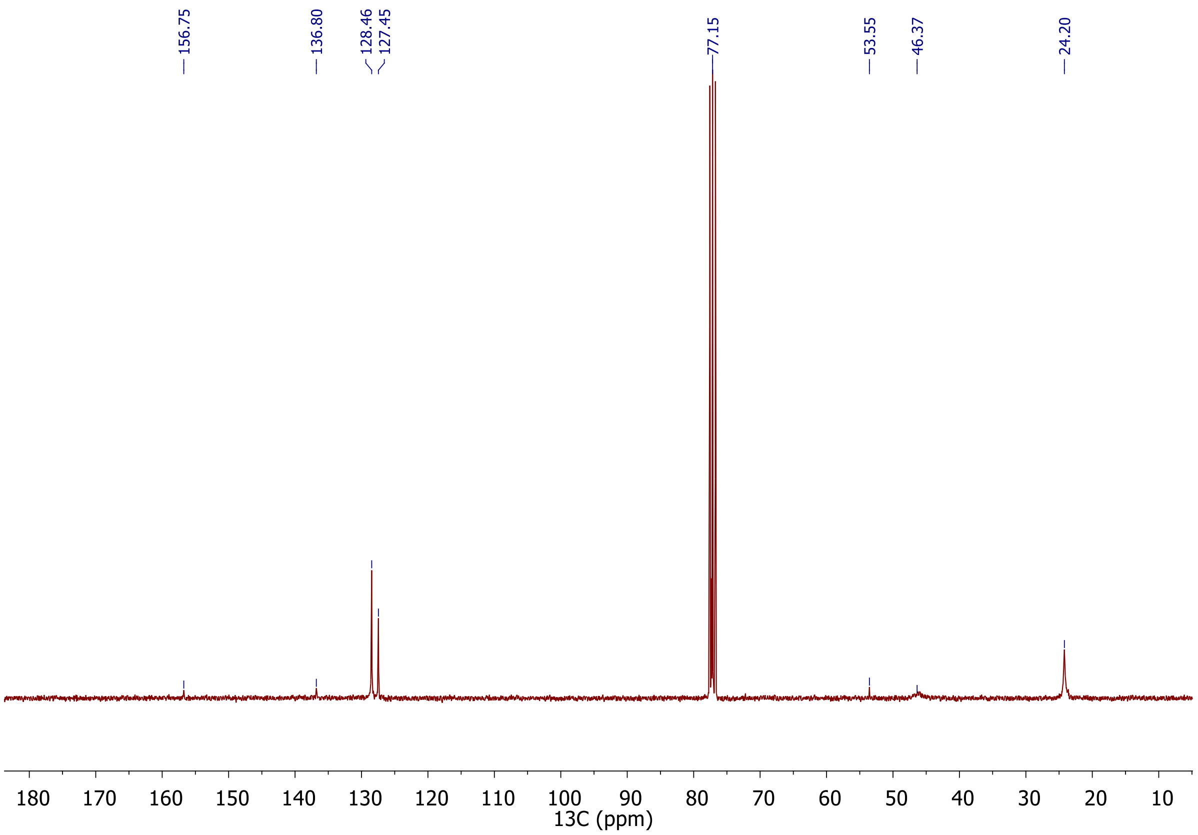

Figure S38: ^{13}C NMR spectrum (75.5 MHz, $CDCl_3$, 297K) of N,N'-Bis(diisopropyl)benzimidamide (Dichloromethane: 53.5 ppm).

2.6.4 Protolysis of [(thf)$_3$Ca{MesC(N-*i*Pr)$_2$}I] (2)

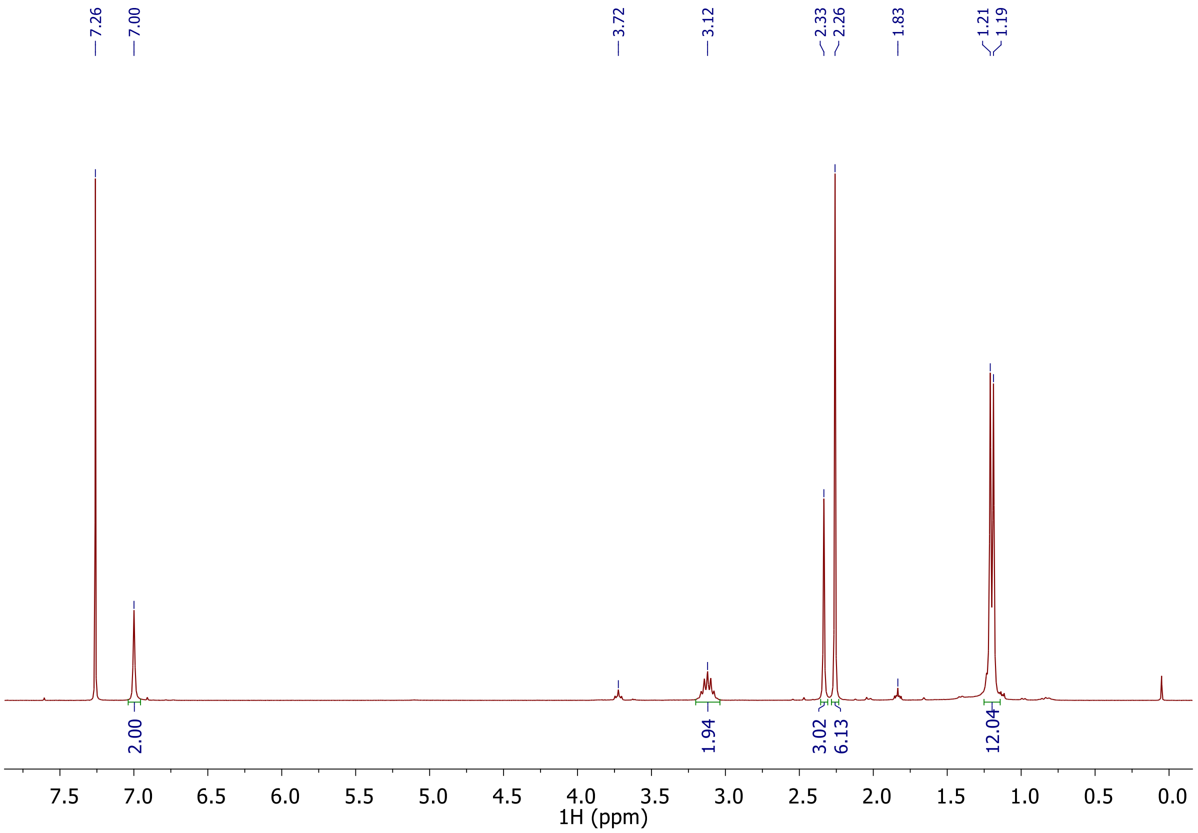

Figure S39: ^{1}H NMR spectrum (300 MHz, $CDCl_3$, 297K) of N,N'-Bis(diisopropyl)-2,4,6-trimethylbenzimidamide (THF: 3.72, 1.83 ppm).

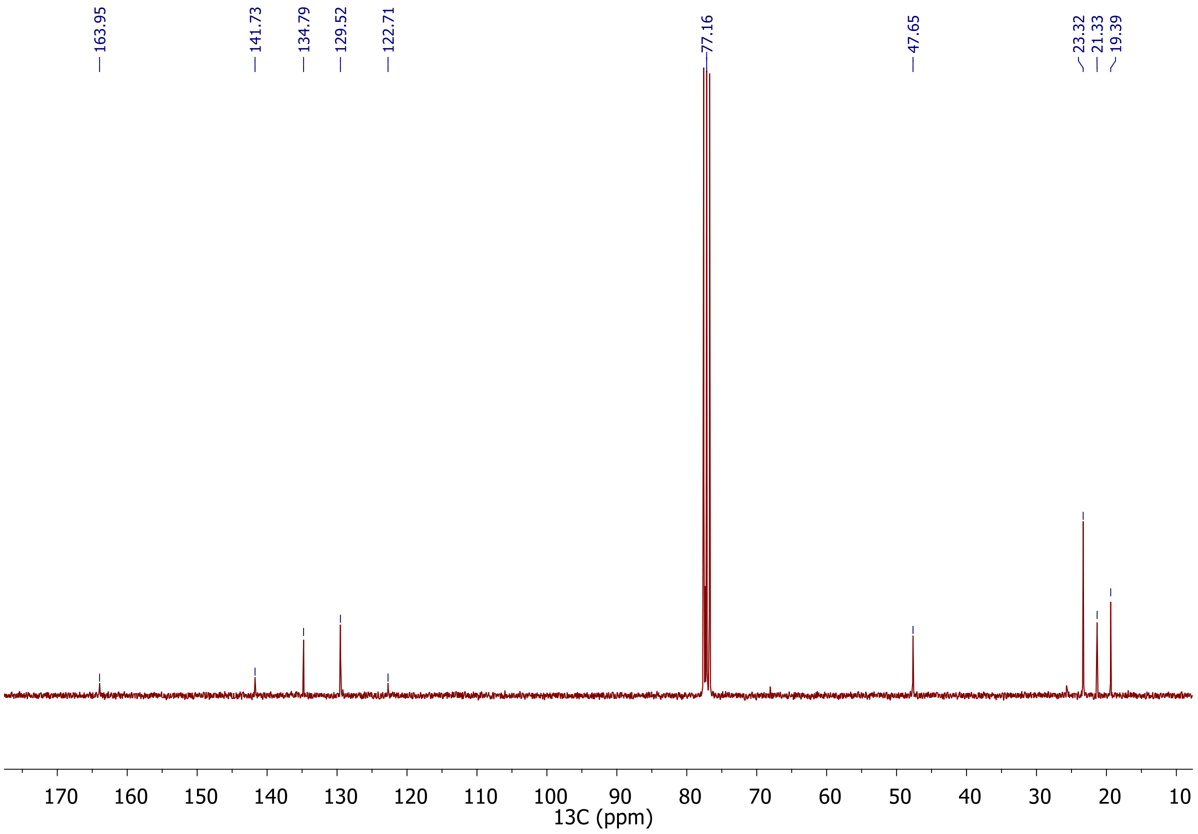

Figure S 40: ^{13}C NMR spectrum (75.5 MHz, $CDCl_3$, 297K) of N,N'-Bis(diisopropyl)-2,4,6-trimethylbenzimidamide.

2.7 Single-crystal X-ray structural analyses

Table S1. Crystal data and details on structure refinement for the reported compounds.

Compound	1a	1b	2	3
CCDC deposition number	2414721	2414722	2414723	2414724
Molecular formula sum	$C_{34}H_{78}Br_2Ca_2N_4O_4Si_4$	$C_{34}H_{78}Ca_2I_2N_4O_4Si_4$	$C_{28}H_{49}CaIN_2O_3$	$C_{68}H_{82}CaN_2O_3$
Formula weight / g mol^{-1}	959.34	1053.32	628.67	1015.43
Crystal system	monoclinic	monoclinic	triclinic	triclinic
Space group	$P2_1/c$	$P2_1/c$	$P\bar{1}$	$P\bar{1}$
Cell metric *a* / Å	13.212(2)	13.516(2)	8.488(2)	14.220(2)
b / Å	14.602(2)	14.860(2)	9.915(2)	14.921(3)
c / Å	13.231(2)	13.059(2)	19.207(4)	19.062(3)
α / deg.	90	90	80.614(5)	70.739(5)
β / deg.	102.305(5)	101.621(5)	77.977(6)	80.612(5)
γ / deg.	90	90	89.323(5)	61.891(4)
Cell volume / Å^3	2493.8(7)	2569.1(7)	1559.5(5)	3367.4(10)
Molecules per cell *z*	2	2	2	2
Electrons per cell F_{000}	1016	1088	656	1096
Calcd. density ρ / g cm^{-3}	1.278	1.362	1.339	1.001
μ / mm^{-1} (Mo-K_α)	1.962	1.551	1.220	0.134
Crystal shape and color	colorless plate	colorless prism	colorless rod	yellow prism
Crystal size / mm	0.21×0.08×0.03	0.19×0.13×0.09	0.21×0.05×0.03	0.17×0.17×0.11
θ range / deg.	2.104 … 28.589	2.060 … 30.559	2.189 … 25.027	1.617 … 27.103
Reflections collected	29715	38754	13291	36090
Reflections unique	6335	7721	5481	14850
Reflections with $I>2\sigma(I)$	4461	5516	3633	9448
Completeness of dataset	100 %	100 %	99.5 %	100 %
R_{int}	0.0576	0.0597	0.0519	0.0452
Data; Parameters; Restraints	6335; 271; 63	7721; 243; 14	5481; 452; 290	14850; 672; 0
R_1 (all data, $I>2\sigma(I)$)	0.0737; 0.0385	0.0660; 0.0357	0.1287; 0.0784	0.0990; 0.0588
wR_2 (all data, $I>2\sigma(I)$)	0.0742; 0.0652	0.0791; 0.0674	0.1802; 0.1602	0.1641; 0.1429
GooF (F^2)	1.015	1.016	1.071	1.037
Max. residual peaks	–0.457; 0.424	–0.407; 0.681	–0.990; 1.546	–0.425; 0.929

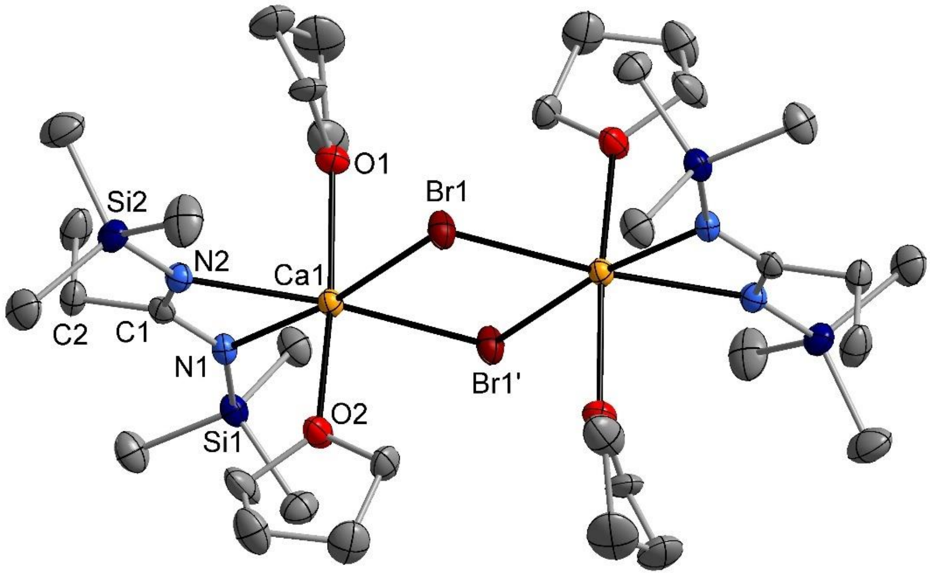

*Figure S41: Molecular structure and atom labeling scheme of **1a**. Ellipsoids represent a probability of 50%, hydrogen atoms are neglected for clarity reasons. Symmetry-equivalent atoms (1-x, 1-y, 1-z) are marked with an apostrophe. Selected bond lengths of **1a** (pm): Ca1-N1 239.3(2), Ca1-N2 239.9(2), Ca1-Br1 291.58(6), Ca1-Br1' 291.32(6), Ca1-O1 240.2(2), Ca1-O2 234.6(2), C1-N1 132.3(3), C1-N2 133.7(3), C1-C2 152.3(3), N1-Si1 170.9(2), N2-Si2 170.7(2). Bond angles (deg): N1-Ca1-N2 57.15(6), Ca1-N1-C1 91.8(1), C1-N1-C2 119.0(2), Ca1-N2-C1 91.2(1).*
